# *Para*-substituted benzoic acid ruthenium(ii) complexes: structural features modulating cytotoxicity

**DOI:** 10.1039/d5ra07271a

**Published:** 2026-04-16

**Authors:** Jocely L. Dutra, Pedro H. S. Marcon, Gustavo Moselli, Fabiano M. Niquini, João Victor F. da Costa, Carlos André F. Moraes, Ataualpa A. C. Braga, Javier Ellena, Alzir A. Batista, João Honorato de Araujo-Neto

**Affiliations:** a Departament of Chemistry, Universidade Federal de São Carlos (UFSCar) 13561-905 São Carlos Brazil; b Department of Fundamental Chemistry, Institute of Chemistry, University of São Paulo São Paulo 05508-000 Brazil joaohonorato@iq.usp.br; c Instituto de Física de São Carlos, Universidade de São Paulo (USP) CP 369, CEP 13560-970 São Carlos Brazil

## Abstract

This work describes the synthesis of six new ruthenium(ii) complexes bearing *para*-substituted benzoic acids of general formula [Ru(L)(dppb)(bipy)]PF_6_, where L = terephthalic acid (L-CO_2_H), 4-(chloromethyl)benzoic acid (L-CCl), 4-(bromomethyl)benzoic acid (L-CBr), 4-(amino)benzoic acid (L-NH_2_), and 4-(nitro)benzoic acid (L-NO_2_), bipy = 2,2′-bipyridine and dppb = 1,4-bis(diphenylphosphino)butane. The complexes were characterized by elemental analysis, molar conductivity, NMR, cyclic voltammetry, IR spectroscopy and, for selected compounds, single-crystal X-ray diffraction. The binuclear complex RuBi exhibited a differentiated structural and spectroscopic pattern, including solvent-dependent ^31^P NMR signal duplication associated with the coexistence of closely related conformers, as supported by DFT calculations. Electrochemical investigations revealed Ru^2+^/Ru^3+^ redox couples whose *E*_1/2_ values strongly depend on the electronic nature of the para substituent, following the trend NO_2_ > COOH > CH_2_Br ≈ CH_2_Cl > NH_2_. The strongly electron-donating –NH_2_ group significantly lowers the oxidation potential and introduces an additional ligand-centered oxidation process, highlighting the pronounced electronic modulation imposed by this substituent. The *in vitro* cytotoxicity of the complexes, free ligands, and cisplatin was evaluated against MDA-MB-231 (breast), A549 (lung), A2780 (ovarian), A2780cis (cisplatin-resistant ovarian), and MRC-5 (non-tumor lung) cell lines using the MTT assay. All ruthenium complexes were more cytotoxic than the corresponding free ligands and cisplatin. Among them, [Ru(L-NH_2_)(dppb)(bipy)]PF_6_ (RuNH_2_) stood out, exhibiting a submicromolar IC_50_ value (0.5 ± 0.1 µM) against A2780 cells and the highest selectivity index (3.6) of the series. Its superior performance can be correlated with the strong donor character of the –NH_2_ group, which modulates the redox properties of the metal center and enhances hydrogen-bonding capability, potentially favoring stronger biological interactions. RuNH_2_ was further investigated in advanced biological assays, showing pronounced morphological alterations, significant reduction in clonogenic survival of A2780 cells, and concentration-dependent accumulation in the sub-G1 phase, consistent with induction of cell death. Finally, all complexes, except RuNH_2_ due to its intrinsic fluorescence, were evaluated for interaction with human serum albumin, revealing moderate binding affinities compatible with bloodstream transport. Collectively, these findings demonstrate how subtle electronic effects govern redox behavior and cytotoxic performance, highlighting RuNH_2_ as a promising candidate for ovarian cancer therapy.

## Abstract 2

Electronic fine-tuning of Ru(ii) complexes drives redox behavior and anticancer activity, with RuNH_2_ emerging as a highly selective, submicromolar candidate that outperforms cisplatin and stands out as a promising agent for ovarian cancer therapy.

## Graphical abstract

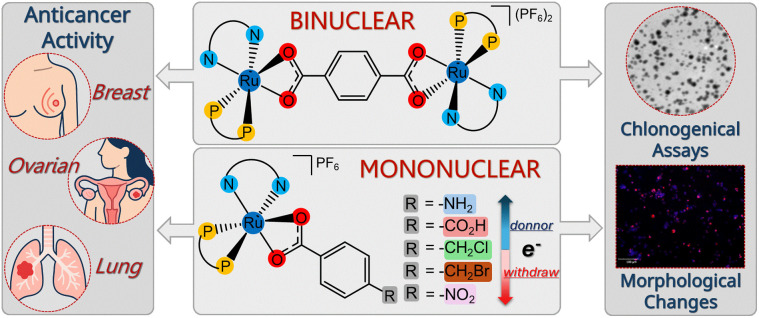

## Introduction

Cancer remains a major public health concern, ranking as the second leading cause of death worldwide.^1,2^ Although platinum-based chemotherapeutics such as cisplatin, carboplatin, and oxaliplatin have revolutionized cancer treatment, their clinical use is limited by severe dose-dependent side effects.^3^ Patients undergoing such treatments may experience over 40 distinct adverse effects, including severe nausea, vomiting, and tissue damage, such as neurotoxicity and nephrotoxicity.^4–6^ These problems, combined with the development of resistance, underscore the urgent need for alternative metal-based agents with improved efficacy and safety profiles. Ruthenium-based complexes have emerged as a promising class of agents with significant anticancer potential. Since the development of metal-based salts such as NAMI-A and KP1339, which demonstrated notable antitumor, antimetastatic, and anti-angiogenic activities in preclinical studies, numerous new molecules have been synthesized, and their pharmacological potential has been extensively explored.^7^ Currently, clinical trials are evaluating the antitumor activity of these complexes in combination with other drugs already used in chemotherapy. For example, the candidate BOLD-100® (proposed trade name for NKP1339) has been tested in humans for the treatment of bile duct, colon, pancreatic, and gastric cancers.^8,9^ The ruthenium(ii) complex TLD1433 also has advanced to clinical evaluation as a photosensitizer for photodynamic therapy in bladder cancer.^10^

Benzoic acids and their derivatives are part of simple phenolic acids and are known for their pharmacological action, such as antibacterial and antifungal.^11,12^ When coordinated to ruthenium, these molecules can significantly influence the structural and electronic properties of organometallic and coordination complexes, allowing precise modulation of their chemical and physical features, as well as their stability and reactivity.^13–15^ Recently, a study demonstrated that Ru(ii)/benzoate complexes exhibited significant activity against triple-negative breast cancer cells. In this study, the interaction of the complexes with DNA was also investigated, and the experiments suggested that the compounds do not significantly alter its secondary structure, indicating that DNA is not the primary target.^13^

**Fig. 1:**
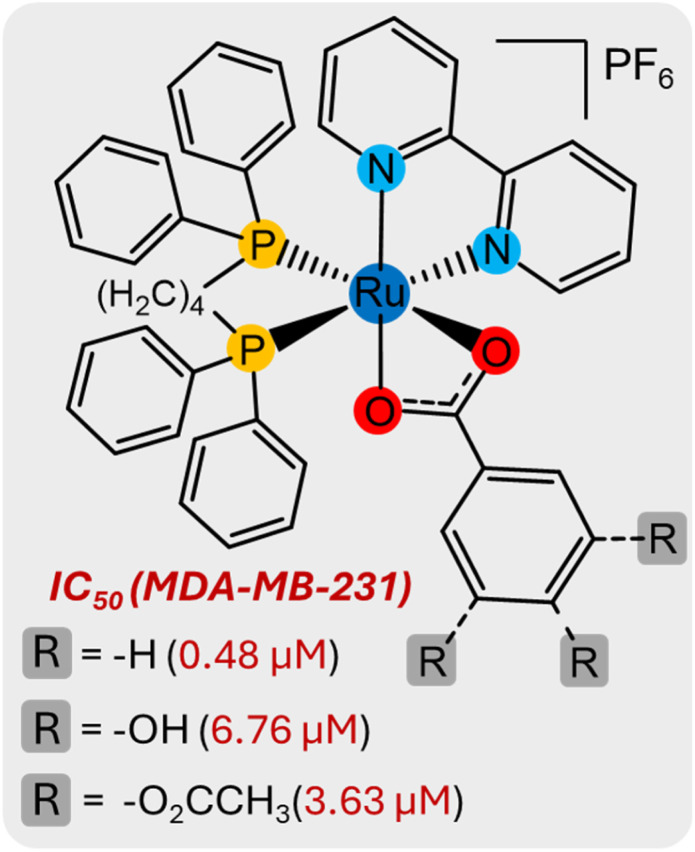
Representative structure of Ru(ii)/benzoate complexes described in the literature.

In a previous study, our research group developed a series of monocationic Ru(ii)/bipyridine/biphosphine complexes containing benzoic acid, gallic acid, and esterified gallic acid ligands (Fig. 1). Their cytotoxicity was found to correlate with lipophilicity, with the benzoic acid derivative (without substitutions) being the most active, displaying a potency 21-fold higher than cisplatin, while the corresponding gallic acid complex showed comparatively lower activity.^16^ In another study, four new Ru(ii)/arene complexes with *p*-substituted benzoic acids, including one binuclear complex, were synthesized and characterized. These compounds followed the same trend observed previously, with the unsubstituted benzoic acid derivative being more active than its *p*-nitro analogue, while the binuclear terephthalic acid complex also displayed low activity. Notably, one of the complexes exhibited enhanced selectivity toward the MDA-MB-231 breast cancer cell line.^14^

**Scheme 1:**
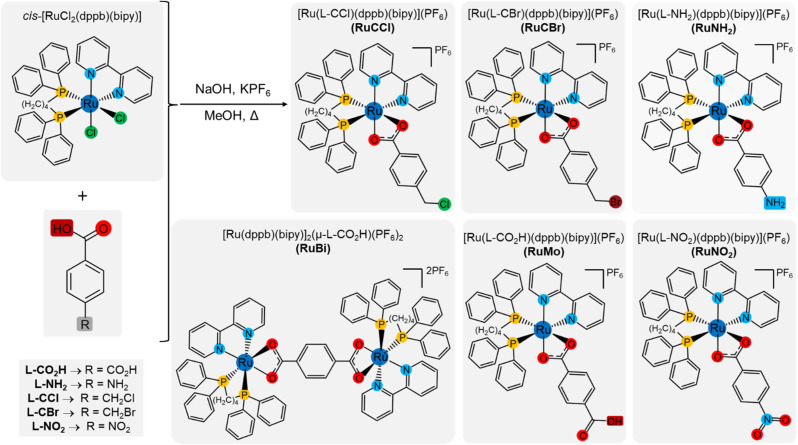
Representative scheme of the reaction path of the Ru(ii) complexes with *para*-substituted benzoic acid ligands.

Building upon these findings, the present study was designed to systematically evaluate how electronic effects introduced by *para*-substituted benzoic acids modulate the structural, redox, and biological properties of Ru(ii)/bipyridine/bisphosphine systems (Scheme 1). To this end, a new series of mononuclear and binuclear complexes incorporating dppb and bipy ligands was developed and thoroughly characterized by spectroscopic, electrochemical, and crystallographic techniques. Their antiproliferative activity was investigated against breast, lung, ovarian, and cisplatin-resistant ovarian cancer cell lines, as well as non-tumor fibroblasts, to establish structure–activity relationships within this family. Additional mechanistic studies, including morphological analysis, clonogenic assays, cell cycle evaluation, and serum albumin binding experiments, were performed to gain deeper insight into the biological behaviour of the most promising derivative.

## Results and discussion, experimental

### Synthesis and characterization

The coordination of *para*-substituted benzoic acids with the Ru(ii) precursor *cis*-[RuCl_2_(dppb)(bipy)], through the substitution of the chloride ligands (Scheme 1), resulted in six new complexes: a binuclear with formula [Ru(dppb)(bipy)]_2_(µ-L-CO_2_H)(PF_6_)_2_ and five mononuclear complexes of general formula [Ru(L)(dppb)(bipy)]PF_6_, where L = terephthalic acid (L-CO_2_H), 4-(chloromethyl)benzoic acid (L-CCl), 4-(bromomethyl)benzoic acid (L-CBr), 4-(amino)benzoic acid (L-NH_2_) and 4-(nitro)benzoic acid (L-NO_2_). The complex was synthesized under basic conditions, where coordination occurs through substitution of the chloride ligands by the oxygen atoms of the carboxylate group, following well-established ligand exchange procedures reported for Ru complexes in the literature.^17^ Molar conductance measurement of the complexes solutions (1 mM) indicates 1 : 1 electrolyte in dichloromethane or DMSO at 25 °C, confirming the obtention of proposed structures with the substitution of the two chloride atoms by the monoanionic ligands, resulting in the monocationic complexes with PF_6_^−^ counterion. The elemental analysis, as detailed in the experimental section, demonstrates the purity of the complexes powder. The high resolution ESI-MS spectra of complexes RuBi, RuMo, RuNH_2_, RuNO_2_, RuCCl, and RuCBr (Fig. S1–S6) show peaks of the molecular ions (simulated fragment values in parenthesis) [Ru(dppb)(bipy)]_2_(µ_2_-L-CO_2_H)]^2+^, [Ru(L-CO_2_H)(dppb)(bipy)]^+^, [Ru(L-NH_2_)(dppb)(bipy)]^+^, [Ru(L-NO)(dppb)(bipy)]^+^, [Ru(L-CCl)(dppb)(bipy)]^+^ and [Ru(L-CBr)(dppb)(bipy)]^+^ at *m*/*z* 766.1473 (766.1446), 849.1595 (849.1592), 820.1811 (820.1790), 850.1539 (850.1532), 853.1455 (853.1448) and 899.0974 (899.0948), respectively.

**Fig. 2:**
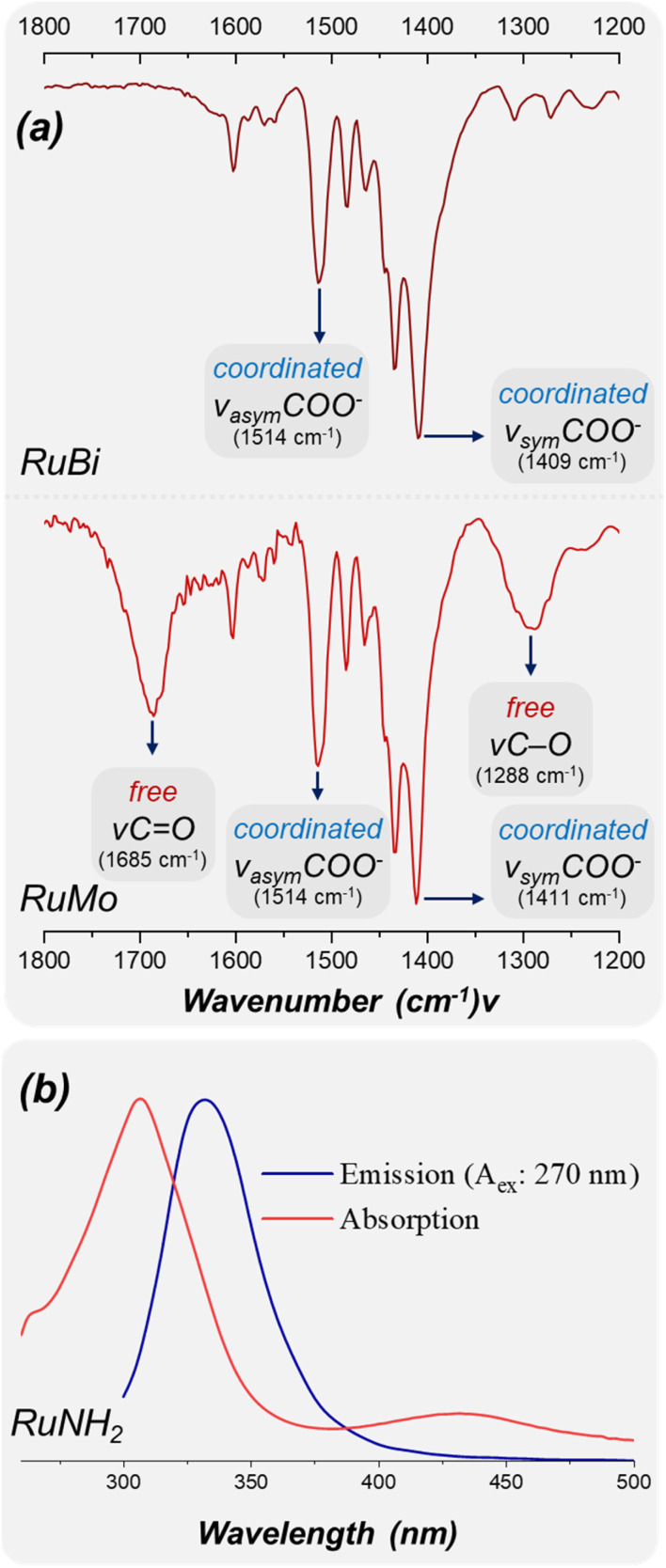
(a)Infrared spectrum of complexes RuBi e RuMo highlighting the carboxylates stretches. (b) UV-vis absorption (red) and fluorescence emission (blue) spectrum of ethanol solution of complex RuNH_2_.

Infrared vibrational spectroscopy was performed for all complexes and their corresponding deprotonated ligands. The spectra (Fig. S7–S12) reveal that the free deprotonated ligands exhibit the characteristic stretching modes of the carboxylate acid group: *ν*_asym_(COO^−^) at ∼1550 cm^−1^, *ν*_sym_(COO^−^) at ∼1400 cm^−1^.^18^ The asymmetric and symmetric stretching modes of the coordinated carboxylate group are clearly observed and summarized in Table S1. Notably, the shifts in *ν*_asym_(COO^−^) and *ν*_sym_(COO^−^), together with the decrease in Δ*ν* (Δ*ν*_ligand_ > Δ*ν*_complex_), support a bidentate chelating coordination mode through both oxygen atoms of the anionic carboxylate group, forming a new four-membered ring.^19–21^ Since L-CO_2_H ligand contains two carboxylic acid moyeties, its mononuclear complex (RuMo) is the only structure to retain the free ligand's carboxylic acid stretching vibrations, while showing the asymmetric and symmetric shift of the coordinated COO^−^ group, suggesting that only one of the carboxylates is responsible for coordination (Fig. 2a). For instance, the binuclear complex (RuBi) presents the same –COO^−^ characteristic group although lacking the original *ν*CO, *ν*C=O, *ν*O–H, *ν*_asym_(COO) and *ν*_sym_(COO), thus indicating that the coordination occurs by both carboxylate groups. We also attributed the bands at ∼520-500 cm^−1^ as *ν*(Ru–P) and the strong bands arround 840 and 557 cm^−1^ as *ν*(P–F) from the PF_6_^−^ counterions.

The photophysical studies showed that RuNH_2_ was the only complex in the series to exhibit detectable fluorescence, displaying an emission band centred at 330 nm upon excitation at 270 nm (Fig. 2b), while the remaining complexes were non-emissive under identical conditions. The absorption spectrum of RuNH_2_ also presents a band around 430 nm, consistent with a Ru(ii) → diimine metal-to-ligand charge transfer (MLCT) transition. Instead, the emission at 330 nm matches well with the characteristic ligand-centered fluorescence of *para*-aminobenzoic acid, which typically emits in the 330–360 nm region depending on the solvent environment.^22,23^ The persistence of this UV emission upon coordination indicates that the excited state retains ligand-centred character. In the excited state, this electronic enrichment may facilitate intramolecular photoinduced electron transfer (PET) processes involving the aniline moiety, which can efficiently compete with and quench the emissive MLCT state.^23,24^ This provides a coherent explanation for the presence of MLCT absorption alongside exclusive ligand-centred emission in RuNH_2_.

The strongly electron-donating –NH_2_ substituent of RuNH_2_ complex enhances electron density across the aromatic framework and modulates the electronic structure of the complex, as also reflected in the lowered Ru^2+^/Ru^3+^ oxidation potential, as will be discussed in the following paragraph.

**Fig. 3:**
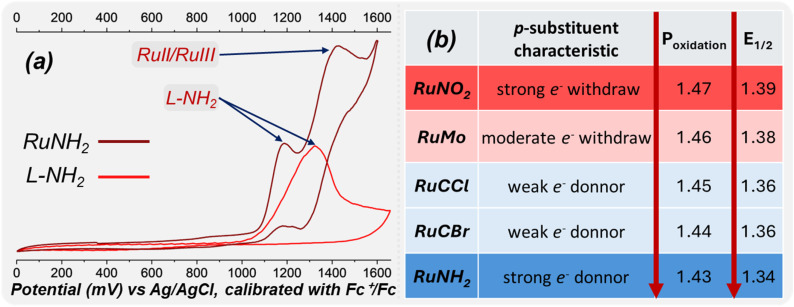
(a) Cyclic voltammogram of the complex RuNH_2_ and the ligand L-NH_2_ in CH_2_Cl_2_ (0.1 M [nBu_4_N]PF_6_) using Ag/AgCl (calibrated with Fc^+^/Fc) as the reference electrode. (b) Electrochemical parameters derived from cyclic voltammetry experiments, showing the oxidation potentials (Pox) and half-wave potentials (*E*_1/2_) of the investigated complexes.

The cyclic voltammograms of the complexes exhibit Ru^2+^/Ru^3+^ redox features with both anodic and cathodic peaks, although with significant peak separation (Δ*E*_p_ ≈ 150 mV – Fig. S29), whose *E*_1_/_2_ values show a clear dependence on the electronic nature of the substituent bound to the ancillary ligand (Fig. 3b). A systematic trend is observed: the strongly electron-donating –NH_2_ group (RuNH_2_ complex) leads to the lowest oxidation potential and half-wave potential, whereas the strongly electron-withdrawing –NO_2_ substituent (RuNO_2_ complex) shifts the Ru^2+^/Ru^3+^ couple to significantly higher and half-wave potential. Intermediate values are found for RuMo, RuCCl and RuCBr, with the respective *p*-substituents –COOH (1.377 V), –CH_2_Cl (1.360 V), and –CH_2_Br (1.365 V), consistent with their moderate electron-withdrawing character.

The RuNH_2_ complex deviates markedly by displaying an additional ligand-centered oxidation at *ca.* 1.20 V, a feature also presents in the free ligand (Fig. 3a). The presence of an additional irreversible ligand-centred oxidation in RuNH_2_ likely perturbs the electronic structure of the complex and may contribute to the loss of reversibility of the Ru^2+^/Ru^3+^ couple. The strong donating character of the –NH_2_ group enhances metal/ligand electronic communication, and oxidation of the ligand framework may trigger follow-up chemical processes that prevent the electrochemical regeneration of Ru^2+^ during the reverse scan. These effects are attributed to the strong donation of the –NH_2_ group, which significantly increases electron density in the ligand framework and perturbs the mixing between metal/ligand centered orbitals, enabling ligand oxidation pathways that are not accessible for the other substituents.

Taken together, the trend in *E*_1/2_ values (NO_2_ > COOH > CH_2_Br ≈ CH_2_Cl > NH_2_) demonstrates how subtle electronic modifications in the coligands modulate the redox potential of the Ru^2+^ center (Fig. 3b). RuNH_2_ represents an extreme case in which strong electron donation not only lowers *E*_1/2_ but also introduces distinct redox processes, underscoring the sensitivity of Ru^2+^/Ru^3+^ electrochemistry to substituent effects.

1D (^1^H, ^13^C and ^31^P{^1^H}) and 2D (^1^H–^1^H COSY, ^1^H–^13^C HSQC and ^1^H–^13^C HMBC) NMR spectroscopy experiments were used to further characterize the molecular structures of the obtained complexes (Fig. S13–S27). The ^1^H NMR spectra can be divided into two common regions for all complexes, regarding chemical shift (*δ*) values for bipy, dppb and the main ligands's aromatic hydrogen atoms (1) and the dppb's aliphatic hydrogen atoms (2). The first set of peaks can be found in the region of *δ* 8.6–4.6 ppm. Overall, the most unshielded signals were attributed to one of bipyridine's hydrogen atoms (Ha) and the adjacent sequence of peaks upfield refers to the remaining bipy's H atoms, as well as diphosphine's phenyl ring H atoms and the four aromatic hydrogens located on the benzoic acid group of the ligands. A common observation in all spectra is the absence of the carboxylic groups' acid proton, indicating its anionic coordination mode, even for RuMo, since the other acidic proton could be involved in hydrogen bonding or rapid exchanges in solution, weakeing the observed signal. The major differences in this region of the spectra are the presence of a singlet at *δ* 5.71 ppm of the terminal amine in RuNH_2_, and the singlets concerning the –CH_2_–X (X = Cl, Br) protons at *δ* 4.72 ppm and *δ* 4.65 ppm in RuCCl and RuCBr, respectively. The second region describes the chemical shift of dppb's *n*-butyl groups and shows the same profile across all complexes.

**Fig. 4:**
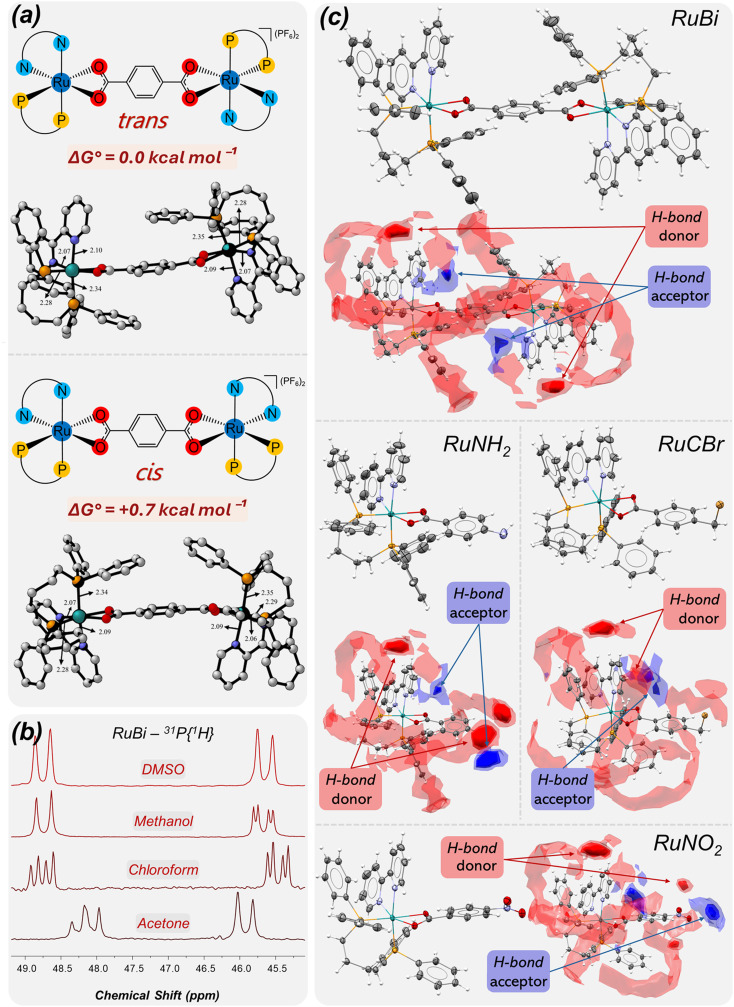
(a) Optimized geometries of the two lowest-energy conformers of the RuBi complex obtained at the B3LYP/def2-SVP level of theory, with single-point energy refinements at the def2-TZVPP level. Left – conformer *trans* (Δ*G*° = 0.0 kcal.mol^−1^) and right – conformer *cis* (Δ*G*° = +0.7 kcal.mol^−1^) key bond distances around the Ru center are indicated (in Å) for each conformer. (b) ^31^P{^1^H} NMR spectra of the RuBi complex obtained in different solvents, illustrating the influence of the solvent on the chemical shifts and overall spectral profile. (c) Molecular structures of complexes RuBi, RuNH_2_, RuCBr, respectively, together with their corresponding full interaction maps (FIMs). Thermal ellipsoids are drawn at the 30% probability level, and PF_6_^−^ counterions are omitted for clarity. In the FIM representation, red isosurfaces indicate regions favorable for hydrogen-bond donors, whereas blue isosurfaces correspond to hydrogen-bond acceptor hotspots within the protein cavity.

The ^31^P{^1^H} NMR spectra provide further insight into the coordination environment of the two *cis*-phosphorus atoms from the dppb ligand. For the mononuclear complexes RuNH_2_, RuMo, RuCCl, RuCBr and RuNO_2_, in chloroform, two non-equivalent doublets are observed at approximately *δ* 48 and *δ* 45 ppm (Fig. S28), corresponding respectively to the phosphorus atoms *trans* to the nitrogen of bipyridine and *trans* to the oxygen of the benzoic acid-type ligands, as typically observed in the literature^25,26^ In contrast, the binuclear RuBi complex exhibits a more complex behaviour that depends markedly on the solvent. In chloroform, both phosphorus doublets undergo additional splitting, resulting in full signal duplication (Fig. 4b). In methanol, however, only one of the doublets remains duplicated while the other coalesces into a single resonance. Conversely, in DMSO, both signals coalesce into two single doublets, resembling the simpler pattern observed for the mononuclear analogues. Notably, the appearance of duplicated phosphorus resonances suggests the presence of more than one chemically similar but magnetically non-equivalent environment in solution. Such behaviour may be rationalized by the coexistence of closely related conformational isomers, potentially arising from different relative orientations of the dppb ligands with respect to the bridging dicarboxylate moiety, leading to two general distinct conformational families, which may be described as *cis*- and *trans*-like orientations across the RuBi molecule (Fig. 4a).

To evaluate this hypothesis and rationalize the solvent-dependent signal duplication detected in the ^31^P NMR spectra, density functional theory (DFT) calculations were performed for the RuBi complex. Among the optimized structures, two conformers were identified as the most stable, differing by only +0.7 kcal mol^−1^. The optimized geometries of these conformers, together with representative distances, are shown in Fig. 4a. In the lowest-energy structure (conformer *trans*), the four phosphorus atoms point away from the plane defined by the dicarboxylate ligand. In contrast, in conformer *cis* the four phosphorus atoms are oriented on the same side of this plane, resulting in a slightly less stable structure. Given that this energy difference of 0.7 kcal mol^−1^ falls within the typical uncertainty of DFT calculations, often estimated to be approximately 1 kcal mol^−1^,^27^ the two conformers are expected to coexist in comparable amounts in solution, thereby contributing similarly to the experimentally observed behavior.

Nuclear magnetic resonance (NMR) calculations using the Gauge-Including Atomic Orbital (GIAO) method^28^ were performed to evaluate the spectral differences between the two conformers. These calculations were carried out at the B3LYP/def2-SVP level of theory, following the same protocol employed for geometry optimizations. The resulting pattern (Fig. S30–S32) qualitatively indicates that up to six distinct phosphorus resonances may arise when contributions from both conformers are considered, with two of these signals exhibiting only small chemical-shift differences. This slight displacement between the corresponding peaks of the two conformers provides a consistent explanation for the duplicated signals observed experimentally for the binuclear complex.

In addition, non-covalent interaction (NCI) analyses were conducted to investigate the presence of π–π stacking interactions between aromatic fragments of the complexes, employing the reduced density gradient (RDG) method as implemented in *NCIplot*.^29^ The NCI results indicate that the presence of an additional π–π interaction provides a significant stabilizing contribution to conformer *trans*, helping to rationalize why this conformer lies lower in energy. The resulting chemical shifts and NCI plots, together with comparative interpretations, are provided in Fig. S33.

Both lines of computational evidence are consistent with the experimental observations and support the conclusion that conformer *trans* is slightly more stabilized than conformer *cis*. Nevertheless, both conformers are expected to be present in appreciable proportions under the experimental conditions and to contribute jointly to the behavior of the analyzed samples.

The crystallographic analysis was performed to elucidate the crystal and molecular structures of the RuNH_2_, RuBi, RuNO_2_, and RuCBr complexes. RuNH_2_ and RuNO_2_ crystallized in the triclinic system, space group *P1̄*, with one independent molecule in the asymmetric unit. RuBi crystallized in the monoclinic system, space group *I*2/*a* and RuCBr crystallized in the monoclinic space group *P*2_1_/*c*, both containing a single molecule in the asymmetric unit (Table S2 contains all crystallographic refinement parameters). The crystal pack of RuNH_2_ and RuBi contained highly disordered solvent molecules, which were treated using a solvent mask during refinement.

The coordination environment around the central Ru(ii) of RuNH_2_, RuBi, RuNO_2_ and RuCBr can be generally described by three ligands acting as bidentate chelates occupying six coordination sites of the metallic center, each assuming a *cis* geometry, as expected (Fig. 4c). In RuNH_2_, the neutral dppb ligand forms a new 7 membered ring through the P_1_–Ru_1_–P_2_ bond, followed by the formation of a 5-membered ring involving the bipyridine ligand *via* N_1_–Ru_1_–N_2_. The 4-aminobenzoate ligand, carrying one negative charge, anchors through O_1_–Ru_1_–O_2_ to generate a strained 4-membered ring, completing the inner coordination sphere. For RuBi, the terephthalic acid ligand (L-CO_2_H), doubly deprotonated, coordinates through both carboxylate groups to distinct ruthenium centers, yielding a binuclear framework stabilized by two 4-membered chelate rings. In RuCBr, the coordination pattern is analogous, with the bromomethylbenzoate ligand anchoring through its deprotonated carboxylate groups, giving rise to a comparable 4-membered chelate ring. We compared the bite angles of the 4-membered carboxylate rings across the three complexes with over 400 related entries in the Cambridge Structural Database and confirmed consistency with expected values (Fig. S34). To balance the charges, equivalent amounts of hexafluorophosphate are present in the outer sphere crystal packing of RuNH_2_ (+1), RuBi (+2), RuNO_2_ (+1) and RuCBr (+1). In all cases, analysis of bond angles indicates distorted octahedral geometries around the Ru(ii) centers, as the observed values deviate from the ideal 90°/180°. Furthermore, the resonant characteristic of the main ligands' coordinated carboxylate groups, as discussed in the FTIR spectroscopic data, match the observed bond lengths: C_1_–O_1_ and C_1_–O_2_ share similar values in RuNH_2_ [1.274(3) Å and 1.275(3) Å] RuBi [1.275(4) Å and 1.270(4)] and RuCBr [1.270(2) Å and 1.270(2) Å], in addition, those values are close to the mean length between C_1_ = O_1_ and C_1_–O_2_ of previously published crystallographic data of the free 4-aminobenzoic acid ligand and free terephthalic acid,^30,31^ indicating the delocalization of electron density over the –COO^−^ group after deprotonation and its preservation after coordination.

In the FIM representation (Fig. 4c), red surfaces correspond to regions favorable for hydrogen-bond donors, whereas blue isosurfaces indicate acceptor-favorable zones. Accordingly, the coordinated carboxylate oxygen atoms preferentially overlap with blue regions, while the –NH_2_ group in RuNH_2_ interacts with blue regions as a donor and regions as acceptor. The –NO_2_ group in RuNO_2_ aligns with blue regions as an acceptor, whereas the –CH_2_Br substituent in RuCBr shows minimal overlap with either hotspot type. These differences in hydrogen-bond complementarity and interaction distribution may contribute to the observed variations in biological activity, as complexes capable of establishing additional directional interactions (*e.g.*, RuNH_2_) are expected to achieve greater stabilization within the binding site compared to derivatives with more limited hydrogen-bonding capacity. In RuBi, the solid-state structure obtained by single-crystal X-ray diffraction provides important structural insight into the origin of the duplicated ^31^P{^1^H} NMR signals. The crystallographic analysis reveals that the complex adopts *trans* conformer relative orientation, which corresponds to the lowest-energy structure identified by DFT calculations.

Although the P–Ru–N angles involving P_1_–Ru–N_2_ and P_1_″–Ru_2_–N_2_″ are equivalent within experimental error, subtle variations in the equatorial bond lengths of the octahedral coordination spheres generate slight electronic differentiation between the two halves of the molecule. These small geometric differences may modulate the electron density around the P2/P2″ nuclei, influencing their magnetic shielding and contributing to the observed signal splitting. Importantly, RuBi crystallizes in the *P1̄* space group with *Z*′ = 1, without an inversion center at a special position, meaning that the two halves of the binuclear unit are not symmetry-related in the solid state. This intrinsic asymmetry is consistent with the inequivalent phosphorus environments detected spectroscopically. In contrast, in a previously reported related complex of the type [Ru(*p*-cymene)(PPh_3_)]_2_(µ–L–CO_2_^−^), the presence of a crystallographic inversion center (*Z*′ = 0.5) enforced equivalence of the two halves, which was reflected in the absence of signal duplication in the ^31^P NMR spectrum.

To evaluate the solution behaviour of the complexes under biologically relevant conditions, ^31^P{^1^H} NMR spectra were recorded over 48 h in DMSO containing 10% (v/v) RPMI medium (Fig. S35–S40). In pure DMSO, all complexes remained spectroscopically unchanged throughout the monitored period, confirming their stability in this coordinating organic solvent. RPMI medium contains significant concentrations of inorganic salts (including NaCl, KCl, CaCl_2_, and MgSO_4_), amino acids, and other bioorganic components capable of weak coordination. RuCBr and RuNO_2_ exhibited no detectable spectral changes over the monitored period, indicating preservation of the original coordination environment. For RuNH_2_, RuCCl, RuMo and RuBi, minor additional phosphorus signals emerged after 24 h; however, their relative integrations remained below 15% of the total phosphorus signal, demonstrating that the parent complexes remain the predominant species in solution (>85%) throughout the experiment.

In the case of RuBi, the newly observed signals at approximately *δ* 30 and 42 ppm indicate the formation of additional phosphorus-containing species over time. Although these signals fall in a region comparable to related Ru(ii) species, the available data do not allow unambiguous structural assignment. These features likely arise from minor speciation or ligand exchange processes influenced by components of the RPMI medium. Importantly, no extensive ligand dissociation or decomposition was observed.

### Biological experiments

The cell viability activities of the ruthenium complexes, ligands and cisplatin were evaluated against tumor cell lines from lung (A549), breast (MDA-MB-231) and ovarian (A2780 and A2780cis), as well as against non-tumor cell line from lung (MRC-5), adopting a conventional tetrazolium colorimetric (3-(4,5-dimethylthiazol-2-yl)-2,5-diphenyltetrazolium bromide (MTT)) method.^33^ The IC_50_ values (half-maximal inhibitory concentration) were determined by the graphs concentration-response curve (Fig. S41–S45), as well as selectivity index (SI) (Table 1).

**Table 1:** *In vitro* cell viability results (IC50 values) for the MDA-MB-231, A549, A2780, A2780cis and MRC-5 cell lines^a^

	IC_50_ (µM)					Selective index (SI)		
	MDA-MB-231	A549	A2780	A2780cis	MRC-5	SI^1^	SI^2^	SI^3^
RuBi	5.45 ± 0.47	>50	2.75 ± 0.05	4.35 ± 0.18	6.06 ± 0.67	1.1	2.2	1.4
RuMo	8.91 ± 0.23	>50	6.98 ± 0.30	7.10 ± 0.63	13.36 ± 1.20	1.5	1.9	1.9
RuNH_2_	1.16 ± 0.12	3.90 ± 0.14	0.56 ± 0.04	10.86 ± 1.85	1.81 ± 0.12	1.5	3.6	—
RuCCl	1.61 ± 0.17	1.67 ± 0.25	0.33 ± 0.05	0.75 ± 0.04	0.61 ± 0.06	—	2.0	—
RuCBr	1.45 ± 0.21	3.79 ± 0.40	1.38 ± 0.36	1.21 ± 0.41	0.54 ± 0.03	—	—	—
RuNO_2_	2.01 ± 0.15	7.86 ± 0.48	2.16 ± 0.20	1.78 ± 0.21	2.79 ± 0.24	1.3	1.2	1.4
L-CO_2_H	≥100	≥100	≥100	≥100	≥100	—	—	—
L-NH_2_	≥100	≥100	≥100	≥100	≥100	—	—	—
L-NO_2_	≥100	≥100	≥100	≥100	≥100	—	—	—
L-CCl	≥100	≥100	30.69 ± 0.33	69.2 ± 4.2	≥100	—	—	—
L-CBr	48.72 ± 3.65	39.46 ± 2.78	10.75 ± 0.70	16.2 ± 1.6	≥100	—	—	—
CB*	12.89 ± 0.88	≥50	9.66 ± 0.02	16.31 ± 0.99	25.84 ± 0.74	2.0	2.7	1.6
Cisplatin^32^	10.20 ± 0.20	14.40 ± 1.40	11.80 ± 0.80	31.8 ± 1.8	29.10 ± 0.80	2.8	2.5	—

a SI^1^ = IC_50_ MRC-5/IC_50_ MDA-MB-231; SI^2^ = IC_50_ MRC-5/IC_50_ A2780; SI^3^ = IC_50_ MRC-5/IC_50_ A2780cis. ^*^c = *cis*-[RuCl_2_(dppb)(bipy)].

The *p*-substituted benzoic acid ligands exhibited, for the most part, IC_50_ values >100 µM, which was the highest concentration tested, except for the L–Br ligand, which showed antitumor activity in all tumor cell lines evaluated. The L–CCl ligand also displayed antitumor activity against ovarian tumor cell lines. However, the synthesized complexes demonstrated higher cytotoxicity than both the free ligands and the reference drug, cisplatin, in the tested cell lines. This suggests that the coordination of *p*-substituted benzoic acids to ruthenium may enhance cytotoxicity, as previously reported in some studies involving benzoic acids.^13,34–36^ Although the free ligand L–CBr exhibited intrinsic antitumor activity across all tumor cell lines tested, its coordination to ruthenium did not potentiate this effect when compared to the other complexes in the series.

This behavior suggests that L–CBr is biologically active in its uncoordinated form, but once bound to the metal center, the ligand may not be sufficiently labilized to exert the same level of cytotoxicity. As a result, the coordination framework may restrict the accessibility of the functional groups responsible for the ligand's intrinsic activity, thereby diminishing its contribution to the overall bioactivity of the complex. Overall, the synthesized complexes exhibited lower IC_50_ values against the A2780 ovarian cancer cell line. When the selectivity index was calculated, the ruthenium complexes also proved to be more selective toward ovarian tissue. Therefore, the RuNH_2_ complex was selected for further in-depth studies, as it showed an IC_50_ of 0.5 ± 0.1 µM approximately 24 times more active than the reference drug and a selectivity index of 3.6, the highest in this series of complexes.

**Fig. 5:**
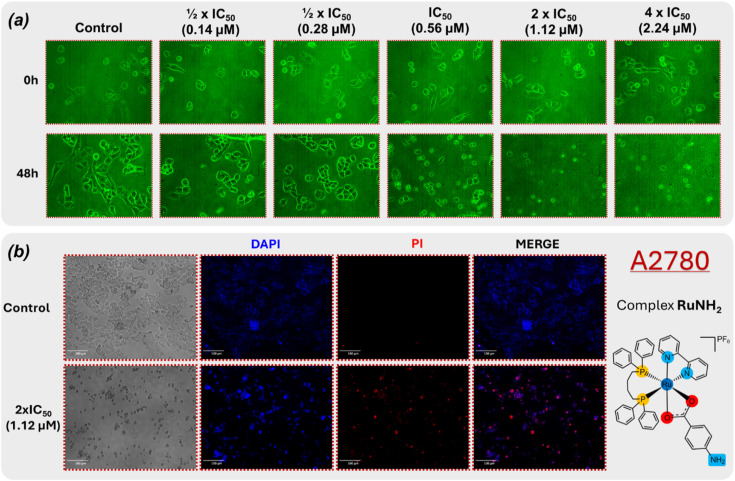
(a) Effect of complex RuNH_2_ on A2780 cell morphology after incubation for 48 h. (b) Fluorescence microscopy of A2780 cells untreated and treated with RuNH_2_ (1.12 µM, 2 × IC_50_), stained with DAPI (blue, total nuclei) and PI (red, dead cells). RuNH_2_-treated cells show increased PI staining compared to control, indicating cell death.

To examine the cytotoxic effects of complex RuNH_2_ in A2780 cells, the cell morphology was analysed over a period of 48 hours of incubation. As shown in Fig. 5a, the untreated cells exhibited normal morphology. On the other hand, A2780 cells treated with RuNH_2_ showed a decrease in cell density, the cells were almost all rounded and detached, starting at the IC_50_ concentration. These morphological alterations indicating cell death.^37,38^

Fluorescence microscopy experiments were conducted using RuNH_2_ at 1.12 µM (corresponding to 2 × IC_50_). After incubation with the complex RuNH_2_, cells were stained with DAPI and PI and imaged using a CELENA® S Digital Imaging System. While DAPI permeates all nuclei, PI selectively stains dead cells.^39^ As shown in Fig. 5b, untreated cells were positive only for DAPI, whereas A2780 cells treated with the RuNH_2_ complex showed a significant proportion of PI-positive cells. These qualitative observations may indicate the induction of cell death through apoptotic pathways, as demonstrated in other studies.^40,41^

To further investigate the cellular effects of the complex RuNH_2_, its impact on colony formation was assessed. The clonogenic assay evaluates the capacity of individual cells to survive treatment, proliferate, and generate colonies consisting of at least 50 cells after drug removal. This approach provides insight into whether the compound exerts a reversible or irreversible effect on cell proliferation.^42–44^

**Fig. 6:**
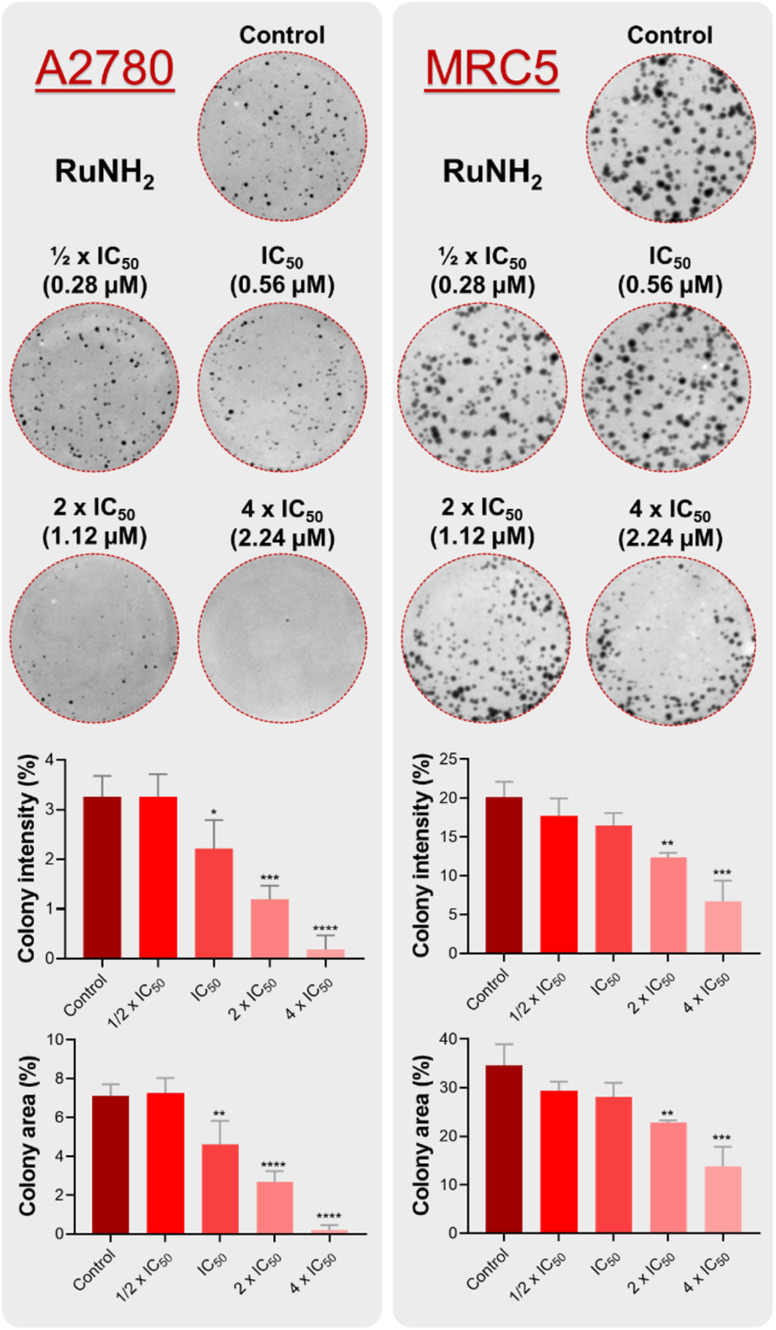
Representative images of the colony formation assay conducted with A2780 or MRC-5 cells after treatment with different concentrations of complex RuNH_2_. Representation of the wells threshold for an experiment is shown along with graph quantifications of colony area and intensity. Data represent the mean ± SD of the assays in triplicate. Significance at the levels of *, *p* < 0.05; **, *p* < 0.01; ***, *p* < 0.001; and ****, *p* < 0.0001 was determined by using ANOVA and Dunnet's test.

We treated the A2780 and MRC-5 cells with different concentrations of complex RuNH_2_, proportional to the IC_50_ in the A2780 cell line, and incubated them with the complex for 48 h. After 10 days, we analysed the resulting colonies. The results presented in Fig. 6 show that, compared with untreated control cells, the number of A2780 colonies decreased drastically starting at the IC_50_ concentration. In contrast, MRC-5 cells treated with the same concentration of RuNH_2_ exhibited only a minor reduction in colony area and density (Fig. 6), with this effect becoming noticeable only at 1.0 µM. Nevertheless, even at the highest concentration tested, colonies of MRC-5 cells were still observed. These findings highlight the selectivity of the RuNH_2_ complex.

**Fig. 7:**
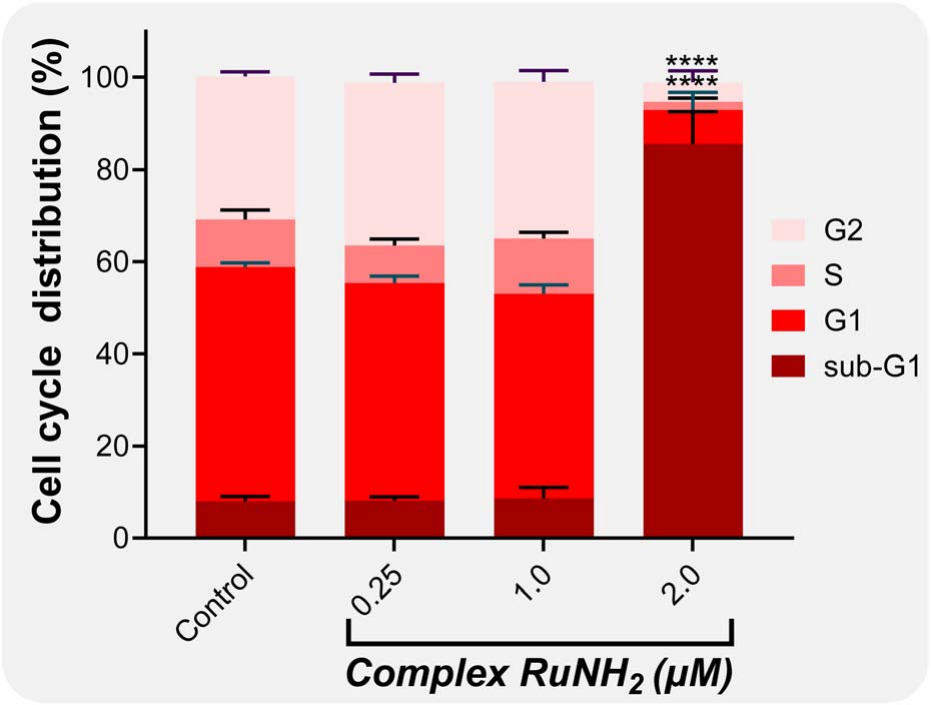
A2780 Cell cycle distribution of cells treated with RuNH_2_ (0.25–2.0 µM) for 48 h, analysed by flow cytometry. The percentages of cells in G1, S, G2 and sub-G1 phases are shown. Data are presented as mean ± SD of three independent experiments. Statistical significance relative to the control: ****p < 0.0001.

The effect of RuNH_2_ on A2780 cell cycle progression was investigated by flow cytometry after treatment with increasing concentrations (0.25–2.0 µM) (Fig. 7). Cells treated with 0.25 and 1.0 µM exhibited a distribution profile comparable to the untreated control, with only minor variations in the G1, S and G2 phases. In contrast, exposure to 2.0 µM resulted in a pronounced and statistically significant increase in the sub-G1 population (****, *p* < 0.0001), concomitant with a marked reduction in the percentages of cells in G1, S and G2 phases. The accumulation of cells in the sub-G1 fraction is consistent with DNA fragmentation and suggests activation of cell death pathways at the highest concentration tested. Overall, RuNH_2_ displays a concentration-dependent effect, with significant disruption of cell cycle distribution observed only at 2.0 µM.

RuNH_2_ displays a clear structure/activity relationship within this series of complexes, in which the strongly electron-donating –NH_2_ substituent probably plays a decisive role. The *σ*-donor character of the amino group lowers the Ru^2+^/Ru^3+^ redox potential and introduces an additional ligand-centered oxidation process, increasing the overall electron density of the complex and potentially enhancing its reactivity toward biological targets.

At the same time, the –NH_2_ functionality provides additional hydrogen-bond donor/acceptor capability, as suggested by FIM analysis, which may favor stronger and more directional interactions within cellular binding sites. Importantly, this electronic and interaction profile correlates with the observed submicromolar IC_50_ in A2780 cells and the highest selectivity index of the series, indicating that the combined electronic modulation and hydrogen-bonding capacity of RuNH_2_ are key contributors to its superior antitumor activity.

### HSA binding experiments

To investigate albumin quenching, the most abundant protein in human plasma, Ru(ii) complexes were studied using human serum albumin (HSA) (Sigma-Aldrich) as a model. All experiments were carried out in triplicate at three different temperatures (298, 303, and 310 K), and the interaction parameters were determined (Table 2).

**Table 2:** Stern–Volmer quenching constant (*K*_sv_, L mol^−1^); biomolecular quenching rate constant (*K*_q_, L mol^−1^ s^−1^); binding constant (*K*_b_, M^−1^); number of binding sites (*n*), Δ*H*° (kJ mol^−1^), Δ*S*° (J mol^−1^ K^−1^), and Δ*G*° (kJ mol^−1^) values for the complex-HSA system at different temperatures

	*T* (*K*)	*K*_SV_ × 10^4^	*K*_q_ × 10^13^	*K*_b_ × 10^4^	*n*	Δ*H*°	Δ*S*°	Δ*G*°
RuBi	298	11.87 ± 0.02	1.91	12.94 ± 0.06	1.09	−1.7	92.1	−29.2
	303	12.39 ± 0.03	2.00	12.80 ± 0.04	1.04		92.1	−29.6
	310	13.31 ± 0.04	2.15	12.31 ± 0.04	0.97		91.9	−30.2
RuMo	298	4.62 ± 0.10	7.33	5.03 ± 0.12	0.99	13.6	135.5	−26.8
	303	4.64 ± 0.12	8.43	5.45 ± 0.05	1.06		135.5	−27.5
	310	5.08 ± 0.02	8.19	5.24 ± 0.05	1.07		134.1	−28.0
RuCCl	298	4.54 ± 0.10	0.73	4.76 ± 0.10	1.38	8.8	118.9	−26.7
	303	4.74 ± 0.21	0.76	5.05 ± 0.25	1.39		118.9	−27.3
	310	4.79 ± 0.08	0.77	5.10 ± 0.05	1.37		118.4	−27.9
RuCBr	298	8.85 ± 0.15	1.43	9.34 ± 0.20	1.07	−19.3	30.4	−28.4
	303	8.82 ± 0.12	1.33	8.21 ± 0.08	0.96		30.4	−28.5
	310	7.87 ± 0.09	1.27	7.73 ± 0.07	0.95		31.4	−29.0
RuNO_2_	298	11.41 ± 0.01	1.84	12.28 ± 0.05	1.01	−19.3	32.5	−29.0
	303	10.72 ± 0.01	1.73	10.80 ± 0.01	1.02		32.5	−29.2
	310	10.34 ± 0.01	1.67	9.78 ± 0.01	0.98		33.1	−29.6

Protein quenching mechanisms typically occur through two distinct pathways, referred to as dynamic and static quenching. Upon excitation at 270 nm, HSA solutions display a fluorescence emission peak at approximately 305 nm (Fig. S46), arising from the intrinsic fluorescence of aromatic residues, predominantly tyrosine with contribution from tryptophan.^21,45^

As shown in Fig. S46, Ru complexes act as albumin quenchers, since the fluorescence emission of HSA decreased with increasing complex concentrations (Fig. S47). The maximum scatter collision-quenching constant values (*k*_q_) for all complexes were on the order of 10^13^ M^−1^ s^−1^ (Table 2), which exceeds the upper limit for dynamic quenching (10^10^ M^−1^ s^−1^), thereby indicating that the predominant mechanism is static quenching.^46^ The calculated *K*_b_ values were in the order of 10^4^–10^5^ M^−1^, indicating that the binding affinity of the complexes for HSA was moderate (Table 2).^21,47-49^

Nevertheless, this did not hinder the complexes from exerting cytotoxic activity, as all of them displayed low IC_50_ values against the evaluated cell lines. Furthermore, the *n* value for the binding sites was approximately 1. The analysis of thermodynamic parameters is a valuable approach for assessing the intermolecular forces governing the interaction between the complexes and HSA.

The interactions of complexes RuBi, RuCBr and RuNO_2_ with HSA are predominantly electrostatic, as indicated by the negative Δ*H*° values and positive Δ*S*° values. For the RuMo and RuCCl complexes, the positive Δ*H* and Δ*S* values suggest that their incorporation into the protein structure is driven by hydrophobic interactions.^50^ The negative values found for the Gibbs free energy variation (Δ*G*°) indicate that the interactions of complex/HSA are spontaneous.

## Conclusions

Six new ruthenium(ii) complexes of general formula [Ru(L)(dppb)(bipy)]PF_6_, bearing para-substituted benzoic acids, were synthesized, fully characterized, and biologically evaluated against breast (MDA-MB-231), lung (A549), ovarian (A2780), and cisplatin-resistant ovarian (A2780cis) tumor cells, as well as non-tumor lung fibroblasts (MRC-5). Structural, spectroscopic, electrochemical, and computational analyses confirmed robust bidentate carboxylate coordination, distorted octahedral geometries, and good stability under biologically relevant conditions. Importantly, systematic variation of the para-substituent produced clear shifts in the Ru^2+^/Ru^3+^ half-wave potentials (NO_2_ > COOH > CH_2_Br ≈ CH_2_Cl > NH_2_), demonstrating effective electronic communication across the coordination sphere and establishing a direct link between ligand electronics and redox behavior.

Coordination to ruthenium significantly enhanced the cytotoxic activity of the ligands, with all complexes outperforming cisplatin *in vitro*, particularly against ovarian cancer cells. While electron-withdrawing and moderately withdrawing substituents afforded active compounds in the low micromolar range, RuNH_2_ emerged as the most promising derivative, exhibiting a submicromolar IC_50_ value (0.5 ± 0.1 µM) against A2780 cells, approximately 24-fold more active than cisplatin, and the highest selectivity index (3.6) within the series. Its superior performance is consistent with a clear structure–activity relationship: the strongly electron-donating –NH_2_ group lowers the Ru^2+^/Ru^3+^ redox potential, introduces an additional ligand-centered oxidation process, increases electron density at the metal center, and provides additional hydrogen-bonding capability. These combined electronic and interaction features likely enhance biomolecular complementarity and biological reactivity.

Morphological and clonogenic assays confirmed that RuNH_2_ induces profound and largely irreversible effects in ovarian tumor cells, with comparatively limited impact on non-tumor MRC-5 cells. Cell cycle analysis further revealed a concentration-dependent response, with significant accumulation in the sub-G1 population at higher concentrations, consistent with DNA fragmentation and activation of cell death pathways. In contrast, the binuclear RuBi complex, despite its structural robustness and conformational richness in solution, did not surpass the mononuclear analogues in cytotoxic performance, indicating that increased nuclearity alone is not a determinant of activity within this scaffold. Moreover, although the free ligand L-CBr displayed intrinsic cytotoxicity, this effect was not enhanced upon coordination, suggesting restricted functional accessibility in the metal-bound form.

Fluorescence and serum albumin binding studies indicated moderate and spontaneous interaction with HSA, without compromising cytotoxicity, supporting preserved bioavailability. Collectively, these results establish a consistent structure–redox–activity relationship within this series and identify RuNH_2_ as a particularly promising candidate for further mechanistic investigation and optimization toward ovarian cancer therapy.

## Experimental

All chemicals used during the synthesis of the complexes are of reagent grade or proportional purity. The solvents used were purified by standard procedures and the chemicals and reactions were handled under argon atmosphere. The reagents RuCl_3_·3H_2_O, 1,4-bis(diphenylphosphino)butane (dppb), 2,2′-bipyridine (bipy), 4-chlorobenzoic acid (HBACl), 4-bromobenzoic acid (HBABr) 4-aminobenzoic acid (HBANH_2_), terephthalic acid (H_2_BATF) and KPF_6_ were purchased from Sigma-Aldrich. The *cis-*[RuCl_2_(dppb)(bipy)] precursor was prepared according to the published procedures.^51^ The spectra in the infrared region (IR) were recorded on a FT-IR Bomem-Michelson MB-102, in the 4000 – 250 cm^−1^ range, using potassium bromide (KBr) pellets. The UV-vis spectra of the complexes were recorded on a spectrometer Hewlett Packard diode array-8452A. Elemental analyses were performed on a FISIONS Instrument EA 1108 CHNS (Thermo Scientific) elemental analyser at the Analytical Laboratory at the Federal University of São Carlos, São Carlos (SP). The conductivity of the complexes in dichloromethane or dimethyl sulfoxide solutions (1.0 mM) was measured on a Meter Lab CDM2300 conductivity meter; a cell of constant 0.089 cm^−1^ was used. ^1^H, ^13^C, {^1^H} and ^31^P{^1^H} NMR spectra of the reported complexes were recorded on a Bruker DRX 400 MHz using the solvents DMSO-*d*_6_ (Dimethyl sulfoxide-*d*_6_). Cyclic voltammetry experiments were carried out at room temperature in a solution of 0.10 M Bu_4_NClO_4_ (TBAP) (Fluka Purum) in CH_2_Cl_2_, with a BAS-100B/W electrochemical analyser (Bioanalytical Systems Inc). A conventional three-electrode configuration was employed, consisting of a platinum disk working electrode, a platinum wire auxiliary electrode, and an Ag/AgCl reference electrode.

### Computational methodology

An initial conformational search was performed using the CREST (Conformer–Rotamer Ensemble Sampling Tool) module within Grimme's xTB program,^52,53^ which enables an efficient exploration of the potential energy surface for ensemble generation. In this stage, implicit solvatation effects were accounted for using the Generalized Born and solvent accessible surface area (GBSA) model with DMSO as solvent, as implemented in the xTB package.^52^ From the conformational ensemble generated by CREST, ten representative structures were selected for subsequent refinement through DFT geometry optimizations.

### X-ray crystallography

The complexes RuNH_2_, RuBi, RuCBr and RuNO_2_ was crystallized from a methanol solution through the slow evaporation of the solvent. Single-crystal X-ray diffraction data were collected using a Rigaku Synergy-S diffractometer equipped with HyPix-6000HE detector and PhotonJet microfocus X-ray sealed tube. The data was collected at 100 K. Unit cell parameters were refined using the CrysAlisPro software suite (CrysAlisPRO, Oxford Diffraction/Agilent Technologies UK Ltd, Yarnton, England). Absorption corrections were applied using the Gaussian method. Structure solution was carried out *via* intrinsic phasing method utilizing the SHELXT program^54^ and refinement with SHELXL least-squares minimization.^55^ H atom positions were calculated with SHELXT's riding atom models while all non-hydrogen atoms were refined with anisotropic displacement parameters. Solvent accessible voids were treated with Solvent Mask tools from OLEX2. Both SHELXT and SHELXL tools were used in the host suite Olex2,^56,57^ also used to make images. The Mercury^58–60^ software was used to make images, calculate distances and angles, as well as the Full Interaction Maps.^61^

### Synthesis

#### Synthesis of the [Ru(dppb)(bipy)]_2_(µ-L-CO_2_H)(PF_6_)_2_ – RuBi

For the synthesis of the binuclear complex reported, a 10 mL methanol solution of 0.065 mmol of the ligand terephthalic acid (L-CO_2_H), was slowly and constantly added to a 10 mL methanol solution with 0.1 g (0.13 mmol) of *cis*-[RuCl_2_(dppb)(bipy)] precursor, under argon atmosphere and constant 50 °C heating. After 10 minutes of stirring, 0.13 mmol of KPF_6_ was also added to the reactional flask. After 4 hours of reaction the solvent was evaporated to around 5 mL and around 10 mL of water was added to precipitate the complex. The solids were filtered off, rinsed and dried similarly to the mononuclear complexes' synthesis.

#### Syntheses of the complexes [Ru(L)(dppb)(bipy)]PF_6_

For the synthesis of the mononuclear complexes, 0.1 g (0.13 mmol) of *cis*-[RuCl_2_(dppb)(bipy)] precursor was dissolved in 20 mL of methanol, under argon atmosphere and constant 50 °C heating. Next, 0.13 mmol of the ligand L (L = terephthalic acid (L-CO_2_H), 4-(chloromethyl)benzoic acid (L-CCl), 4-(bromomethyl)benzoic acid (L-CBr), 4-(nytro)benzoic acid (L-NO_2_) and 4-(amino)benzoic acid (L-NH_2_)) were added. After 10 minutes of stirring, 0.13 mmol of KPF_6_ was also added to the reactional flask. After 4 hours of reaction the solvent was evaporated to around 5 mL and around 10 mL of water was added to precipitate the respective complex. The solids were filtered off and rinsed with water (2 × 10 mL) and ether (2 × 10 mL) and dried under vacuum.

#### [Ru(dppb)(bipy)]_2_(µ–L–CO_2_H)(PF6)_2_ (RuBi)

Orange solid. Yield 60%. Elemental analysis (%) exp. (calc.) C, 55.54 (55.89); H, 4.59 (4.74); N, 3.15 (3.00). Molar conductivity (S cm^2^ mol^−1^): 54.0 (dimethyl sulfoxide) and 49.8 (dichloromethane). Selected IR bands (KBr, cm^−1^): *ν*_asym_(COO) 1483; *ν*_sym_(COO) 1407; *δ*(P–F) 840; *ν*(Ru–P) 518-502; *ν*(Ru–N) 434-411. ESI(+)-MS/MS (*m*/*z*), calc. for C_84_H_76_N_4_O_4_P_4_Ru_2_ [M]+: 766.1446, found: 766.1473. ^1^H NMR (400 MHz, DMSO-*d*_6_, 298 K, *δ*, ppm): 8.51 (2H, *d*, bipy-HA/Ha); 8.23 (6H, 6*x d*, bipy-HI/Hi/HG/Hg/HD/Hd); 7.92 (12H, m, bipy-HC/Hc/HF/Hf; dppb-HPh); 7.68 (7H, m, dppb-HPh); 7.49 (9H, m, bipy-HB/Hb; dppb-HPh); 7.29–7.24 (4H, m, L-CO_2_H-H1/H2/H3/H4); 7.02–6.84 (15H, m, bipy-HH/Hh; dppb-HPh); 6.64 (4H, dd, dppb-HPh); 5.89 (4H, dd, dppb-HPh); 3.17 (2H, m, dppb-aliphatic); 2.75–2.50 (5H, m, dppb-aliphatic); 2.30–1.90 (7H, m, dppb-aliphatic); 1.53 (2H, m, dppb-aliphatic). ^13^C NMR (100.6 MHz, DMSO-*d*_6_, 298 K, *δ*, ppm): 180.67 (L-CO_2_H-C1); 180.57 (L-CO_2_H-C8); 159.99 (bipy-CI/Ci); 158.45 (bipy-CF/Cf); 155.47 (bipy-CE/Ce); 148.72 (bipy-CA/Ca); 138.50 (bipy-CC/Cc); 137.04 (bipy-CY/Cy); 134.00 (L-CO_2_H-C2/C7); 128.91 (bipy-CB/Cb); 126.94 (L-CO_2_H-C3/C4/C5/C6); 125.90 (bipy-CH/Ch); 123.89 (bipy-CD/Cd); 123.18 (bipy-CH/Cg). ^31^P{^1^H} NMR (162 MHz, dichloromethane (using a capillary containing deuterated water), 298 K) *δ* (ppm), (multiplicity): 48.63 (d) and 45.55 (d), ^2^*J* = 34.2 Hz.

#### [Ru(L-CO_2_H)(dppb)(bipy)]PF_6_ (RuMo)

Orange solid. Yield 81%. Elemental analysis (%) exp. (calc.) C, 55.67 (55.59); H, 4.32 (4.16); N, 2.94 (2.82). Molar conductivity (S cm^2^ mol^−1^): 30.2 (dimethyl sulfoxide) and 30.3 (dichloromethane). Selected IR bands (KBr, cm^−1^): *ν*(O–H) 3443; *ν*(C=O) 1687; *ν*_asym_(COO) 1483; *ν*_sym_(COO) 1407; *ν*_sym,free_(COO) 1291; *δ*(P–F) 842; *ν*(Ru–P) 519-501; *ν*(Ru–N) 442-409. ESI(+)-MS/MS (*m*/*z*), calc. for C_46_H_41_N_2_O_4_P_2_Ru [M]+: 849.1595, found: 849.1592. ^1^H NMR (400 MHz, DMSO-*d*_6_, 298 K, *δ*, ppm): 8.51 (1H, *d*, bipy-Ha); 8.23 (3H, 2x d, bipy-Hi/Hg/Hd); 7.96–7.92 (6H, m, bipy-Hc/Hf; dppb-HPh); 7.68–7.47 (8H, m, dppb-HPh; L-CO_2_H-H1/H2); 7.28 (1H, s, L-CO_2_H-H3); 7.24 (1H, s, L-CO_2_H-H4); 7.05–6.80 (7H, m, bipy-Hh; dppb-HPh); 6.64 (2H, dd, dppb-HPh); 5.90 (2H, dd, dppb-HPh); 3.20 (1H, m, dppb-aliphatic); 2.67 (2H, m, dppb-aliphatic); 2.21–1.97 (4H, m, dppb-aliphatic); 1.52 (1H, m, dppb-aliphatic). ^13^C NMR (100.6 MHz, DMSO-*d*_6_, 298 K, *δ*, ppm): 180.63 (L-CO_2_H-C1); 180.55 (L-CO_2_H-C8); 159.96 (bipy-Cj); 158.43 (bipy-Cf); 155.44 (bipy-Ce); 148.70 (bipy-Ca); 138.46 (bipy-*C*c); 137.01 (bipy-Ch); 134.02 (L-CO_2_H-C2/C7); 128.87 (bipy-CB/Cb); 126.91 (L-CO_2_H –C3/C4/C5/C6); 125.68 (bipy-Ci); 123.86 (bipy-Cd); 123.15 (bipy-Cg); 24.21–21.10 (dppb-aliphatic). ^31^P{^1^H} NMR (162 MHz, dichloromethane (using a capillary containing deuterated water), 298 K): *δ* (ppm), (multiplicity): 48.64 (d) and 45.54 (d), ^2^*J* = 34.3 Hz.

#### [Ru(L-NH_2_)(dppb)(bipy)]PF_6_ (RuNH_2_)

Orange solid. Yield 77%. Elemental analysis (%) exp. (calc.) C, 56.23 (56.02); H, 4.51 (4.39); N, 4.72 (4.36). Molar conductivity (S cm^2^ mol^−1^): 28.0 (dimethyl sulfoxide) and 50.2 (dichloromethane). Selected IR bands (KBr, cm^−1^): *ν*(N–H) 3447 + 3378; *ν*_asym_(COO) 1479; *ν*_sym_(COO) 1428; *δ*(P–F) 860; *ν*(Ru–P) 517-509. ESI(+)-MS/MS (*m*/*z*), calc. for C_45_H_42_N_3_O_2_P_2_Ru [M]+: 820.1796, found: 820.1811. ^1^H NMR (400 MHz, DMSO-*d*_6_, 298 K, *δ*, ppm): 8.57 (1H, *d*, bipy-Ha); 8.24–8.14 (3*H*, m, bipy-Hi/Hg/Hd); 8.02–7.87 (6*H*, m, bipy-Hc/Hf; dppb-HPh); 7.66–7.46 (7*H*, m, dppb-HPh); 7.13–6.92 (9H, m, L-NH_2_-H1/H2; bipy-Hh/Hb; dppb-HPh); 6.63 (2H, dd, dppb-HPh); 6.29 (2H, 2*x* s, L-NH_2_-H3/H4); 5.89 (2H, dd, dppb-HPh); 5.71 (2H, s, L-NH_2_-NH_2_); 3.16 (1H, m, dppb-aliphatic); 2.66 (1H, m, dppb-aliphatic); 2.15–1.89 (3H, m, dppb-aliphatic); 1.52 (1H, m, dppb-aliphatic). ^13^C NMR (100.6 MHz, DMSO-*d*_6_, 298 K, *δ*, ppm): 182.81 (L-NH_2_-C1); 159.60 (bipy-Cj); 158.48 (bipy-Cf); 155.45 (bipy-Ce); 148.57 (bipy-Ca); 138.13 (bipy-Cc); 136.61 (bipy-Ch); 133.78 (L-NH_2_-C2/C7); 130.63 (bipy-CB/Cb); 129.41 (L-NH_2_-C3/C4/C5/C6); 125.51 (bipy-Ci); 123.73 (bipy-Cd); 123.02 (bipy-Cg); 26.49–21.09 (dppb-aliphatic). ^31^P{^1^H} NMR (162 MHz, dichloromethane (using a capillary containing deuterated water), 298 K) *δ* (ppm), (multiplicity): 47.68 (d) and 46.71 (d), ^2^*J* = 33.5 Hz.

#### [Ru(L-CCl)(dppb)(bipy)]PF_6_ (RuCCl)

Orange solid. Yield 77%. Elemental analysis (%) exp. (calc.) C, 56.23 (56.02); H, 4.51 (4.39); N, 4.72 (4.36). Molar conductivity (S cm^2^ mol^−1^): 28.0 (dimethyl sulfoxide) and 50.2 (dichloromethane). Selected IR bands (KBr, cm^−1^): *ν*_asym_(COO) 1485; *ν*_sym_(COO) 1436; *δ*(P–F) 843; *ν*(Ru–P) 515-509; *ν*(Ru–N) 430-415. ESI(+)-MS/MS (*m*/*z*), calc. for C_46_H_42_ClN_2_O_2_P_2_Ru [M]+: 853.1455, found: 853.1448. ^1^H NMR (400 MHz, DMSO-*d*_6_, 298 K, *δ*, ppm): 8.58 (1H, *d*, bipy-Ha); 8.24–8.18 (3H, m, bipy-Hi/Hg/Hd); 8.03–7.88 (6H, m, bipy-Hc/Hf; dppb-HPh); 7.69–7.48 (7H, m, dppb-HPh; bipy-Hh/Hb); 7.41–7.28 (4H, dd, L-CCl-H1/H2/H3/H4); 7.06–6.92 (7H, m, dppb-HPh); 6.65 (2H, m, dppb-HPh); 5.91 (2H, m, dppb-HPh); 4.72 (2H, s, L-CCl-H5); 3.18 (1H, m, dppb-aliphatic); 2.70 (3H, m, dppb-aliphatic); 2.25–1.98 (3H, m, dppb-aliphatic); 1.53 (1H, m, dppb-aliphatic). ^13^C NMR (100.6 MHz, DMSO-*d*_6_, 298 K, *δ*, ppm): 181.22 (L-CCl-Cz); 159.95 (bipy-C10); 158.49 (bipy-C6); 155.47 (bipy-C5); 148.73 (bipy-C1); 141.68 (L-CCl-Cf); 138.41 (bipy-C3); 136.95 (bipy-C8); 136.0–126.0 (dppb-CPh); 129.00 (bipy-C2); 128.08 (L-CCl-Cb/Cc); 127.90 (L-CCl-Cd/Ce); 125.65 (bipy-C9); 123.85 (bipy-C4); 123.14 (bipy-C7); 45.39 (L-CCl-*C*m); 26.48–21.22 (dppb-aliphatic). ^31^P{^1^H} NMR (162 MHz, dichloromethane (using a capillary containing deuterated water), 298 K) *δ* (ppm), (multiplicity): 47.68 (d) and 46.71 (d), ^2^*J* = 33.5 Hz.

#### [Ru(L-CBr)(dppb)(bipy)]PF_6_ (RuCBr)

Orange solid. Yield (65%). Elemental analysis (%) exp. (calc.) C, 53.22 (52.99); H, 4.24 (4.06); N, 2.73 (2.69). Molar conductivity (S cm^2^ mol^−1^): 31.2 (dimethyl sulfoxide) and 50.2 (dichloromethane). Selected IR bands (KBr, cm^−1^): *ν*_asym_(COO) 1483; *ν*_sym_(COO) 1429; *δ*(P–F) 842; *ν*(Ru–P) 517-504. ESI(+)-MS/MS (*m*/*z*), calc. for C_46_H_42_BrN_2_O_2_P_2_Ru [M]+: 899.0948, found: 899.0974. ^1^H NMR (400 MHz, DMSO-*d*_6_, 298 K, *δ*, ppm): 8.57 (1H, *d*, bipy-Ha); 8.23 (3H, m, bipy-Hi/Hg/Hd); 8.02–7.95 (6H, m, bipy-Hc/Hf; dppb-HPh); 7.69–7.48 (7H, m, dppb-HPh; bipy-Hh/Hb); 7.36–7.30 (4H, dd, L-CBr -H1/H2/H3/H4); 7.03–6.94 (7H, m, dppb-HPh); 6.65 (2H, m, dppb-HPh); 5.91 (2H, m, dppb-HPh); 4.65 (2H, s, L-CBr -H5); 2.70 (2H, m, dppb-aliphatic); 2.25–1.8 (5H, m, dppb-aliphatic); 1.52 (1H, m, dppb-aliphatic). ^13^C NMR (100.6 MHz, DMSO-*d*_6_, 298 K, *δ*, ppm): 181.16 (L-CBr-Cz); 159.95 (bipy-C10); 158.49 (bipy-C6); 155.46 (bipy-C5); 148.73 (bipy-C1); 142.14 (L-CBr -Cf); 138.39 (bipy-C3); 136.93 (bipy-C8); 136.0–126.96 (dppb-CPh); 128.89 (bipy-C2); 128.56 (L-CBr-Cb/Cc); 127.92 (L-CBr -Cd/Ce); 126.96 (L-CBr-Ca); 125.63 (bipy-C9); 123.83 (bipy-C4); 123.12 (bipy-C7); 44.9 (L-CBr -*C*m); 27.0–20.00 (dppb-aliphatic). ^31^P{^1^H} NMR (162 MHz, dichloromethane (using a capillary containing deuterated water), 298 K) *δ* (ppm), (multiplicity): 49.18 (d) and 46.43 (d), ^2^*J* = 34.3 Hz.

#### [Ru(L-NO_2_)(dppb)(bipy)]PF_6_ (RuNO_2_)

Dark-Orange solid. Yield (78%). Elemental analysis (%) exp. (calc.) C, 54.86. (54.73); H, 4.64 (4.42); N, 4.29 (4.06). Molar conductivity (S cm^2^ mol^−1^): 34.2 (dimethyl sulfoxide) and 52.2 (dichloromethane). Selected IR bands (KBr, cm^−1^): *ν*_asym_(COO) 1531; *ν*_sym_(COO) 1433; *ν*(P–F) 843; *δ*(PF_6_) 557. ESI(+)-MS/MS (*m*/*z*), calc. for C_45_H_40_N_3_O_4_P_2_Ru [M]+:850.1532, found: 850.1539. ^1^H NMR (400 MHz, DMSO-*d*_6_, 298 K, *δ*, ppm): 8.59 (1H, *d*, bipy), 8.27 (1H, d, bipy), 8.24–8.19 (2H, m, dppb-HPh), 8.08 (2H, d,L-NO_2_), 8.05–7.98 (2H, m, dppb-HPh), 7.98–7.91 (4H, m, bipy), 7.73–7.65 (3H, m, dppb-HPh), 7.62 (2H, d, L-NO_2_), 7.57 (1H, d, bipy), 7.54–7.48 (3H, m, bipy; dppb-HPh), 7.08 (1H, t, bipy), 7.02 (2H, t,dppb-HPh), 6.99–6.86 (4H, m, dppb-HPh), 6.70–6.64 (2H, m, dppb-HPh), 5.98–5.90 (2H, m, dppb-HPh), 3.29–3.19 (1H, m, dppb-aliphatic), 2.76–2.66 (1H, m, dppb-aliphatic), 2.62–2.53 (2H, m, dppb-aliphatic), 2.36–2.22 (1H, m, dppb-aliphatic), 2.14–1.93 (2H, m, dppb-aliphatic), 1.53–1.53 (1H, m, dppb-aliphatic). ^13^C NMR (100.6 MHz, DMSO-*d*_6_, 298 K, *δ*, ppm): 179.3 (L-NO_2_), 160.2 (bipy), 160.1, 158.6, 155.5(bipy), 149.5 (L-NO_2_), 148.9(bipy), 138.6 (bipy), 128.3 (L-NO_2_), 128.1 (dppb-CPh), 127.8, 127.7, 127.5, 127.4, 127.1 (dppb-CPh), 125.8 (bipy), 123.9, 123.2, 123.0(L-NO_2_), 26.5–21.4 (dppb-aliphatic). ^31^P{^1^H} NMR (162 MHz, dichloromethane (using a capillary containing deuterated water), 298 K) *δ* (ppm), (multiplicity): 49.39 (d) and 45.87 (d), ^2^*J* = 34.0 Hz.

### Stability in solution

Since the experiments are performed with previous solubilization of complexes in DMSO, the stability of the ruthenium complexes was evaluated by using DMSO and RPMI culture medium (10%). The ^31^P{^1^H} NMR spectra of the complexes in this solution (with a deuterated water capillary) were recorded at 0, 24, and 48 h.

### Biological investigation

#### Cell culture

MDA-MB-231 (ATCC No. HTB-26) human triple-negative breast tumor cells, A549 (ATCC No. CCL-185) human lung tumor cells, and MRC-5 (ATCC No. CCL-171) non-tumor human lung cells were cultured in Dulbecco's modified Eagle's medium (DMEM) supplemented with 10% FBS. A2780 (ATCC No. 93112519) human ovarian carcinoma cells, and A2780cis (ECACC No. 93112517) cisplatin-resistant human ovarian carcinoma cells were cultured in RPMI-1640 medium supplemented with 10% FBS. All the cell lines were maintained in an incubator with humidified 5% CO_2_ atmosphere at 37 °C. The MDA-MB-231 and A2780 cell lines were purchased from BCRJ (Banco de Células do Rio de Janiero, Brazil). The remaining cell lines were kindly provided by Dra. Marcia R. Cominetti, from the department of gerontology, UFSCar, São Carlos. Culture medium were purchased from Vitrocell and FBS from Gilbco.

#### *In vitro* cell viability

To conduct the assay, 1.5 × 10^4^ cells per well were seeded in 150 µL of appropriate medium in 96-well plates and incubated in 5% CO_2_ at 37 °C for 24 h to allow cell adhesion. The complexes were dissolved in dimethyl sulfoxide, and 0.75 mL of one of the complex solutions was added to each well (final concentration of 0.5% dimethyl sulfoxide per well). For cisplatin, dimethylformamide (DMF) was used as solvent (0.5%). Untreated cells were the negative control. The cells were incubated with one of the complexes in 5% CO_2_ at 37 °C for 48 h. After this treatment, MTT (50 mL, 1 mg mL^−1^ in PBS buffer) was added to each well, and the plate was incubated for 4 h. Cell viability was detected by MTT reduction to purple formazan in living cells. The formazan crystals were solubilized with DMSO (150 µL per well), and the optical density of each well was measured at 540 nm by using a multiscanner autoreader. The percentage cell viability was calculated by dividing the average absorbance of cells treated with the complexes by that of the control; % cell viability *versus* drug concentration (logarithmic scale) was calculated using Hill's Equation in GraphPad Prism software (version 8.0) to determine the IC_50_ (drug concentration that inhibits 50% of the cells' viability relative to the control), with its estimated error derived from the average of three experiments in triplicate.

#### Morphological changes

A2780 cells (1.0 × 10^5^ cells per well) were seeded in a 12-well plate and incubated in supplemented medium and 5% CO_2_ at 37 °C for 24 h. Cell morphology was examined in an inverted microscope (Nikon, T5100) with a 10× objective at 0, 24, and 48 h after the cells were treated with different concentrations of complex RuNH_2_.

#### Clonogenic assay

A2780 or MRC-5 cells (400 cells per well) were seeded in a six-well plate and incubated in 5% CO_2_ at 37 °C for 24 h for cell adhesion. Then, the cells were treated with different concentrations of complex RuNH_2_ for 48 h. The medium was replaced with fresh medium without any complex, and the cells were incubated for 10 days. Next, the cells were washed with PBS, fixed with 1 : 1 (v/v) methanol and water with 0.5% methyl violet for 10 min, and washed with water. Relative survival was calculated by using the ImageJ software, by applying the plugin “ColonyArea” that measures the area and intensity of each colony in the selected image.^42^

#### DAPI/PI staining

A2780 Cells (1.0 × 10^4^ cells per well) were plated in 96-well plates and allowed to attach for 24 h at 37 °C in a humidified atmosphere containing 5% CO_2_. After adhesion, the cells were exposed to complex 3 at the indicated concentrations and incubated for an additional 48 h. Subsequently, the medium was replaced with 100 µL of propidium iodide (PI, 1 µg mL^−1^) and the cells were incubated for 1 h. DAPI (4′,6-diamidino-2-phenylindole dilactate, 2.6 µg mL^−1^; 100 µL) was then added and incubation continued for another 1 h. Fluorescence images were acquired using a CELENA® S Digital Imaging System (Logos Biosystems).

#### Cell cycle analysis

A2780 cells were seeded in 12-well plates at a density of 1 × 10^5^ cells per well and incubated for 24 h at 37 °C in a humidified atmosphere containing 5% CO_2_. After this period, the cells were exposed to different concentrations of RuNH_2_ for 48 h. Following treatment, the cells were harvested, washed with ice-cold PBS, and fixed in 70% ethanol at −20 °C for 24 h. After fixation, samples were centrifuged at 2000 rpm for 5 min at 4 °C and resuspended in 150 µL of PBS containing RNase A (0.2 mg mL^−1^) and propidium iodide (PI, 5 µg mL^−1^) in hypotonic fluorochrome solution. The suspensions were incubated for 30 min prior to analysis. Cell cycle distribution was evaluated using an Accuri C6 flow cytometer (BD Biosciences), acquiring 10 000 events per sample. Data were processed using FlowJo software, and experiments were performed in triplicate. Untreated cells served as the negative control.

## HSA binding experiment

Studies of the interaction between HSA (from Sigma-Aldrich) and the complexes were performed by a fluorescence quenching experiment, where the concentration of HSA in buffer (4.5 mM Tris-HCl, 0.5 mM NaOH, and 50 mM NaCl) at pH 7.4 was kept constant (2.5 µM), while the concentration of the complexes was increased from 2.5 to 17.5 µM. Extinction of the emission intensity of the HSA tryptophan residues at 305 nm (excitation wavelength 270 nm) was monitored at 25, 30 and 37 °C. Data were analyzed by using the classic Stern–Volmer equation (eqn (1)).1
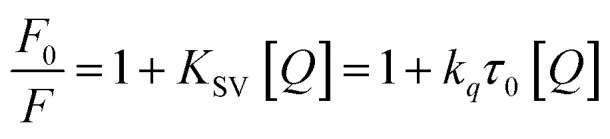
where *F*_0_ and *F* correspond to the fluorescence intensities in the absence and presence of the quencher, respectively; [*Q*] is the concentration of the quencher; and *K*_sv_ is the Stern–Volmer quenching constant. The binding constant (*K*_b_) as well as the number of binding sites (*n*) was determined by plotting the double log graph of the fluorescence data by using eqn (2).2
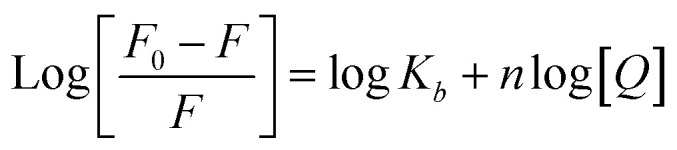


The thermodynamic parameters Δ*H*, Δ*S*, and Δ*G* were obtained by using eqn (3) and (4)3
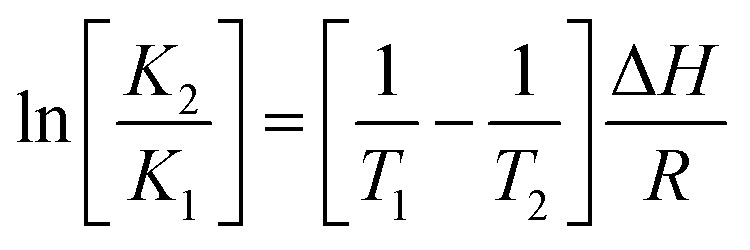

4 Δ*G* = −RT ln *K*_b_ = Δ*H* – TΔ*S*

where *K*_1_ and *K*_2_ are the binding constants at temperatures *T*_1_ and *T*_2_, respectively; and *R* is the gas constant.

## Author contributions

conceptualization: João Honorato de Araujo-Neto, Alzir A. Batista; methodology: Jocely L. Dutra, Gustavo Moselli, Pedro H. S. Marcon, Carlos André F. Moraes, Fabiano M. Niquini, Javier Ellena; investigation*:* Jocely L. Dutra, Gustavo Moselli, Pedro H. S. Marcon, Carlos André F. Moraes, João Victor F. da Costa; crystallography (X-ray data collection and refinement): Pedro H. S. Marcon, Fabiano M. Niquini, Javier Ellena, João Honorato de Araujo-Neto; data curation and validation: Jocely L. Dutra, Gustavo Moselli, Carlos André F. Moraes; formal analysis*:* João Honorato de Araujo-Neto, João Victor F. da Costa; writing – original draft*:* Jocely L. Dutra, Pedro H. S. Marcon, João Honorato de Araujo-Neto; writing – review & editing*:* Javier Ellena, Alzir A. Batista, Ataualpa A. C. Braga; supervision: João Honorato de Araujo-Neto, Ataualpa A. C. Braga, Alzir A. Batista; funding acquisition: João Honorato de Araujo-Neto, Javier Ellena, Ataualpa A. C. Braga, Alzir A. Batista.

## Conflicts of interest

There are no conflicts to declare.

## Note added after first publication

This article replaces the version published on 16 April, which included a smaller version of Fig. 4 that was difficult to read. The changes are to the layout only, with the scientific content unaffected.

## Supplementary Material

RA-016-D5RA07271A-s001

RA-016-D5RA07271A-s002

## Data Availability

CCDC 2487110 (RuNH_2_), 2487109 (RuCBr), 2487111 (RuBi) and 2513690 (RuNO_2_) contain the supplementary crystallographic data for this paper.^62*a*–*d*^ Supplementary information (SI): full characterization of the ruthenium(ii) complexes, including ESI-MS, FTIR, and multinuclear NMR data (^1^H, ^13^C, and ^31^P), as well as electrochemical (cyclic voltammetry) and computational studies. It also contains crystallographic data, stability studies in biological media, and biological evaluation results, including cytotoxicity assays and HSA interaction studies. See DOI: https://doi.org/10.1039/d5ra07271a.
