# Supplementary material for: Para-substituted benzoic acid ruthenium(ii) complexes: structural features modulating cytotoxicity
Source: RSC Adv. 2026 Apr 16;16(22):19763–78. doi: 10.1039/d5ra07271a (PMC13085785; doi:10.1039/d5ra07271a)
Supplement: RA-016-D5RA07271A-s001 [file RA-016-D5RA07271A-s001.pdf]

Supplementary Information (SI) for RSC Advances.
This journal is © The Royal Society of Chemistry 2026

ELETRONIC SUPPORTING INFORMATION (ESI)

Para-substituted benzoic acid ruthenium(II) complexes: structural features modulating cytotoxicity

Jocely L. Dutra[a], Pedro H. S. Marcon[b], Gustavo Moselli[a], Fabiano M. Niquini[a], João Victor F. da Costa[a], Carlos André F. Moraes[a], Ataualpa A. C. Braga[a], Javier Ellena[c], Alzir A. Batista[a] and João Honorato de Araujo-Neto[b]*

[a] *Departament of Chemistry, Universidade Federal de São Carlos (UFSCar), 13561-905, São Carlos, SP, Brazil.*
[b] *Department of Fundamental Chemistry, Institute of Chemistry, University of São Paulo. 05508-000 São Paulo, SP, Brazil.*
[c] *Instituto de Física de São Carlos, Universidade de São Paulo (USP), CP 369, CEP 13560-970, São Carlos, SP, Brazil.*

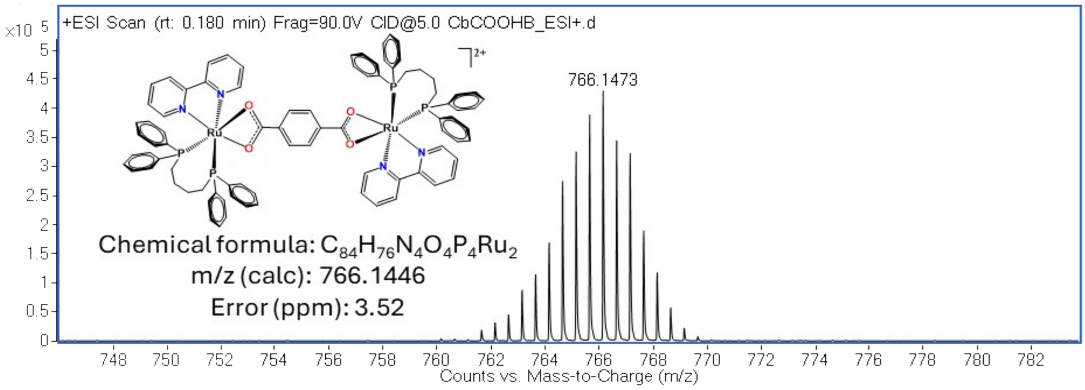

Figure S1. ESI(+)-MS analysis of the complex **RuBi**.

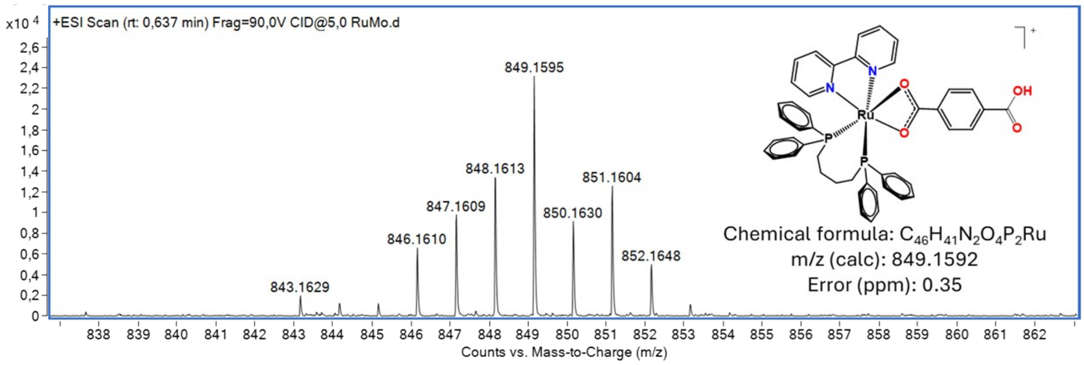

Figure S2. ESI(+)-MS analysis of the complex **RuMono.**

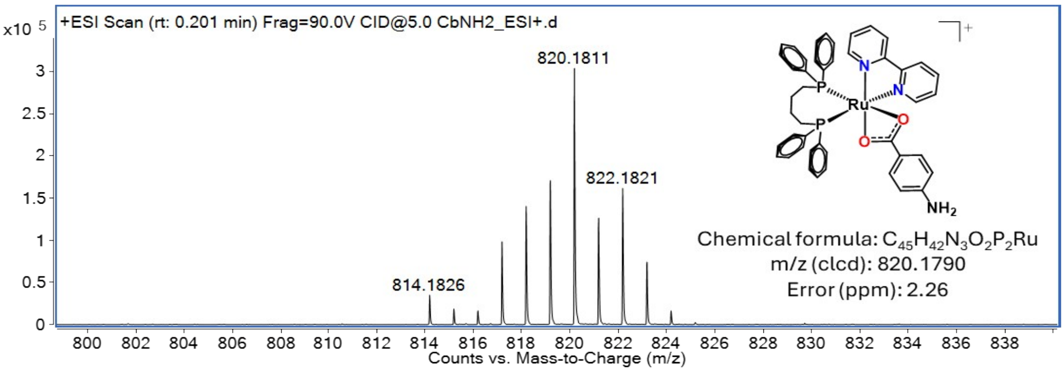

Figure S3. ESI(+)-MS analysis of the complex **$RuNH_2$**.

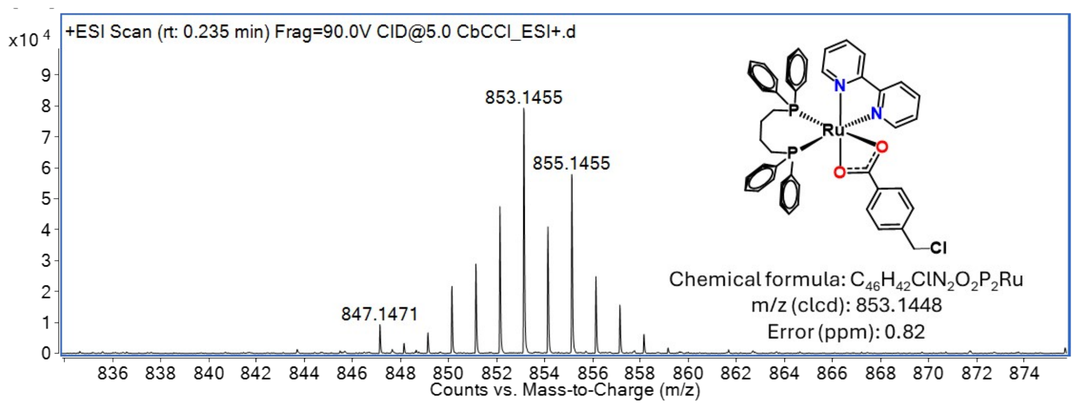

Figure S4. ESI(+)-MS analysis of the complex **RuCCl**.

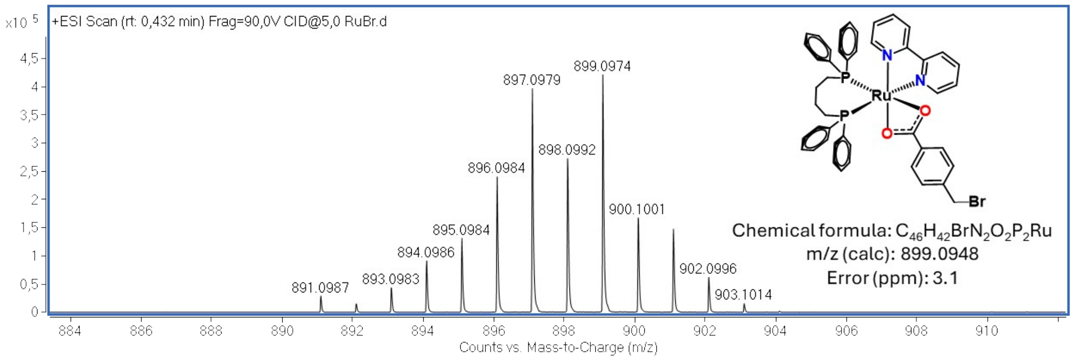

Figure S5. ESI(+)-MS analysis of the complex **RuCBr**.

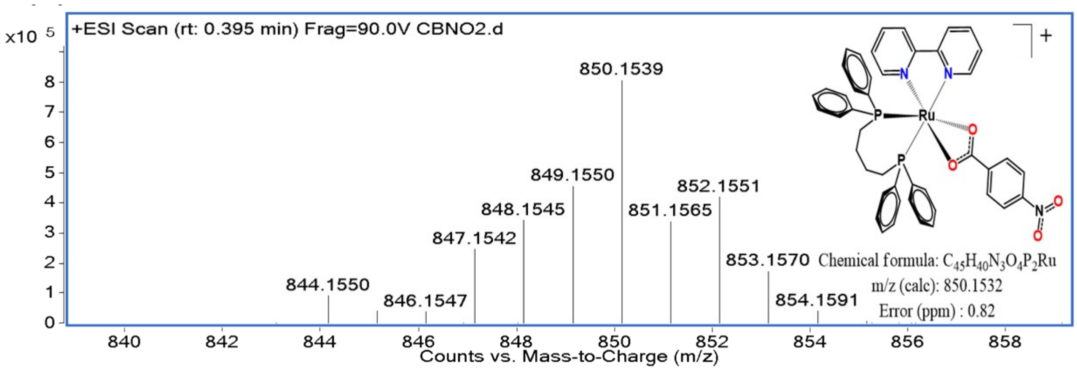

Figure S6. ESI(+)-MS analysis of the complex **RuNO$_2$**

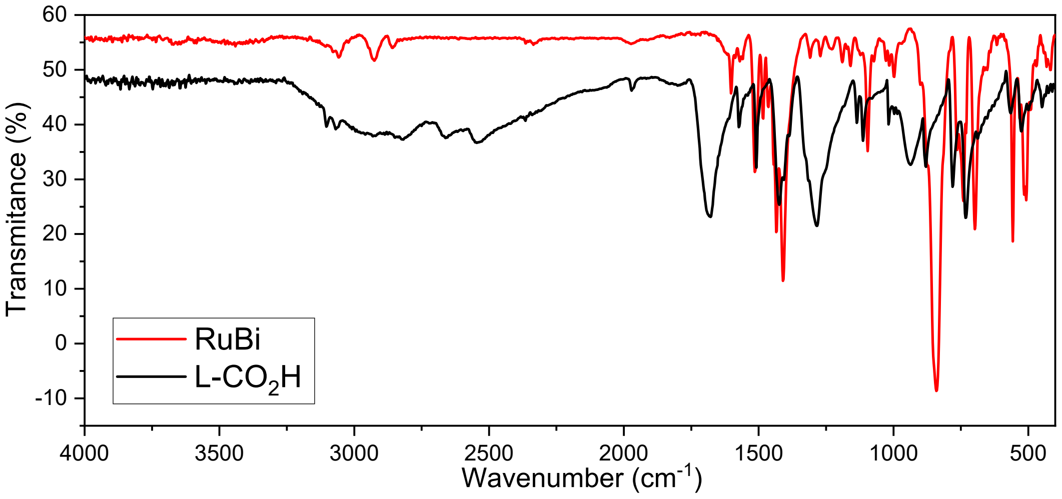

Figure S7. FTIR spectra for **RuBi** and L-CO_2H.

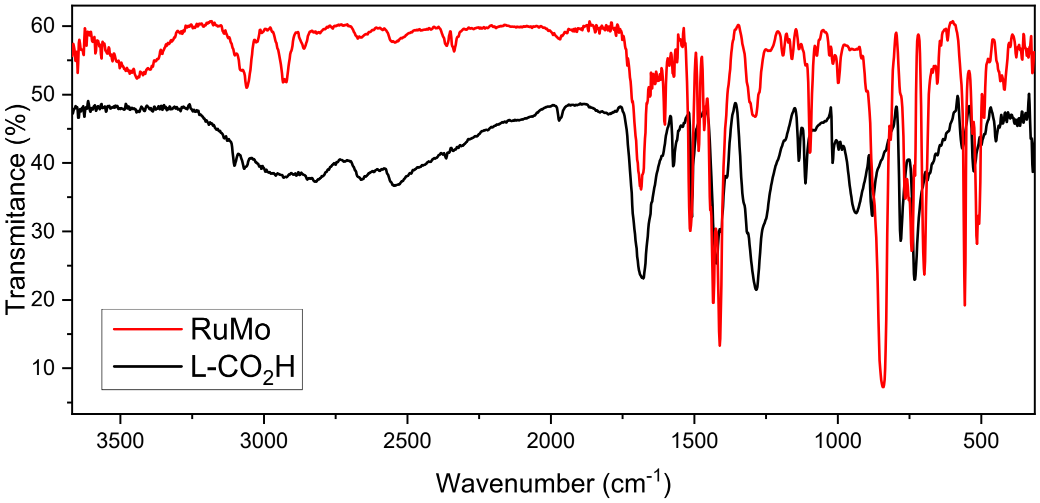

Figure S8. FTIR spectra for **RuMo** and L-CO_2H.

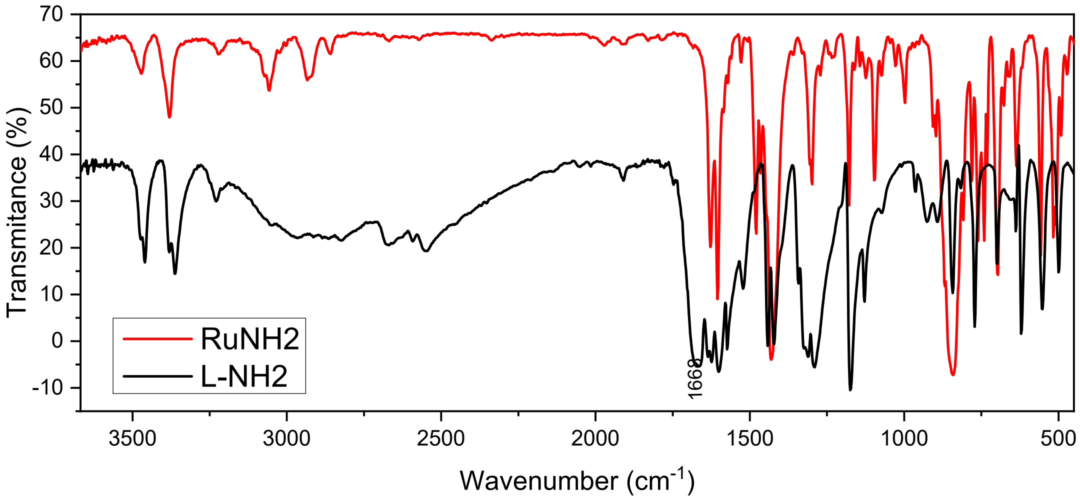

Figure S9. FTIR spectra for **RuNH$_2$** and L-NH_2.

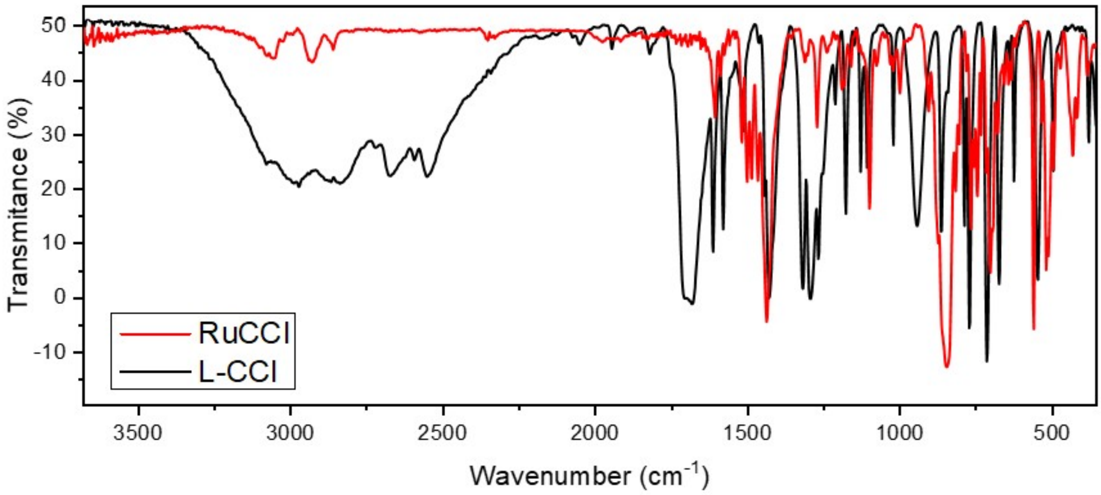

Figure S10. FTIR spectra for **RuCCl** and L-CCl.

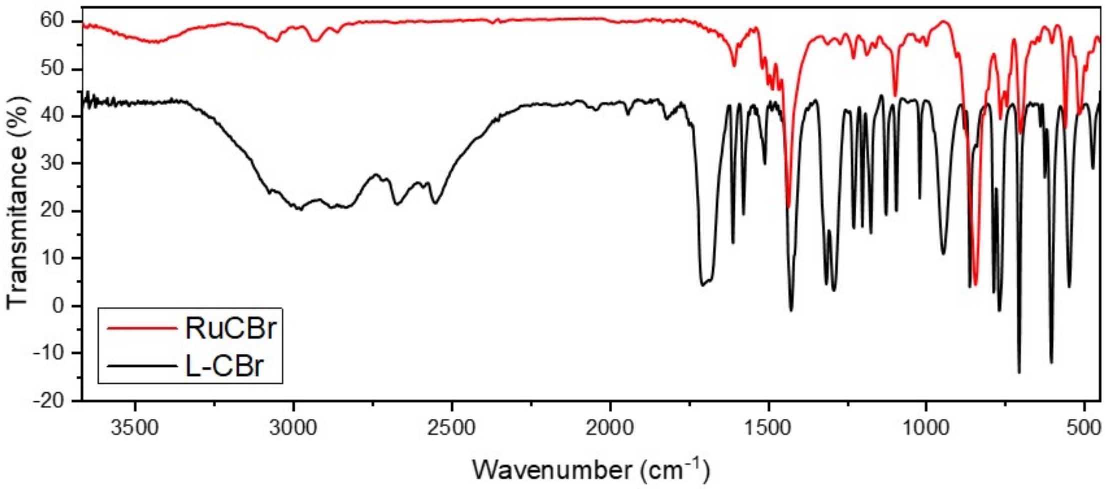

Figure S11. FTIR spectra for **RuCBr** and L-CBr.

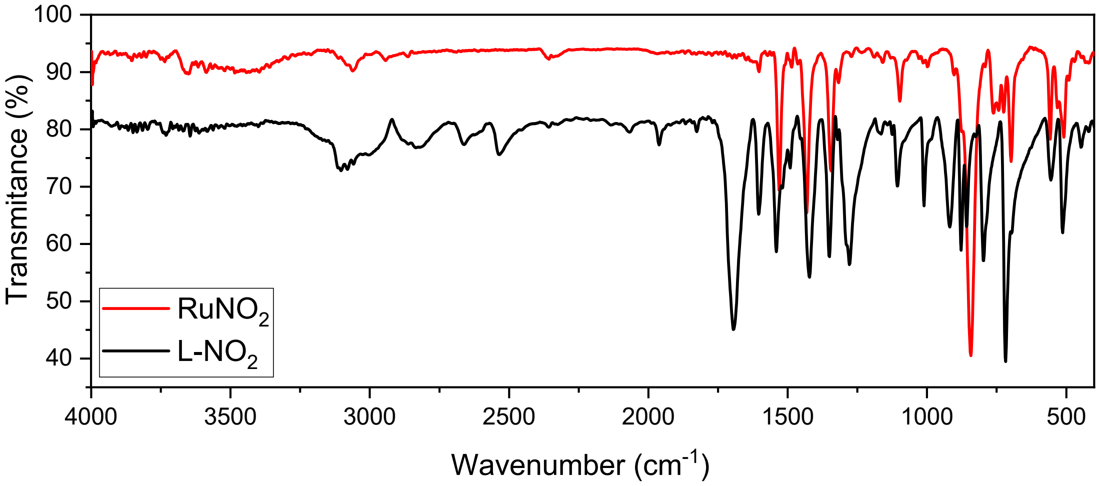

Figure S12. FTIR spectra for **$RuNO_2$** and L-NO_2.

Table S1. Wavenumber values (cm^{-1}) for the asymmetric and symmetric stretching vibrations of the carboxylic group, as well as Δv for the respective free ligands and their complexes.

	v_{asym}(COO)	v_{sym}(COO)	Δv
**L-CO_2H**	1572	1330	242
**L-NH_2**	1571	1341	230
**L-CCl**	1577	1316	261
**L-CBr**	1572	1315	257
**L-NO_2**	1608	1313	295
**RuBi**	1483	1407	76
**RuMo**	1483	1407	76
**RuNH_2**	1479	1428	51
**RuCl**	1485	1436	49
**RuBr**	1483	1429	54
**RuNO_2**	1531	1433	98

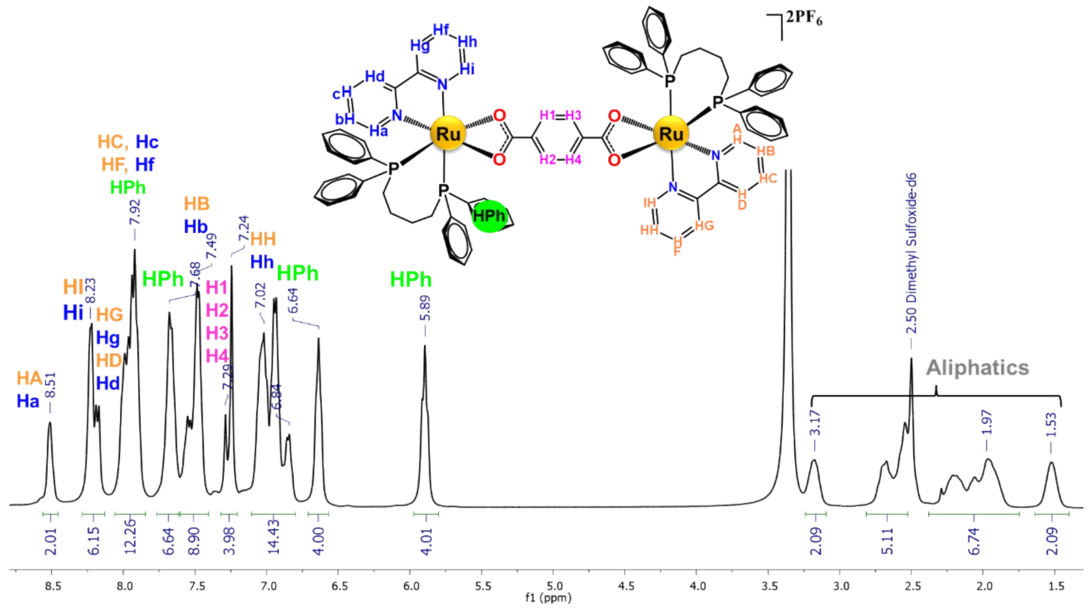

Figure S13. NMR ^{1}H spectra and structure of the complex **RuBi**, in DMSO-d_6.

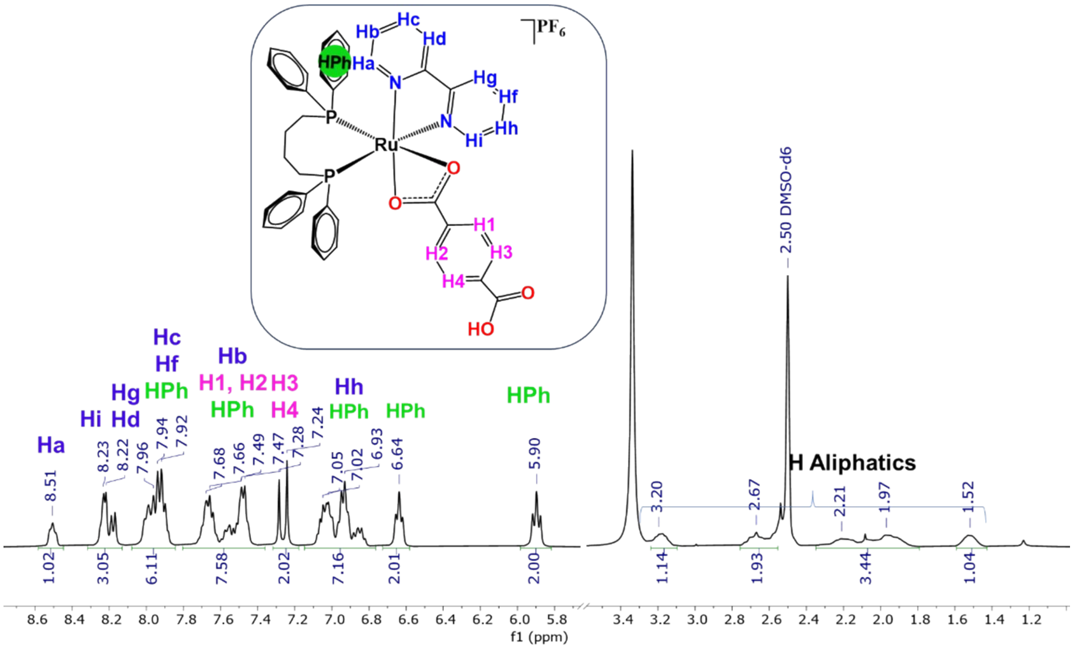

Figure S14. NMR ^{1}H spectra and structure of the complex **RuMo**, in DMSO-d_6.

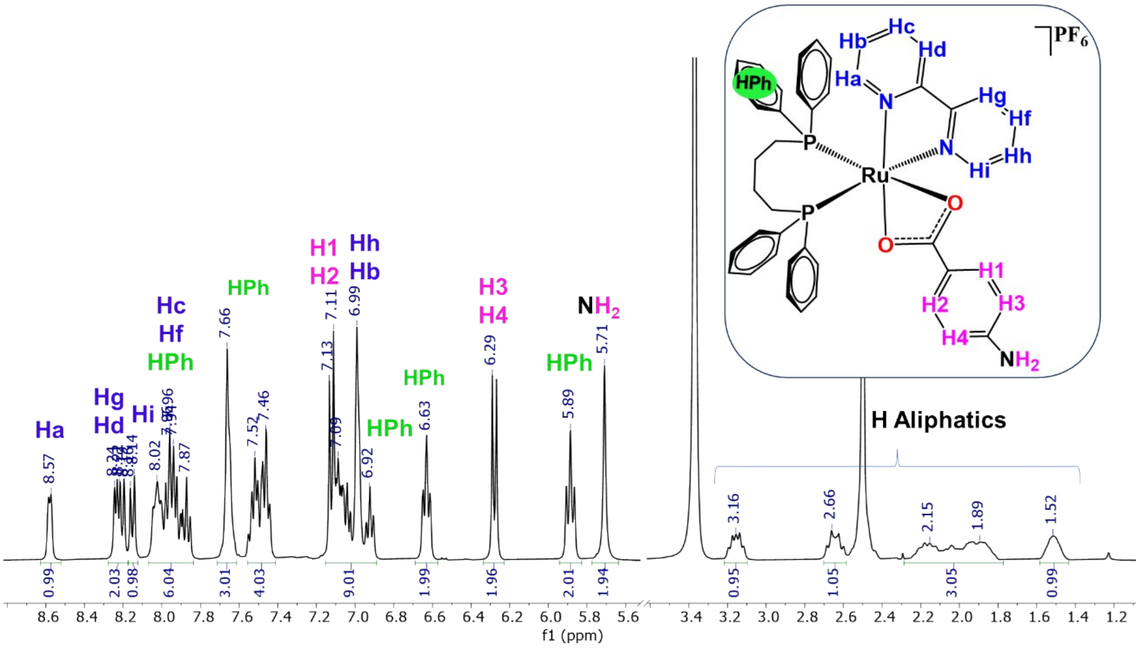

Figure S15. NMR ^{1}H spectra and structure of the complex **RuNH$_2$**, in DMSO-d$_6$.

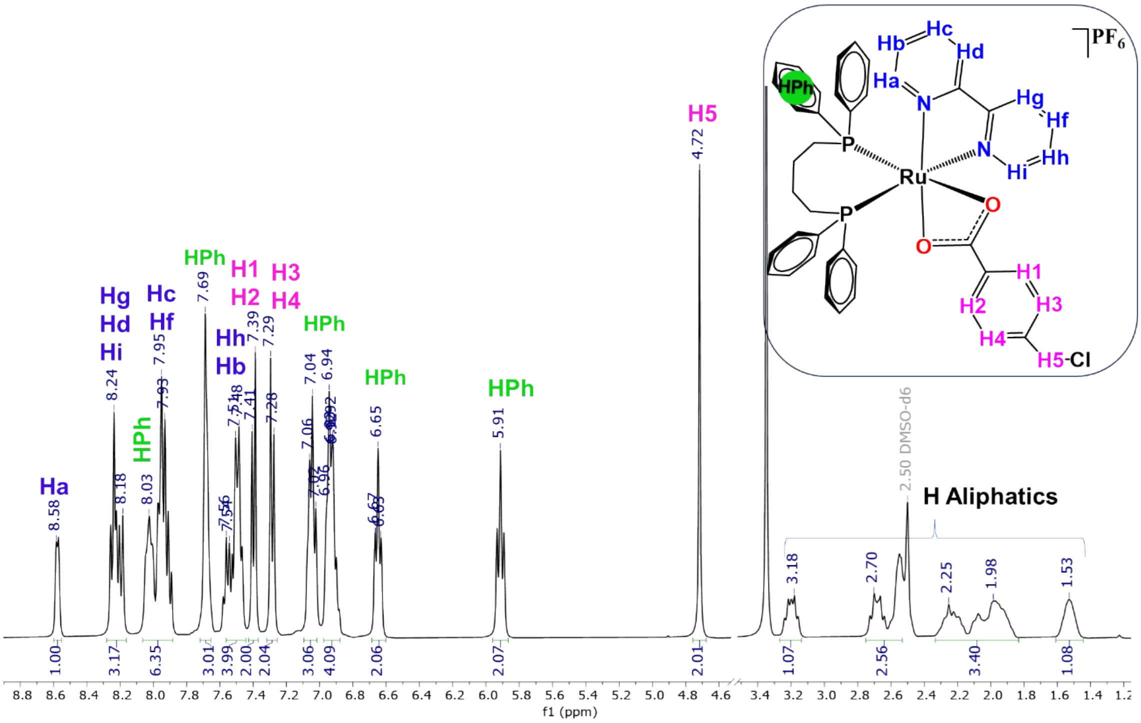

Figure S16. NMR ^{1}H spectra of the complex **RuCCl**, in DMSO-d$_6$.

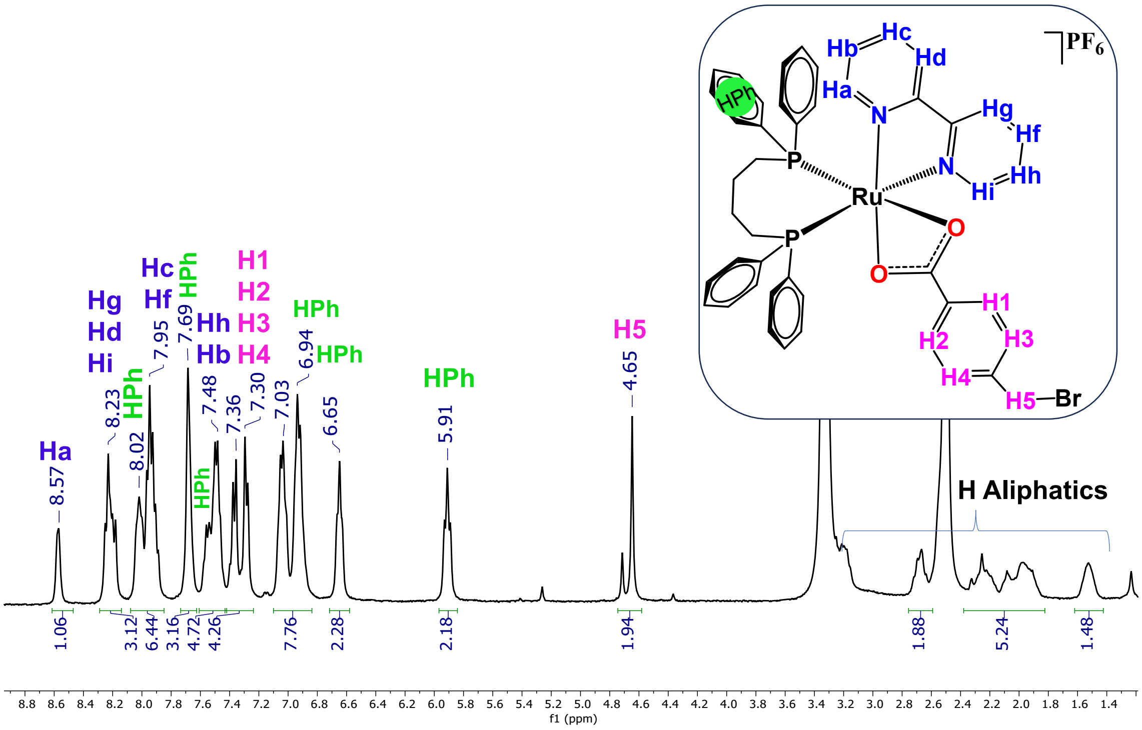

Figure S17. NMR ^{1}H spectra of the complex **RuCBr**, in DMSO-d_6.

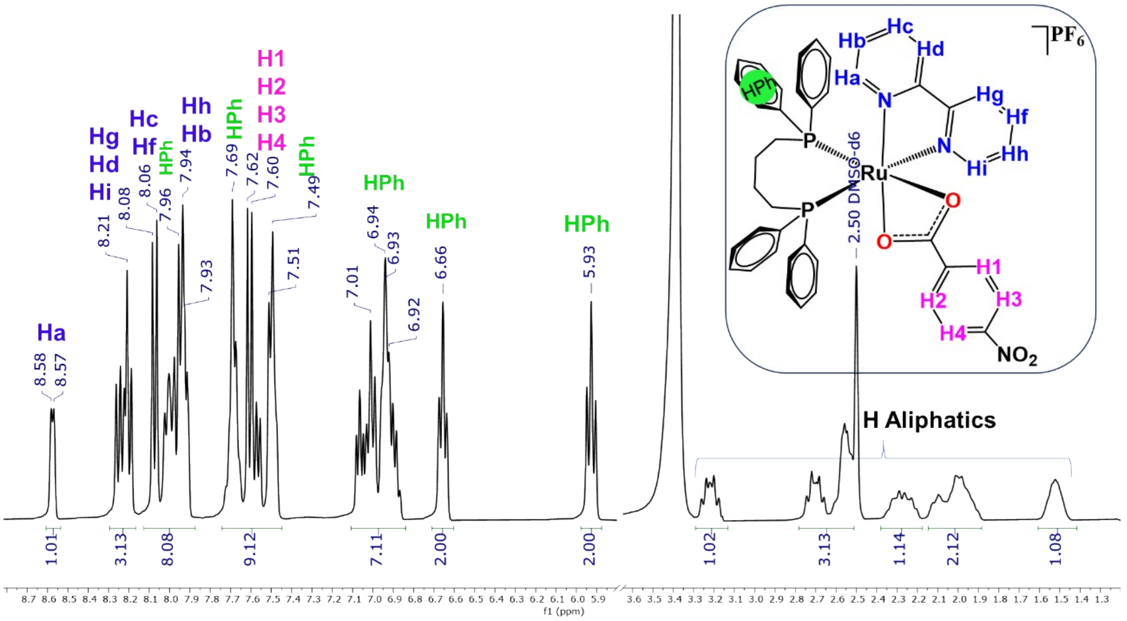

Figure S18. NMR ^{1}H spectra of the complex **RuNO₂**, in DMSO-d_6.

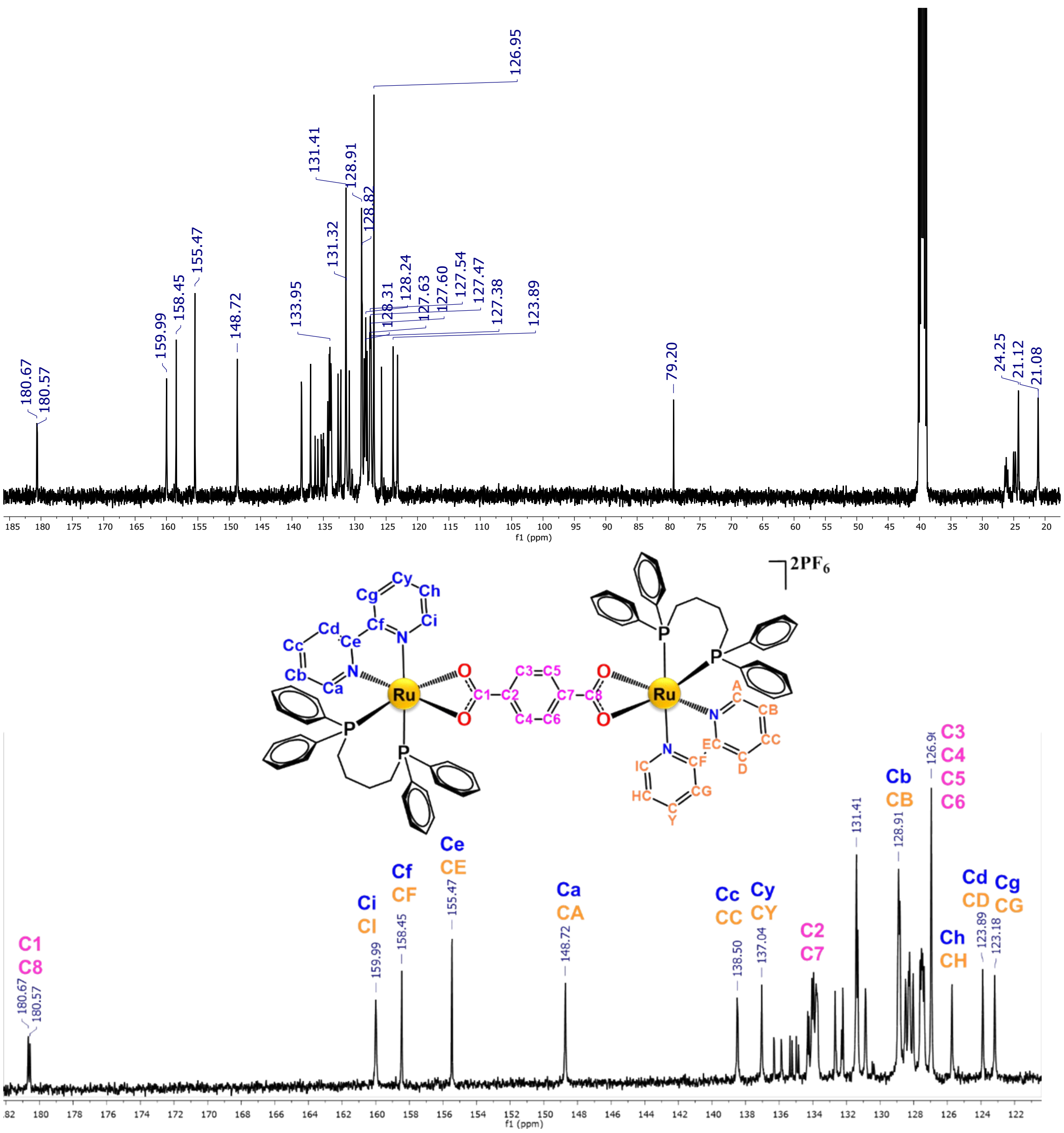

Figure S19. NMR ^{13}C spectra and structure of complex **RuBi**, in DMSO-d_6.

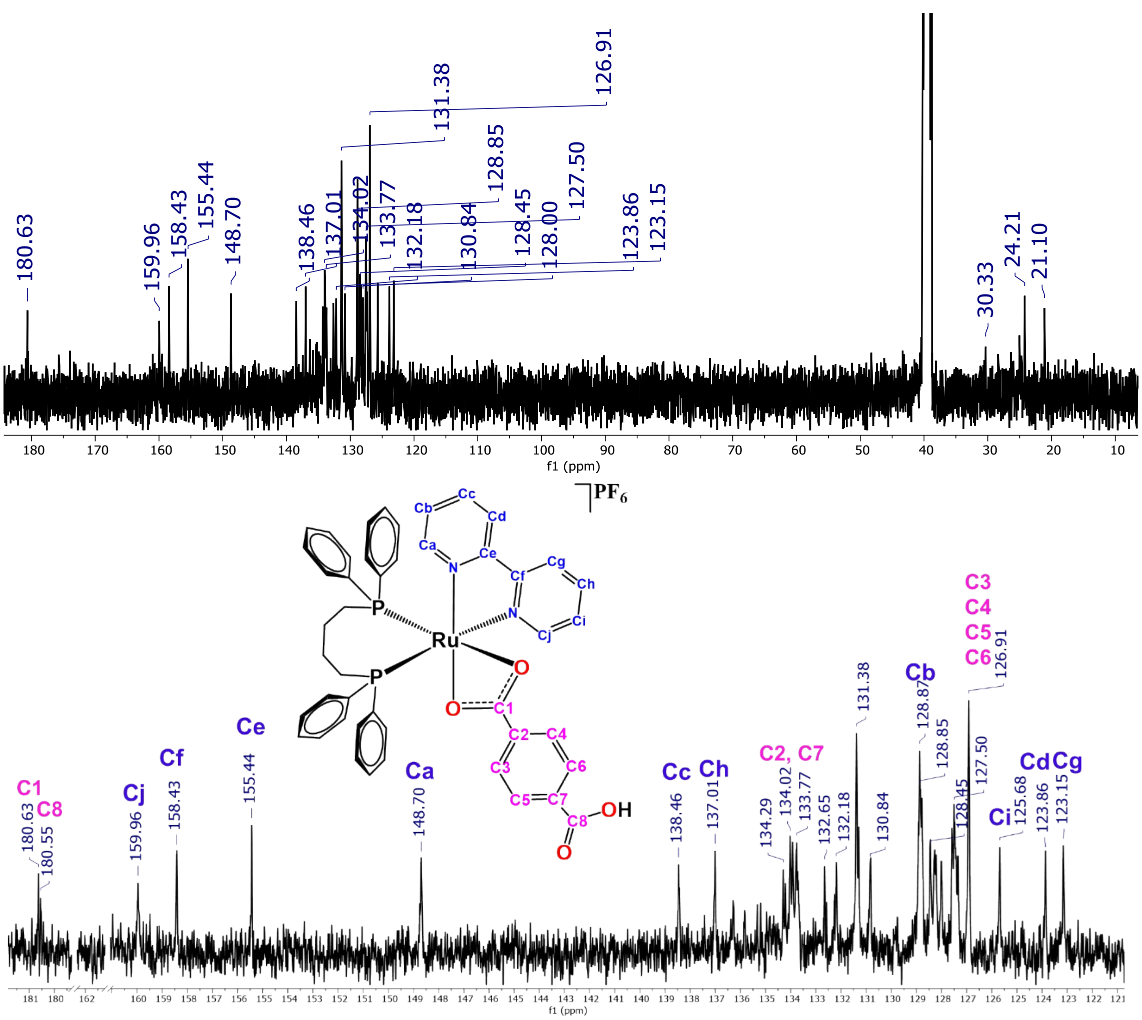

Figure S20. NMR ^{13}C spectra and structure of complex **RuMo**, in DMSO-d$_6$.

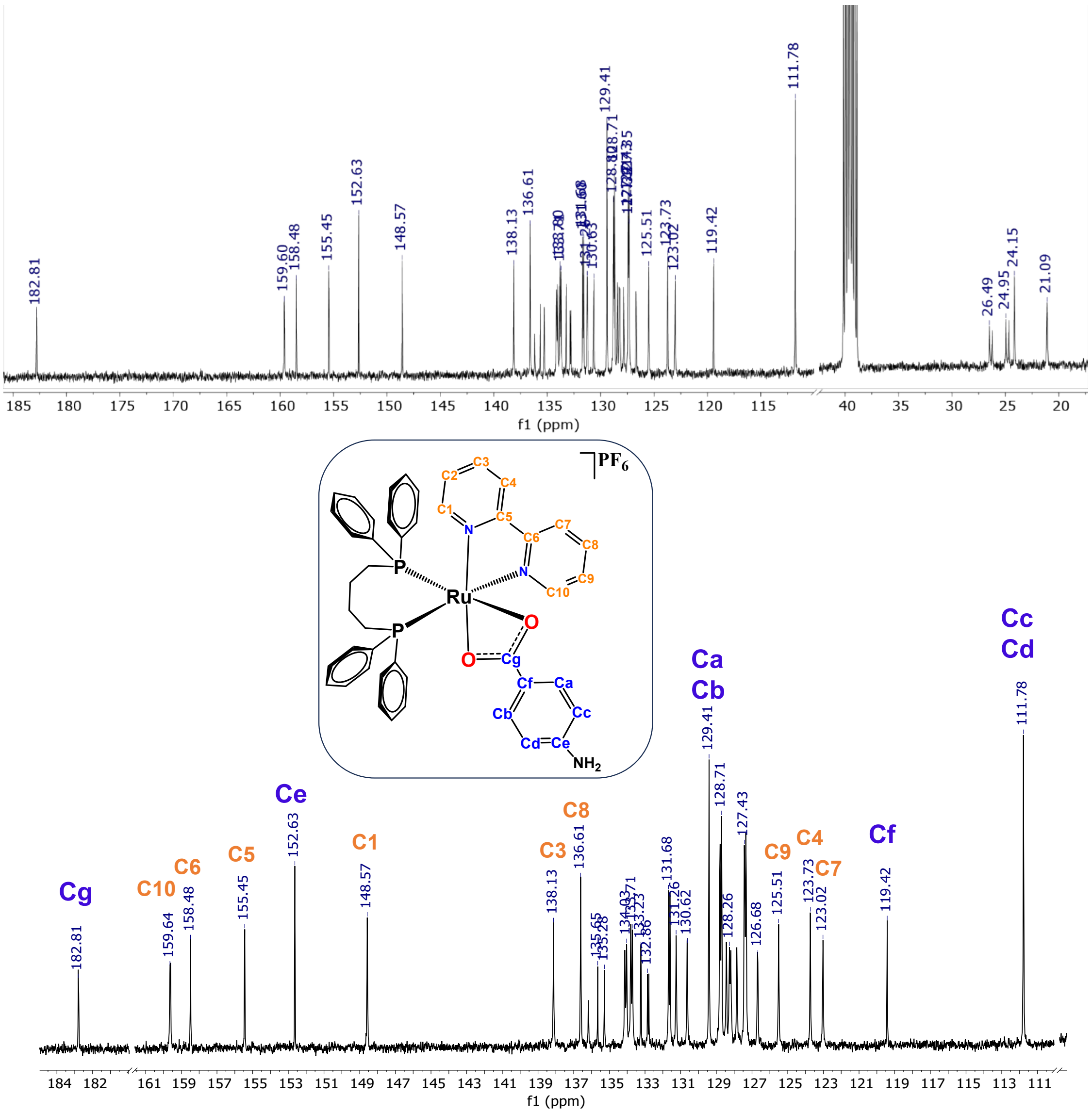

Figure S21. NMR ^{13}C spectra and structure of complex **$RuNH_2$**, in DMSO-d_6.

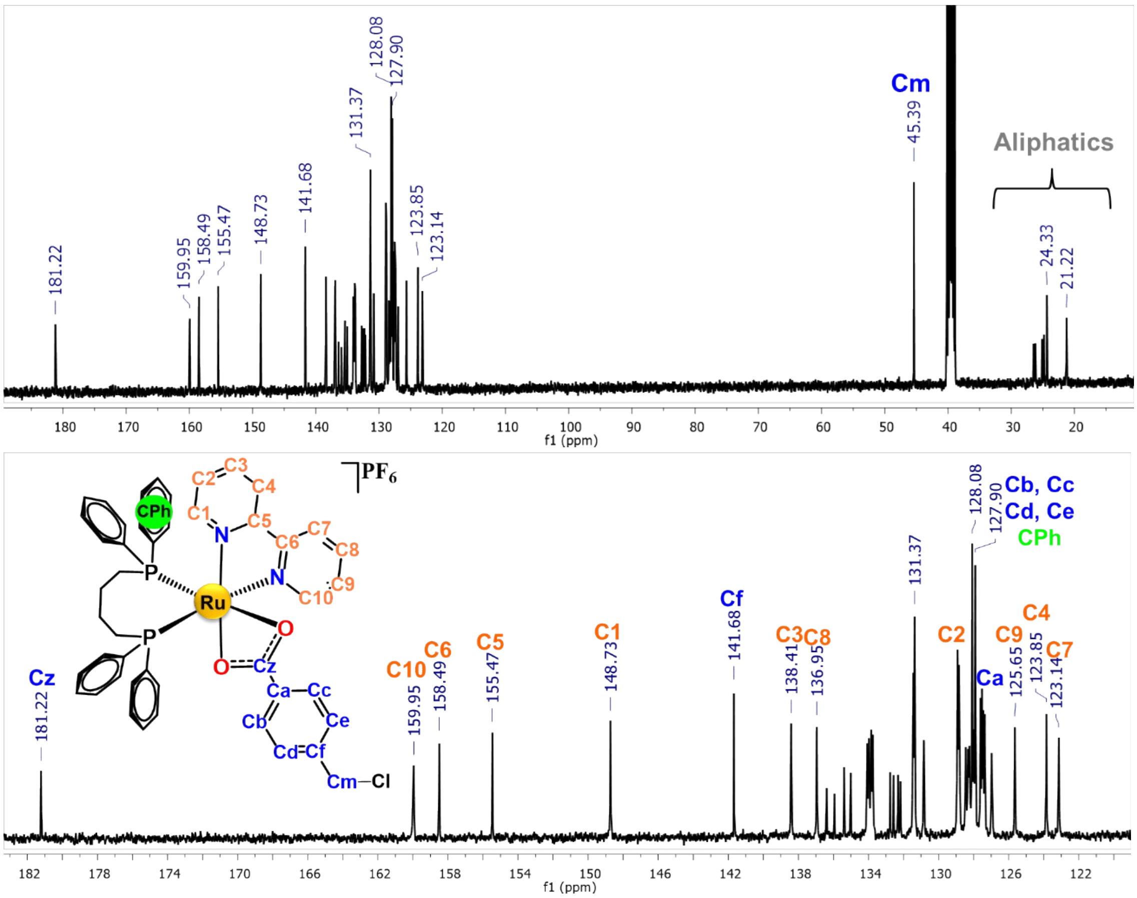

Figure S22. NMR ^{13}C spectra and structure of complex **RuCCl**, in DMSO-d$_6$.

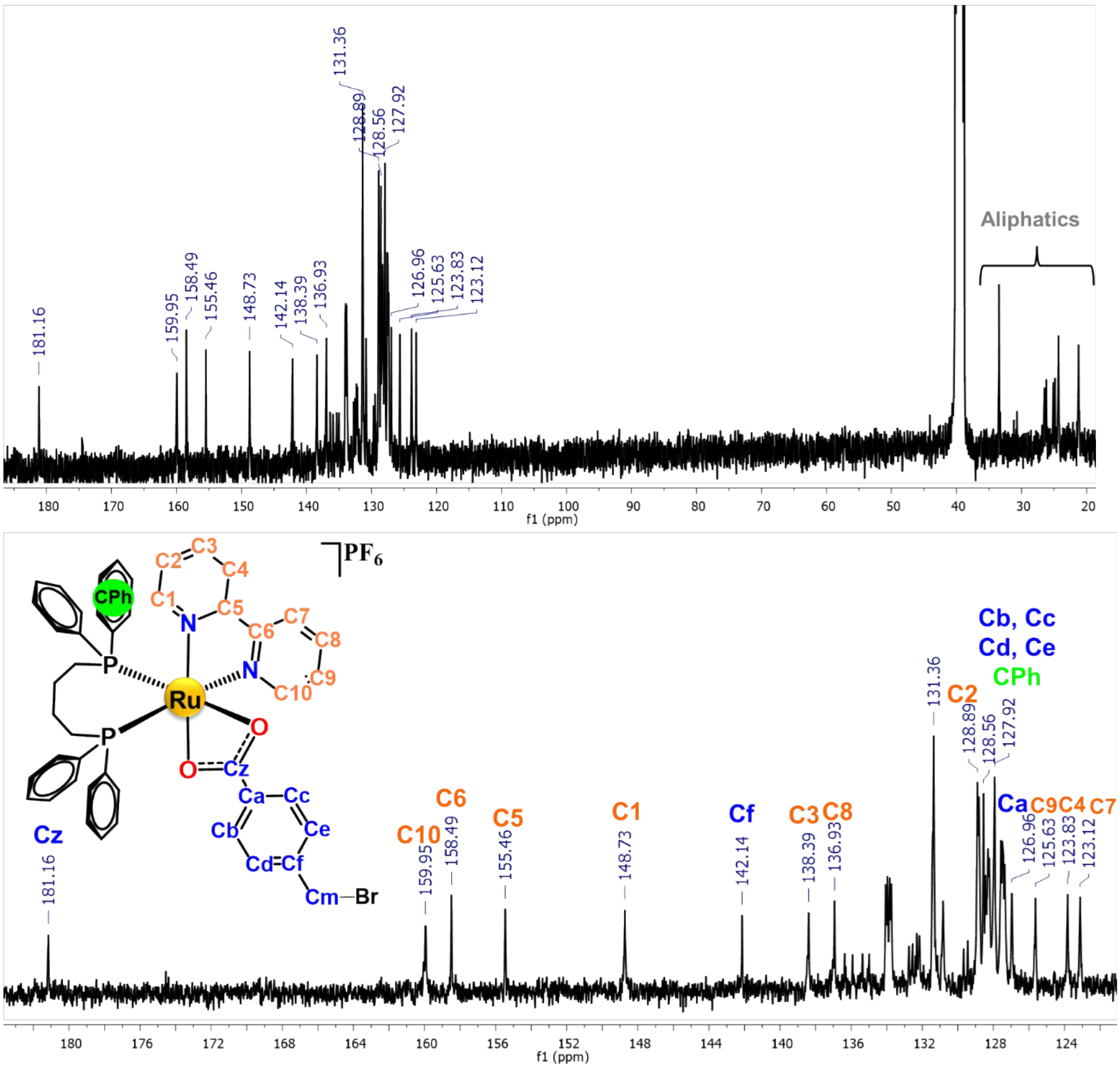

Figure S 23. NMR ^{13}C spectra and structure of complex **RuCBr**, in DMSO-d_6.

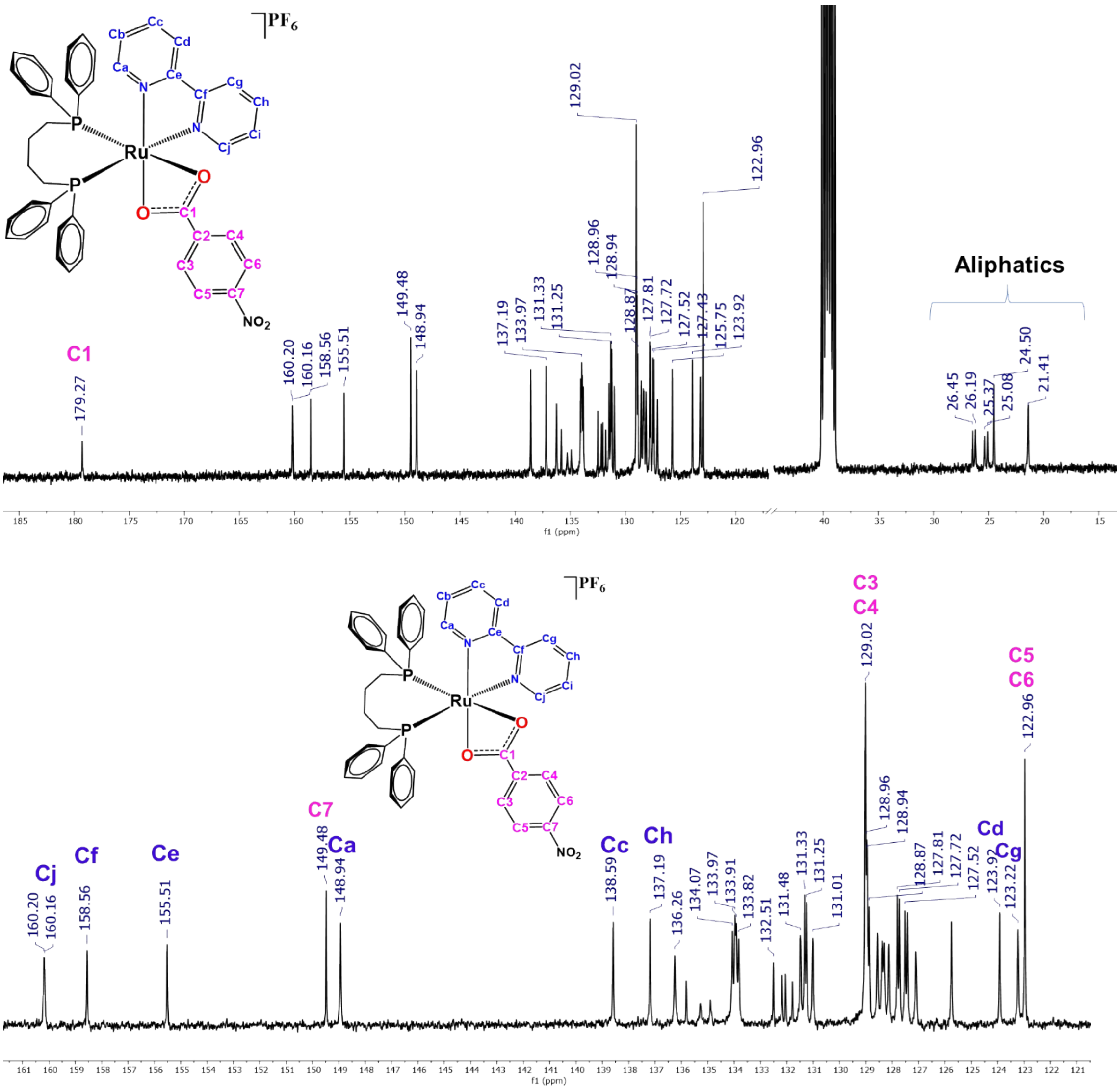

Figure S 24. NMR ^{13}C spectra and structure of complex **$RuNO_2$**, in DMSO-d$_6$.

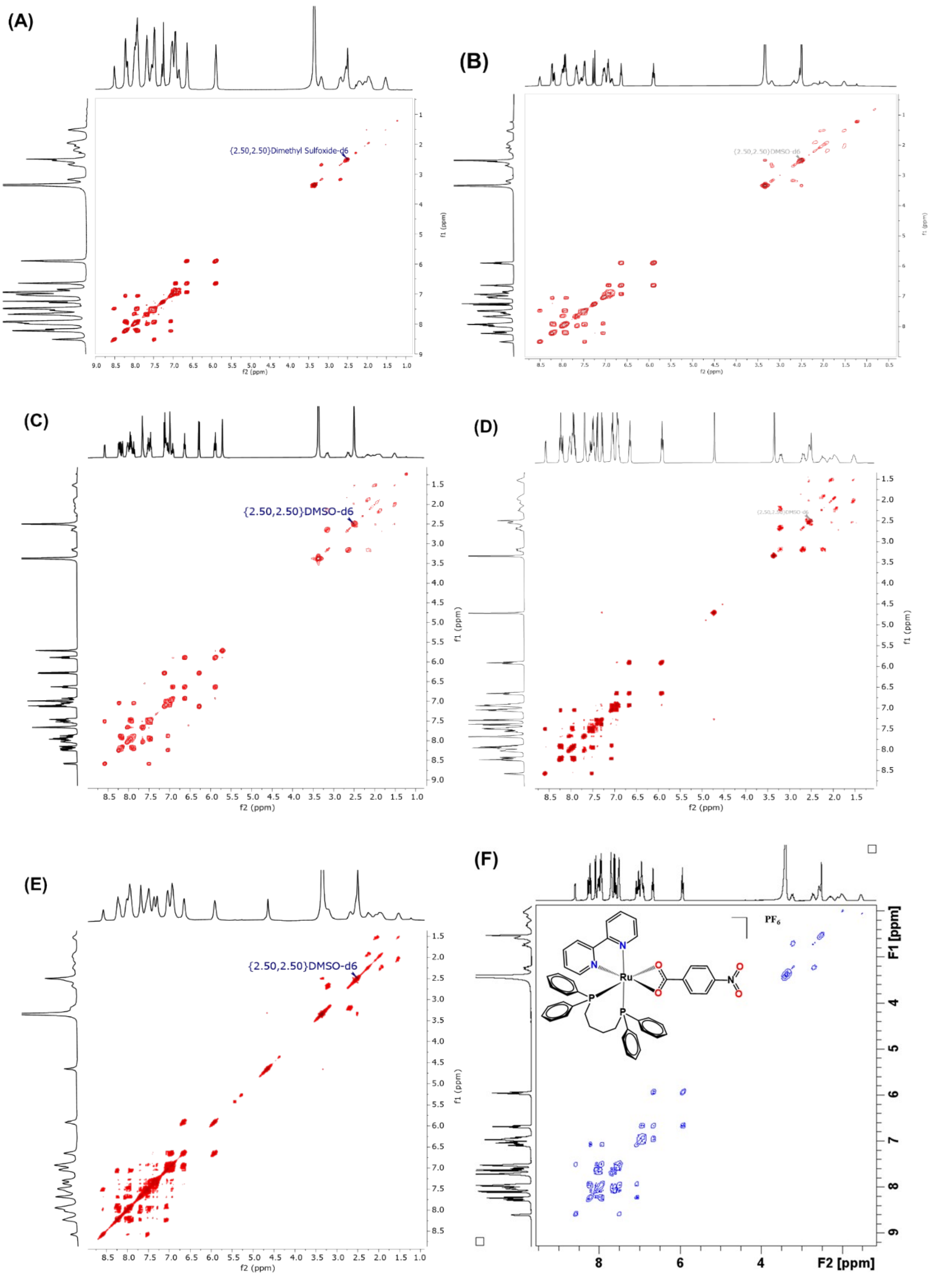

Figure S25. COSY ^1H-^1H NMR spectra of complexes (A) **RuBi**, (B) **RuMo**, (C) **$RuNH_2$**, (D) **RuCCl** and (E) **RuCBr**, in DMSO-d_6.

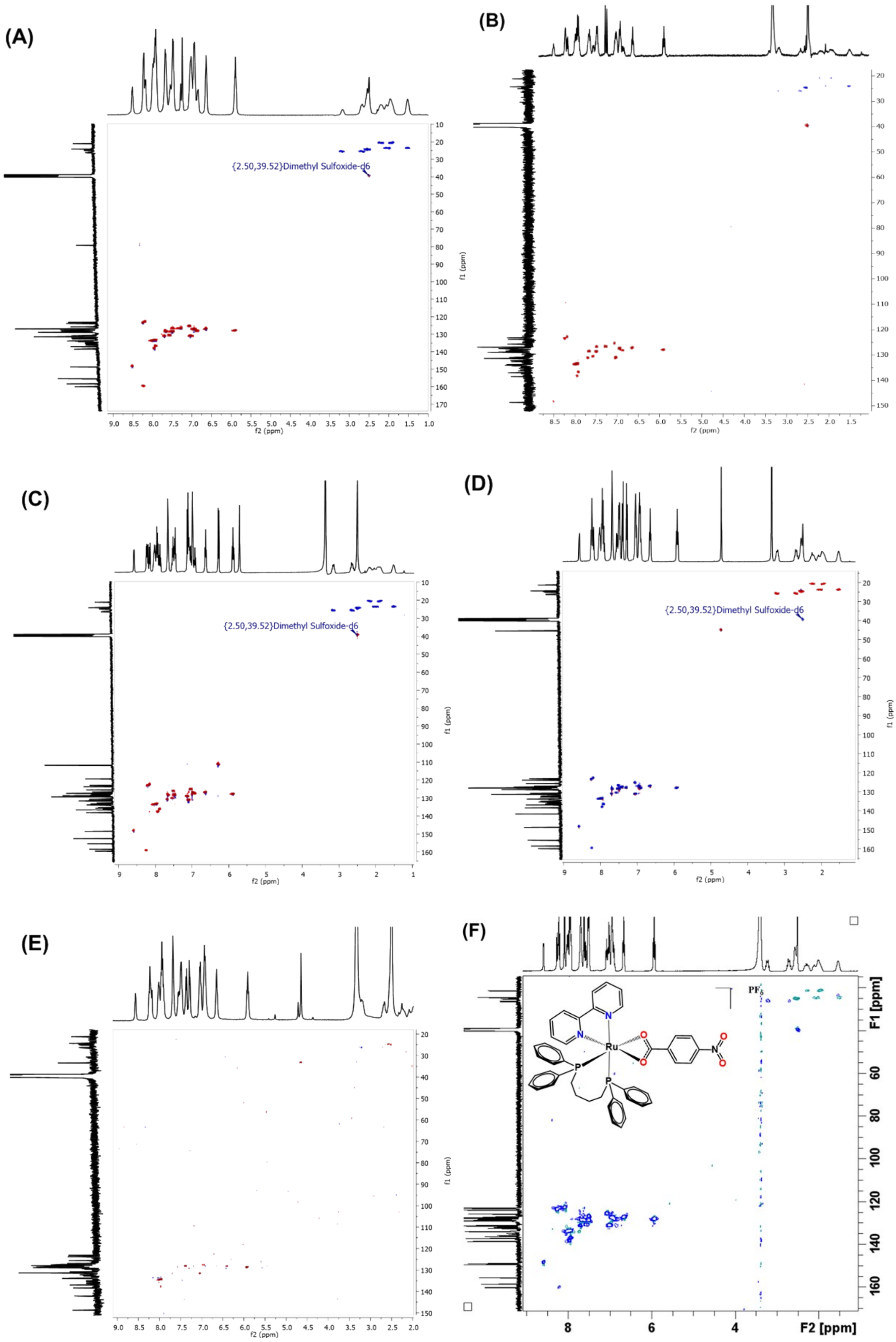

Figure S26. ^{1}H-^{13}C HSQC NMR of complexes (A) **RuBi**; (B) **RuMo**; (C) **$RuNH_2$;** (D) **RuCCl** and (E) **RuCBr**, in DMSO-d_6.

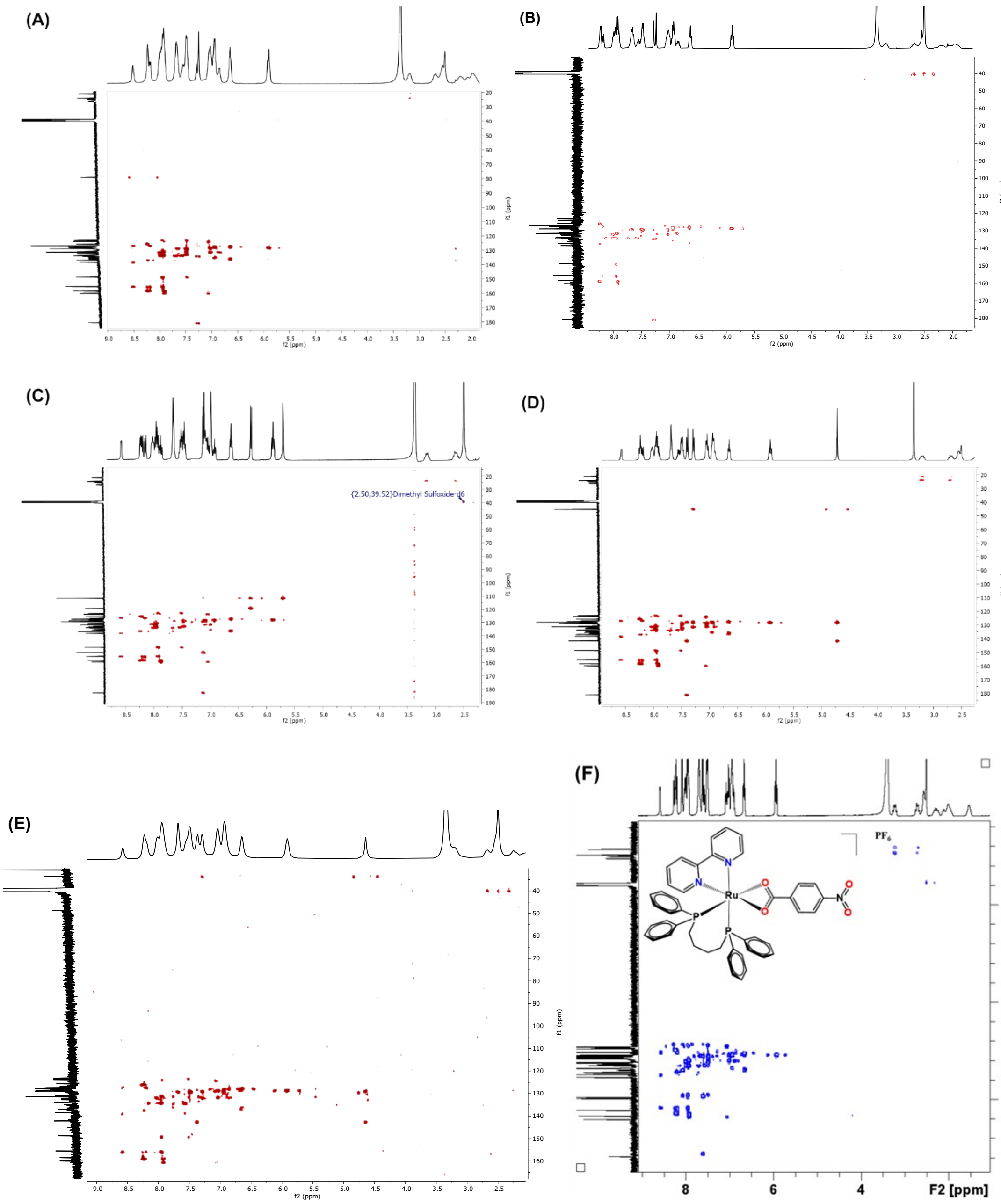

Figure S27. ^{1}H-^{13}C HMBC NMR of complexes (A) **RuBi**, (B) **RuMo**, (C) **$RuNH_2$**, (D) **RuCCl** and (E) **RuCBr**, in DMSO-d_6.

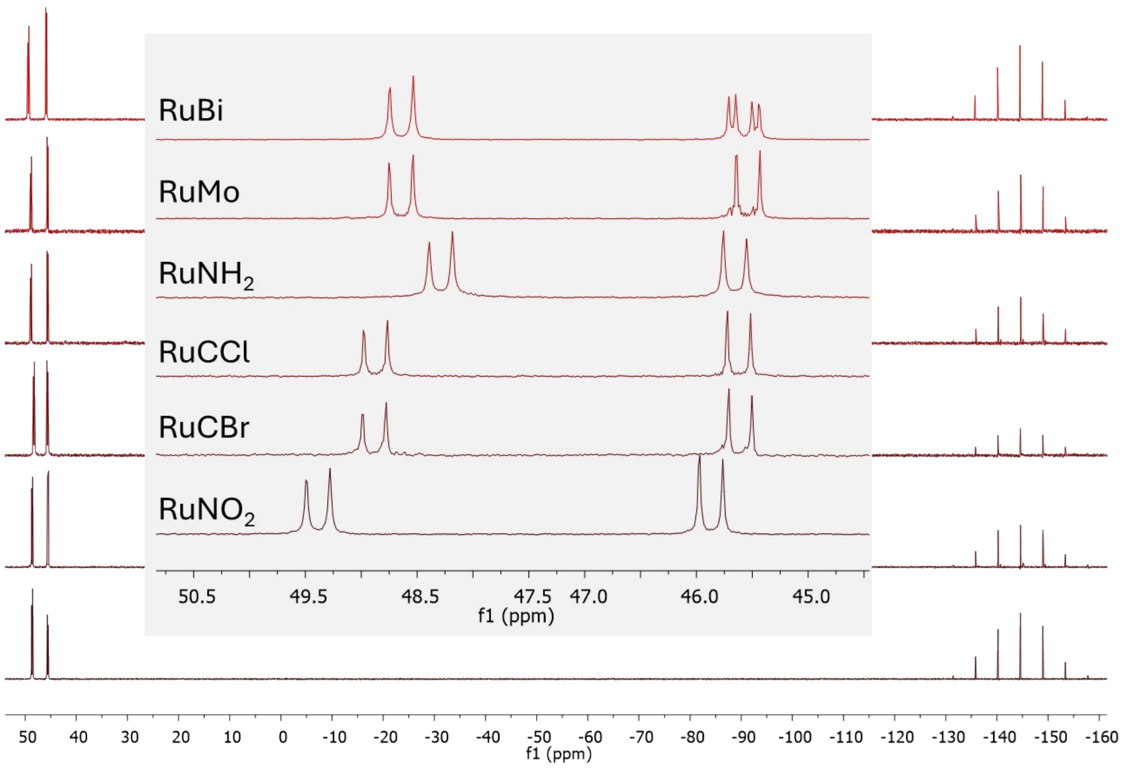

Figure S28. $^{31}P\{^1H\}$ NMR spectra of **Ru** complexes, in CH_2Cl_2 (with D_2O capillary).

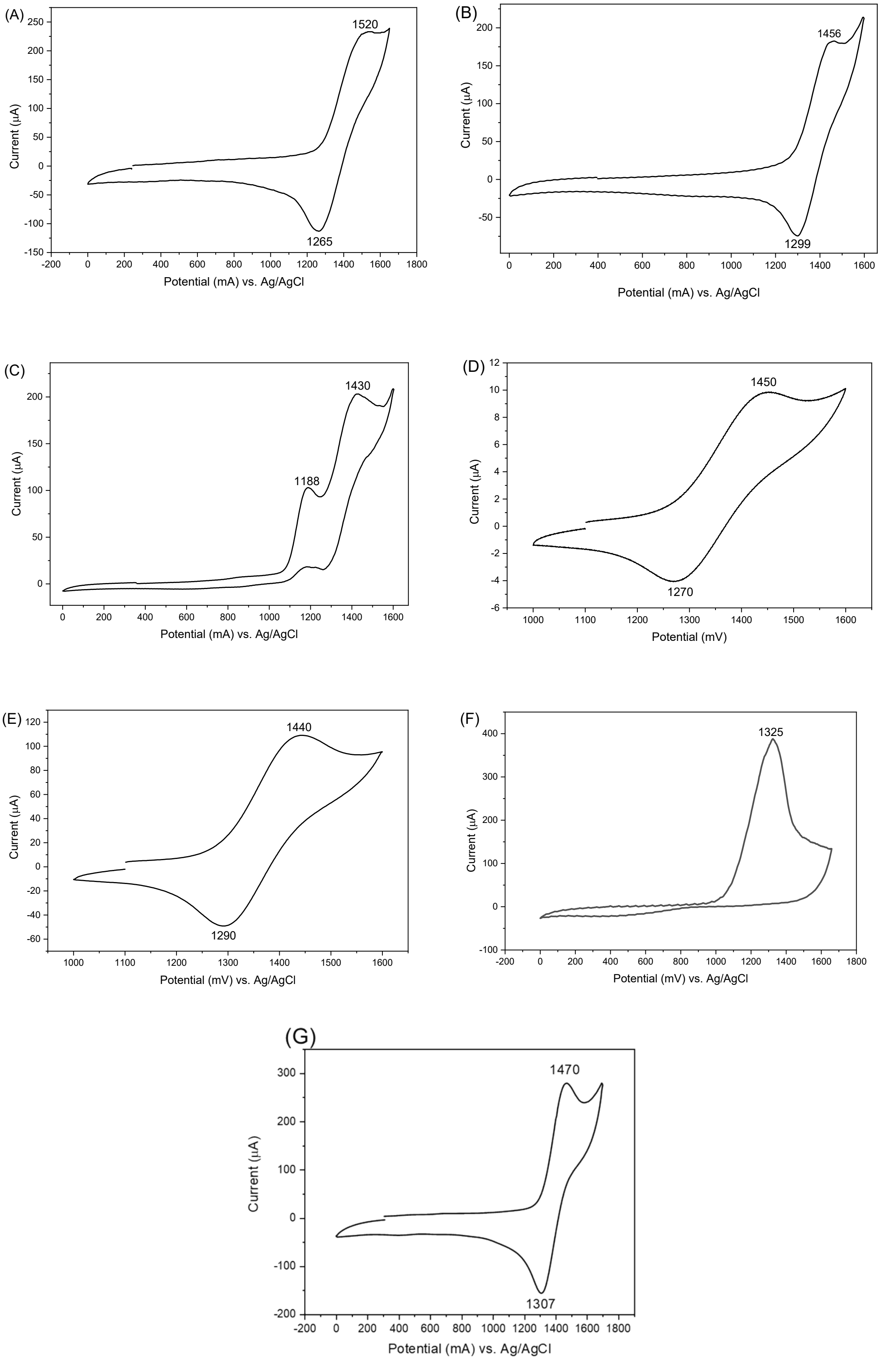

Figure S29. Cyclic voltammogram for complexes (A) **RuBi**, (B) **RuMo**, (C) **$RuNH_2$**, (D) **RuCCl**, (D) **RuCBr**, ligand (F) L-NH_2 and (G) **$RuNO_2$** in CH_2Cl_2 (Tetrabutylammonium perchlorate 0.1 M; Ag/AgCl; work electrode Pt; 100 mV s^{-1}).

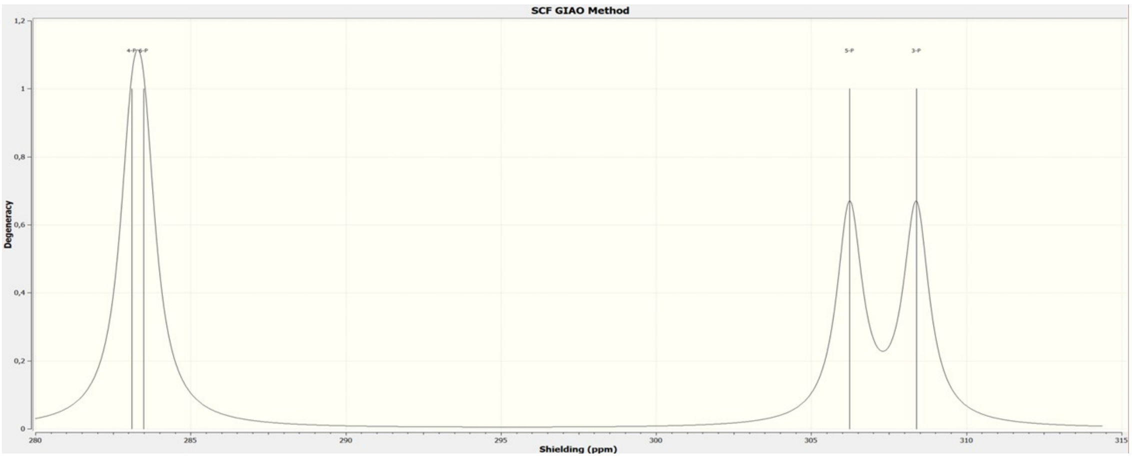

Figure S30. Simulated ^{31}P NMR spectrum for conformer 1. Calculated using the GIAO method at the B3LYP/def2-SVP level of theory. Four phosphorus signals are observed, with two nearly overlapping resonances indicating a pair of chemically equivalent P

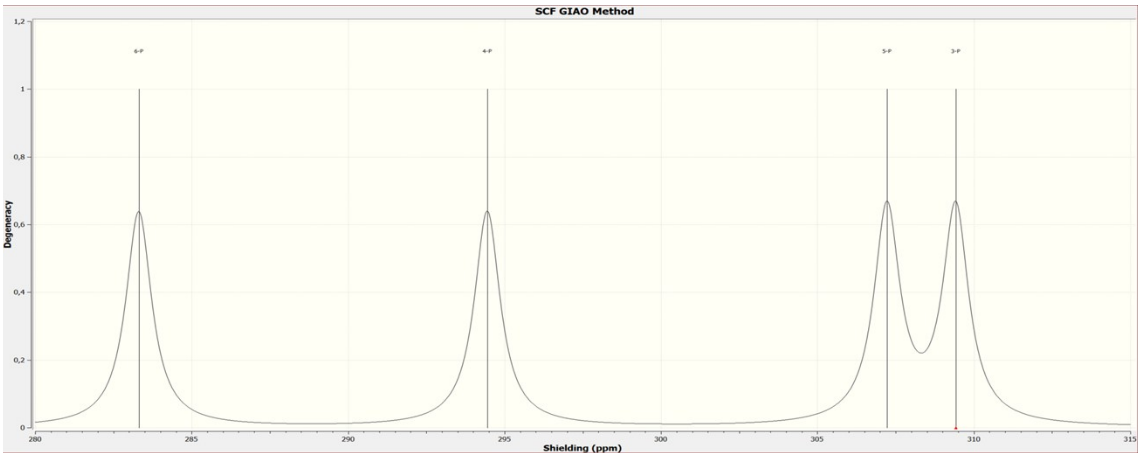

environments in conformer 1.

Figure 31. Simulated ^{31}P NMR spectrum for conformer 2. Calculated using the GIAO method at the B3LYP/def2-SVP level of theory. The spectrum shows four distinct phosphorus resonances consistent with inequivalent P environments in conformer 2.

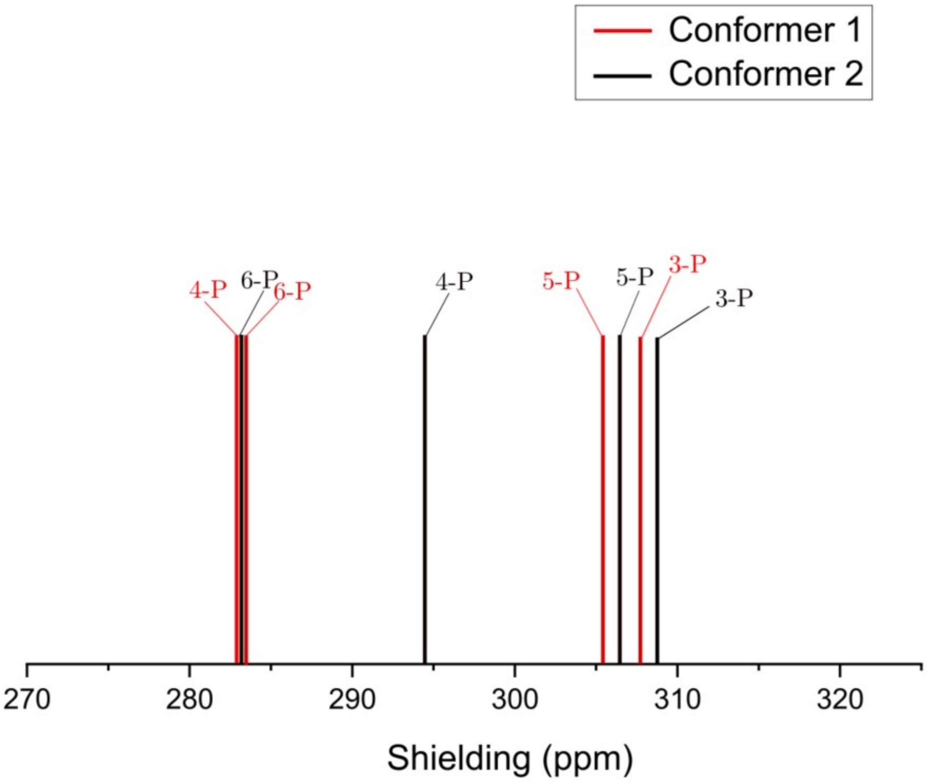

Figure S32. Superposition of the simulated ^{31}P NMR spectra for conformers 1 and 2. Comparison of the calculated chemical shielding values obtained using the GIAO method at the B3LYP/def2-SVP level of theory. Red peaks correspond to conformer 1 and black peaks to conformer 2, evidencing slight variations in the ^{31}P chemical environment between the two conformers.

A

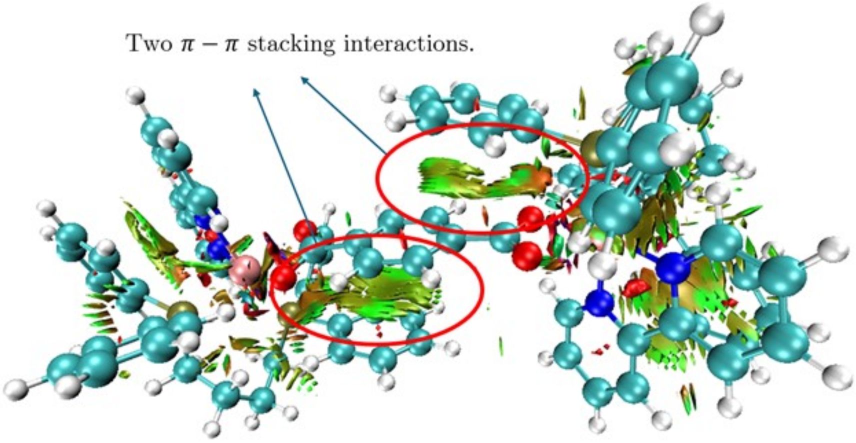

B

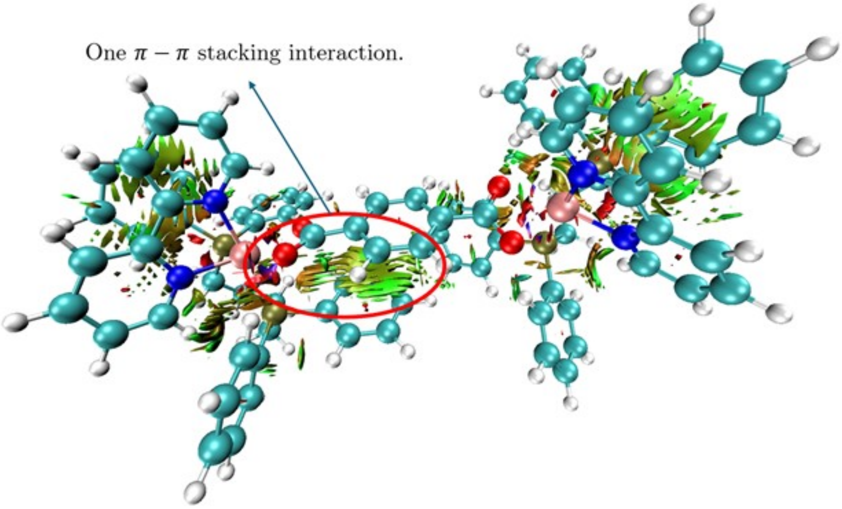

C

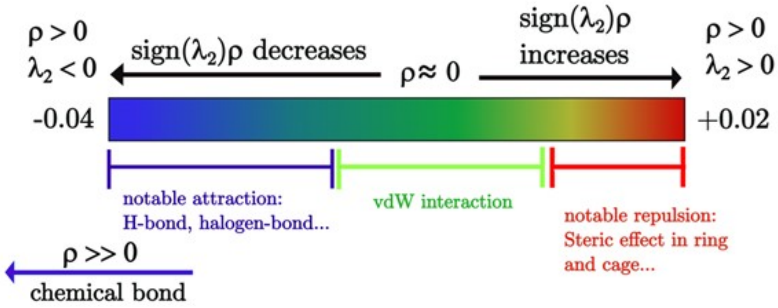

Figure S33. NCI analysis for the studied structures. (A) NCI isosurface showing two π–π stacking interactions between aromatic fragments in conformer 1. (B) NCI isosurface displaying a single π–π stacking interaction in conformer 2. (C) Colour scale for the NCI plots, where blue indicates attractive interactions, green corresponds to weak van der Waals contacts, and red denotes repulsive regions (sign(λ_2)ρ).

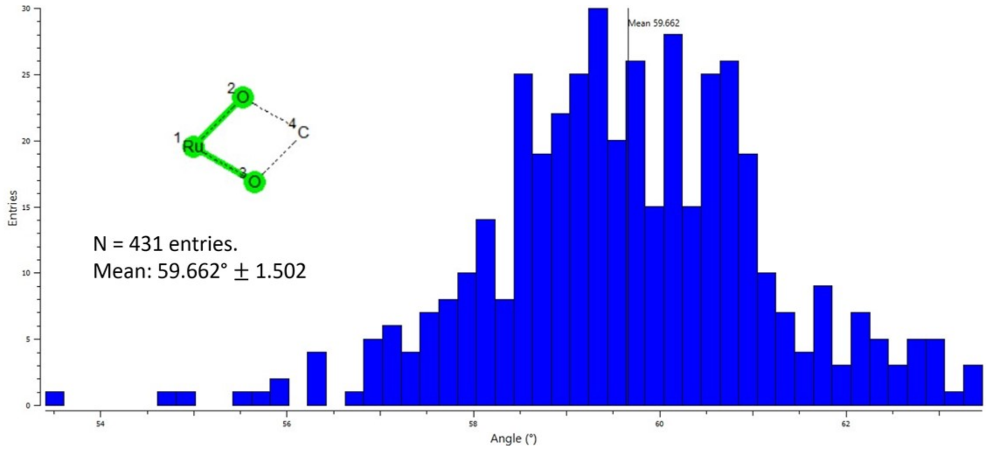

Figure S34. Values of 4-membered ring O-Ru-O angles over 431 entries in the Cambridge Structural Database (CSD).

Table S2. Crystal data and refinement details.

	**$RuNH_2$**	**RuCBr**	**RuBi**	**$RuNO_2$**
CCDC code	2487110	2487109	2487111	2513690
Empirical formula	$C_{46.25}H_{47}F_6N_3O_{3.25}P_3Ru$	C46H42.6BrF6 N2O2.3P3Ru	$C_{84}H_{76}F_{11.98}N_4O_6P_6Ru_2$	$C_{45}H_{40}F_6N_3O_4P_3Ru$
Formula weight /g mol^{-1}	1004.85	1.048.143	1857.548	994.78
Temperature /K	293(2)	100.1(3)	100.00(14)	100.0(3)
Crystal system	Triclinic	monoclinic	Monoclinic	triclinic
Space group	P-1	P21/c	I2/a	P-1
a/Å	11.6936(3)	15.7262(1)	29.52159(11)	12.8108(3)
b/Å	13.4117(4)	14.8624(1)	12.88956(6)	13.9903(2)
c/Å	15.3031(4)	19.3587(1)	43.88249(18)	14.5427(3)
α/°	106.669(2)	90	90	65.487(2)
β/°	97.953(2)	99.520(1)	96.7437(4)	66.110(2)
γ/°	90.324(2)	90	90	73.459(2)
Volume/$Å^3$	2274.43(11)	4462.38(5)	16582.65(12)	2146.91(9)
Z	2	4	8	2
ρ_{calc}g/cm^3	1.467	1.560	1.459	1.539
μ/mm^{-1}	0.519	5.486	4.721	4.652
F(000)	1029.0	2124	7446.8	1012.0
Crystal size/mm^3	0.105 X 0.048 X 0.012	0.12 × 0.09 × 0.04	0.21 X 0.11 X 0.05	0.09 × 0.07 × 0.03
Radiation	Mo Kα (λ = 0.71073)	Cu Kα (λ = 1.54184)	Cu Kα (λ = 1.54184)	Cu Kα (λ = 1.54184)
2Θ range for data collection/°	5.198 to 69.136	8.98 to 140.14	9.18 to 140.14	9.612 to 140.14
Index ranges	-18 ≤ h ≤ 18, -20 ≤ k ≤ 21, -24 ≤ l ≤ 23	-20 ≤ h ≤ 19, -18 ≤ k ≤ 18, -20 ≤ l ≤ 24	-29 ≤ h ≤ 37, -16 ≤ k ≤ 16, -55 ≤ l ≤ 55	-15 ≤ h ≤ 15, -17 ≤ k ≤ 16, -17 ≤ l ≤ 17
Reflections collected	74606	63786	96366	41358
Independent reflections	18179 [R_{int} = 0.0392, R_{sigma} = 0.0363]	8467 [Rint = 0.0283, Rsigma = 0.0210]	15726 [R_{int} = 0.0319, R_{sigma} = 0.0252]	8120 [R_{int} = 0.0472, R_{sigma} = 0.0397]
Data/restraints/parameters	18179/105/542	8467/373/799	15726/72/1043	8120/0/559
Goodness-of-fit on F^2	1.051	1.054	1.044	1.029
Final *R* indexes [*I* ≥ 2σ(*I*)]	R_1 = 0.0544, wR_2 = 0.1414	R1 = 0.0459, wR2 = 0.1313	R_1 = 0.0420, wR_2 = 0.1189	R_1 = 0.0415, wR_2 = 0.1130
Final *R* indexes [all data]	R_1 = 0.0853, wR_2 = 0.1685	R1 = 0.0476, wR2 = 0.1329	R_1 = 0.0448, wR_2 = 0.1211	R_1 = 0.0457, wR_2 = 0.1164
Largest diff. peak/hole/e $Å^{-3}$	1.23/-1.39	1.20/-3.59	1.65/-0.64	1.46/-0.99

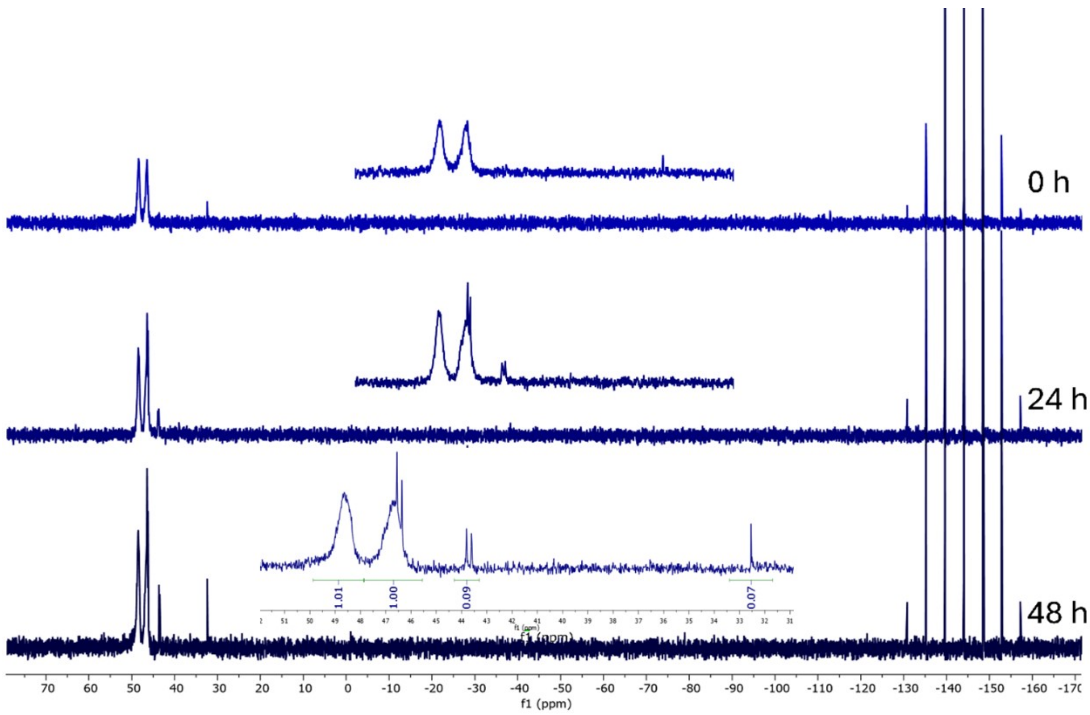

Figure S35. $^{31}P\{^{1}H\}$ NMR spectra of the complex **RuBi** in DMSO/10% RPMI culture medium, in 0, 24, and 48 h.

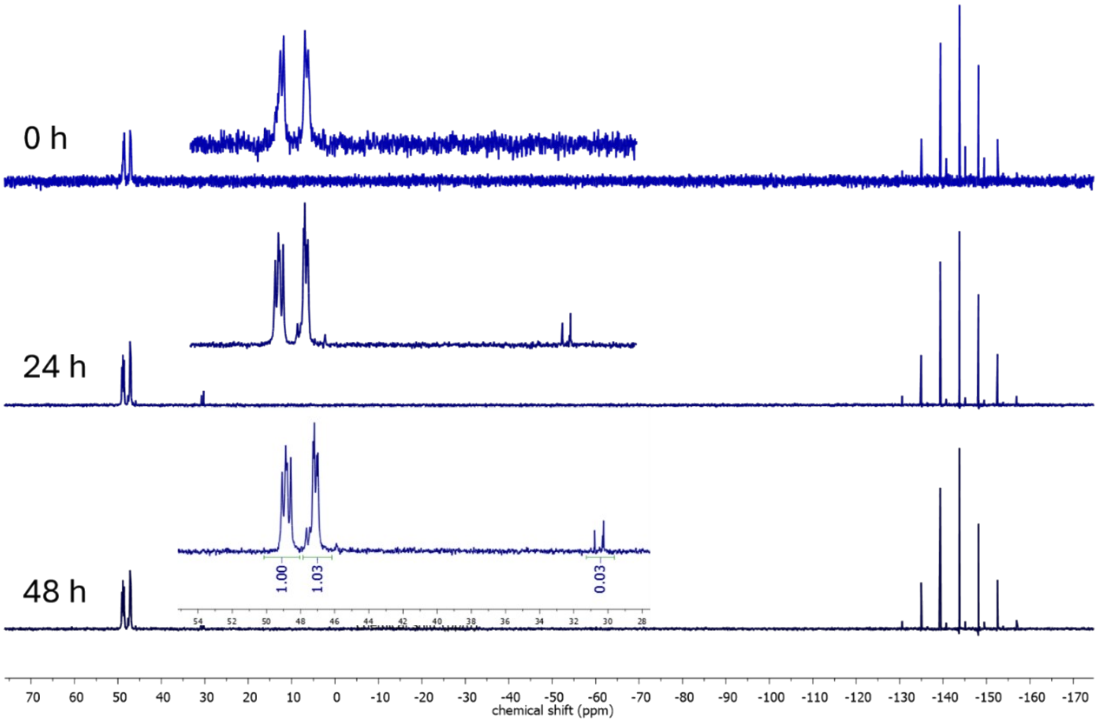

Figure S36. $^{31}P\{^{1}H\}$ NMR spectra of the complex **RuMo** in DMSO/10% RPMI culture medium, in 0, 24, and 48 h.

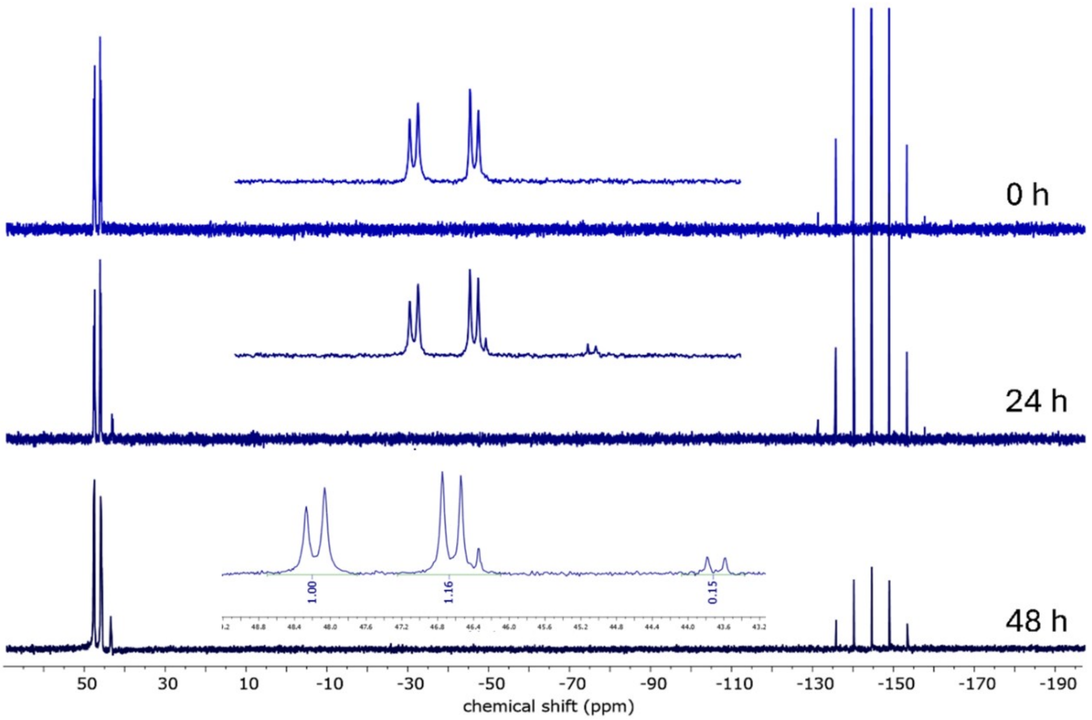

Figure S37. $^{31}P\{^1H\}$ NMR spectra of the complex **$RuNH_2$** in DMSO/10% RPMI culture medium, in 0, 24, and 48 h.

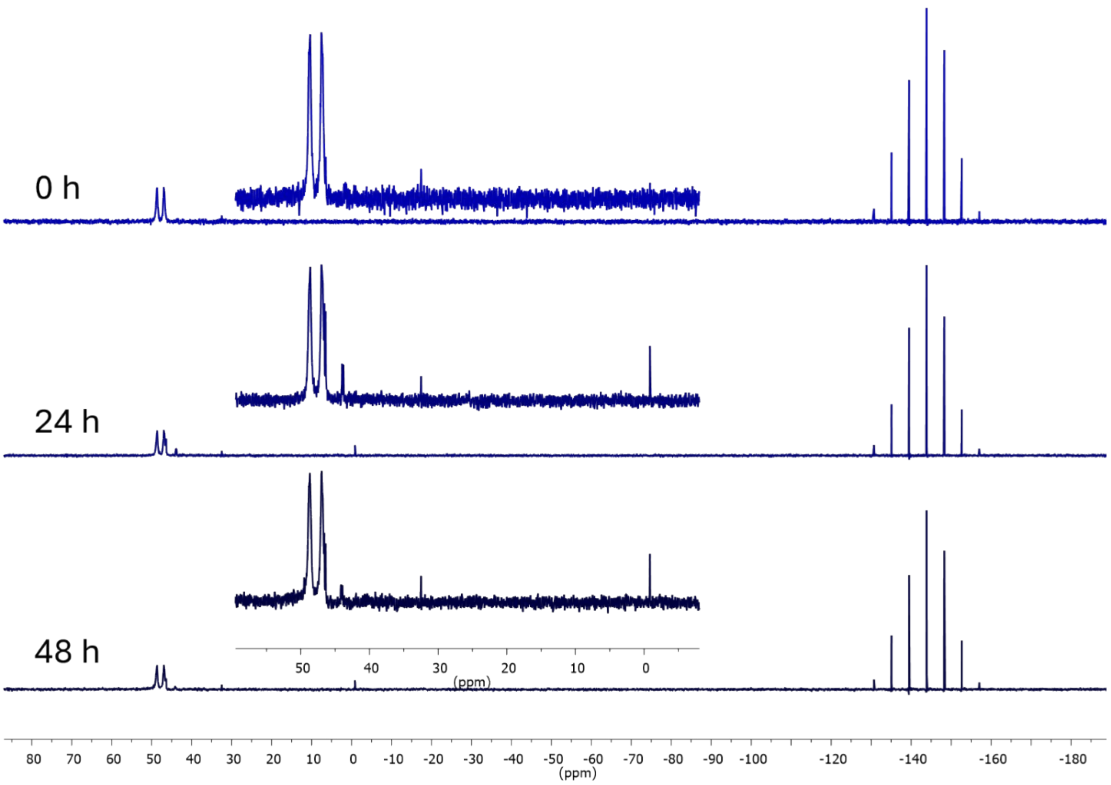

Figure S38. $^{31}P\{^1H\}$ NMR spectra of the complex **RuCCl** in DMSO/10% RPMI culture medium, in 0, 24, and 48h.

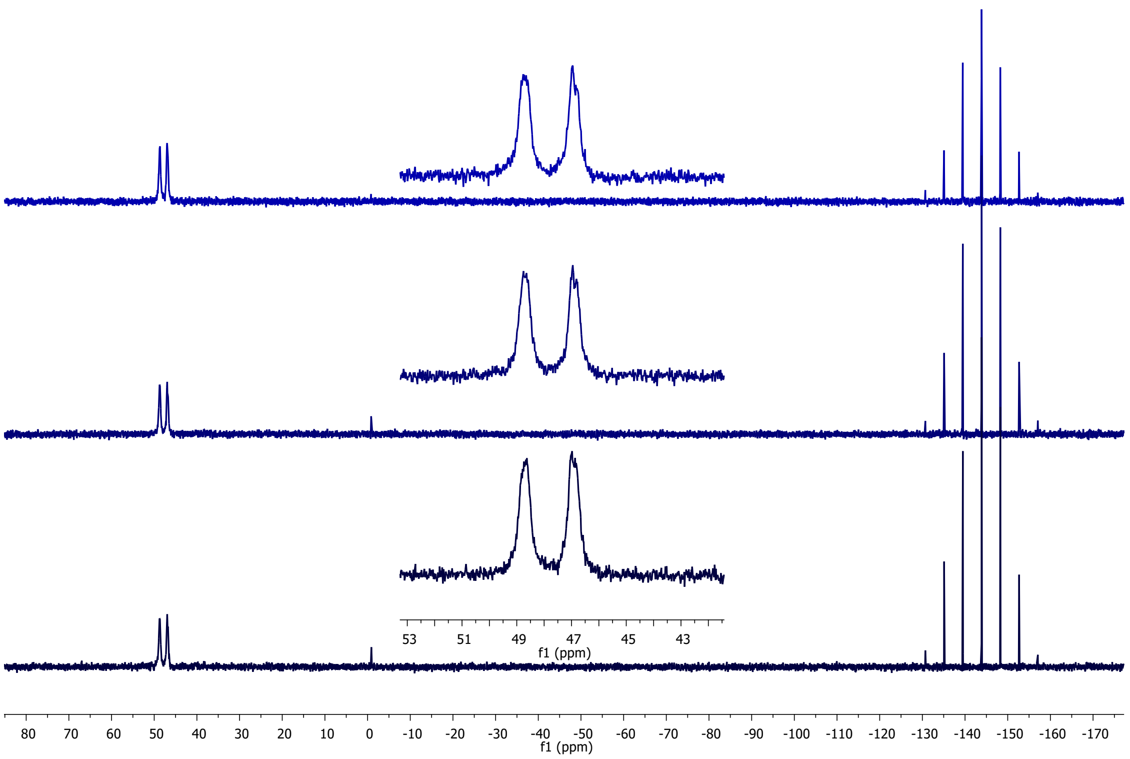

Figure S39. $^{31}P\{^{1}H\}$ NMR spectra of the complex **RuCBr** in DMSO/10% RPMI culture medium, in 0, 24, and 48h.

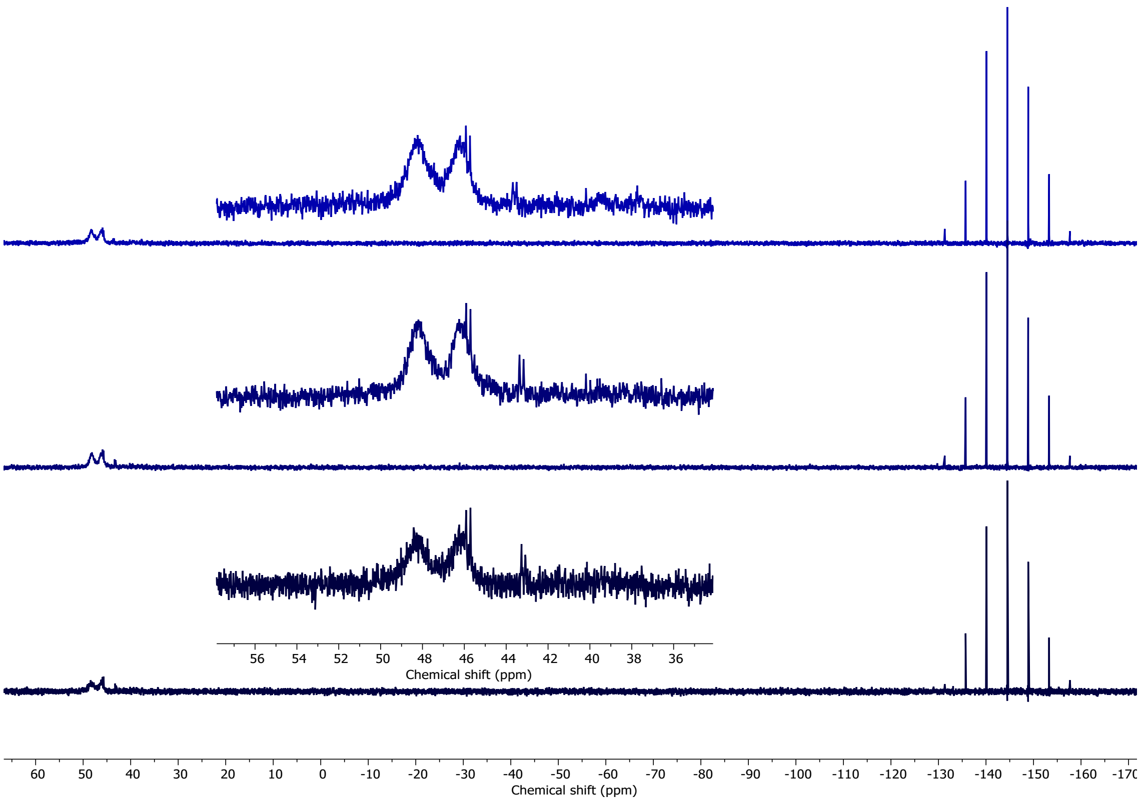

Figure S40. $^{31}P\{^{1}H\}$ NMR spectra of the complex **$RuNO_2$** in DMSO/10% RPMI culture medium, in 0, 24, and 48h.

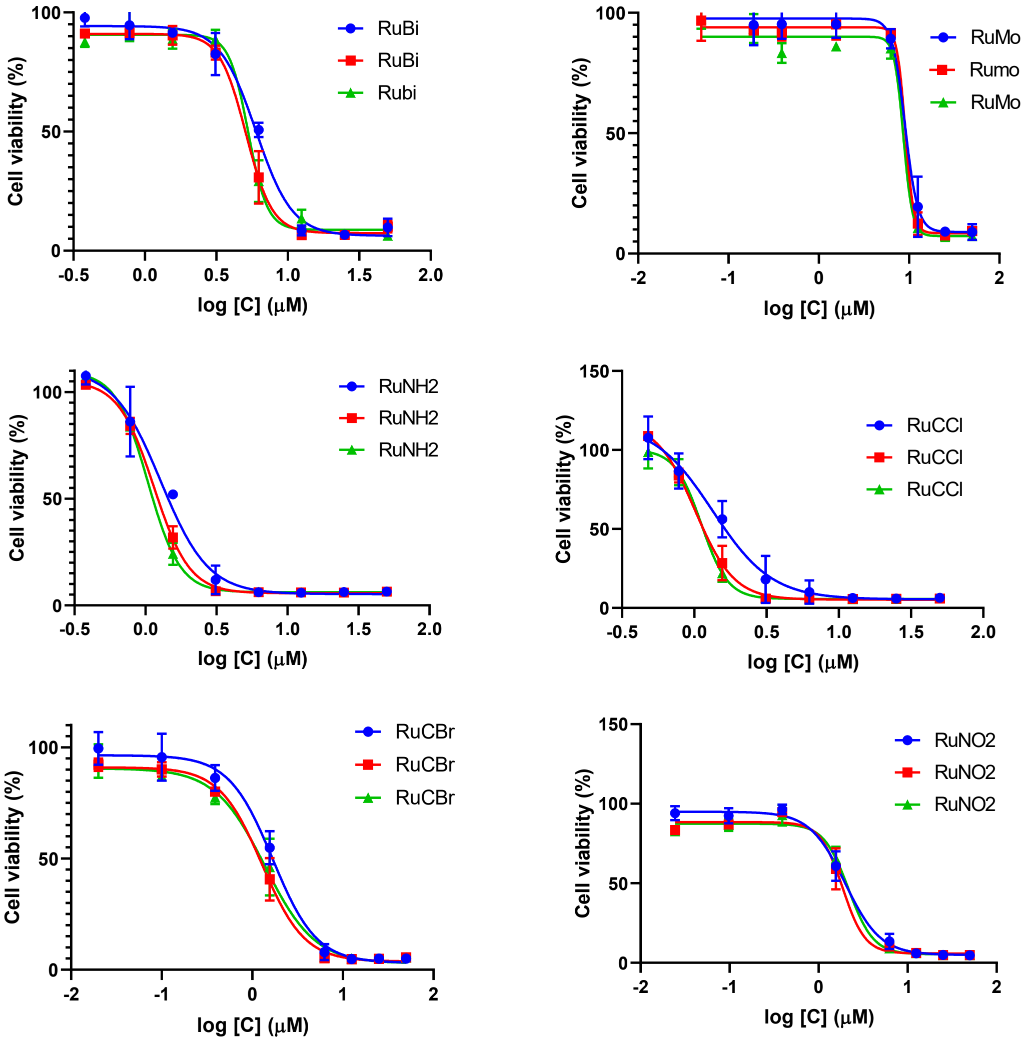

Figure S41. Concentration-response curve of tumor cells MDA-MB-231 after complexes treatment for 48 h, in triplicate.

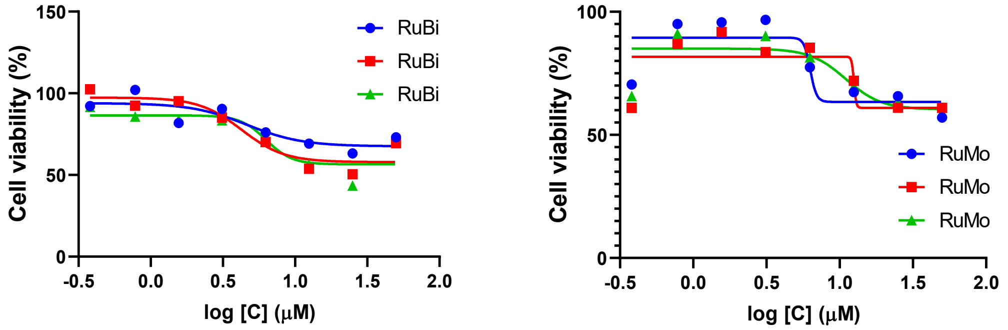

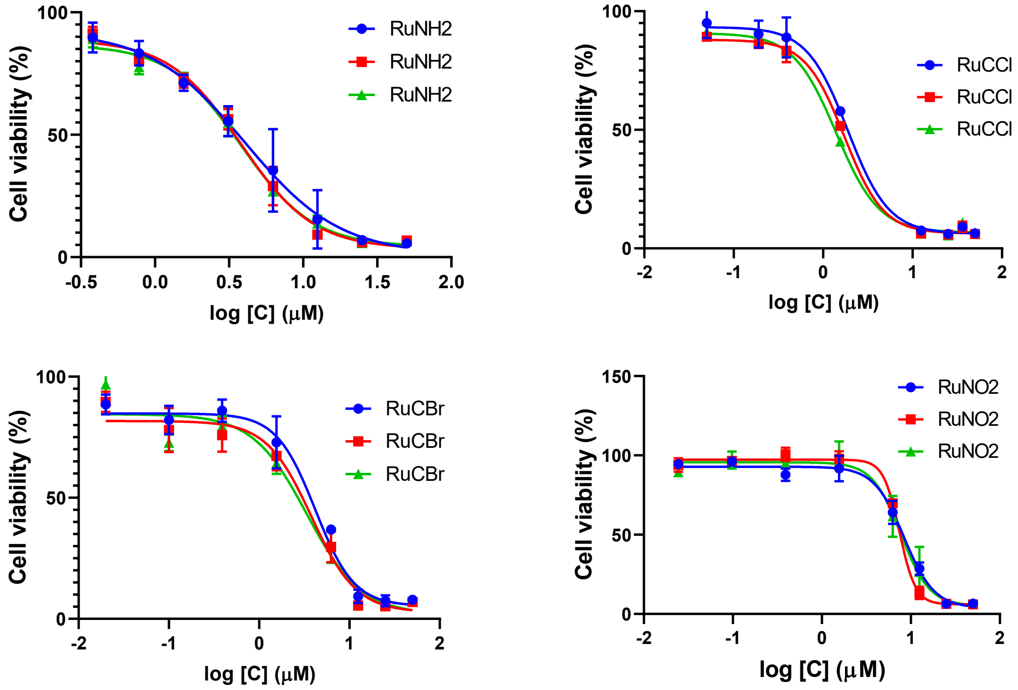

Figure S42. Concentration-response curve of tumor cells A549 after complexes treatment for 48 h, in triplicate.

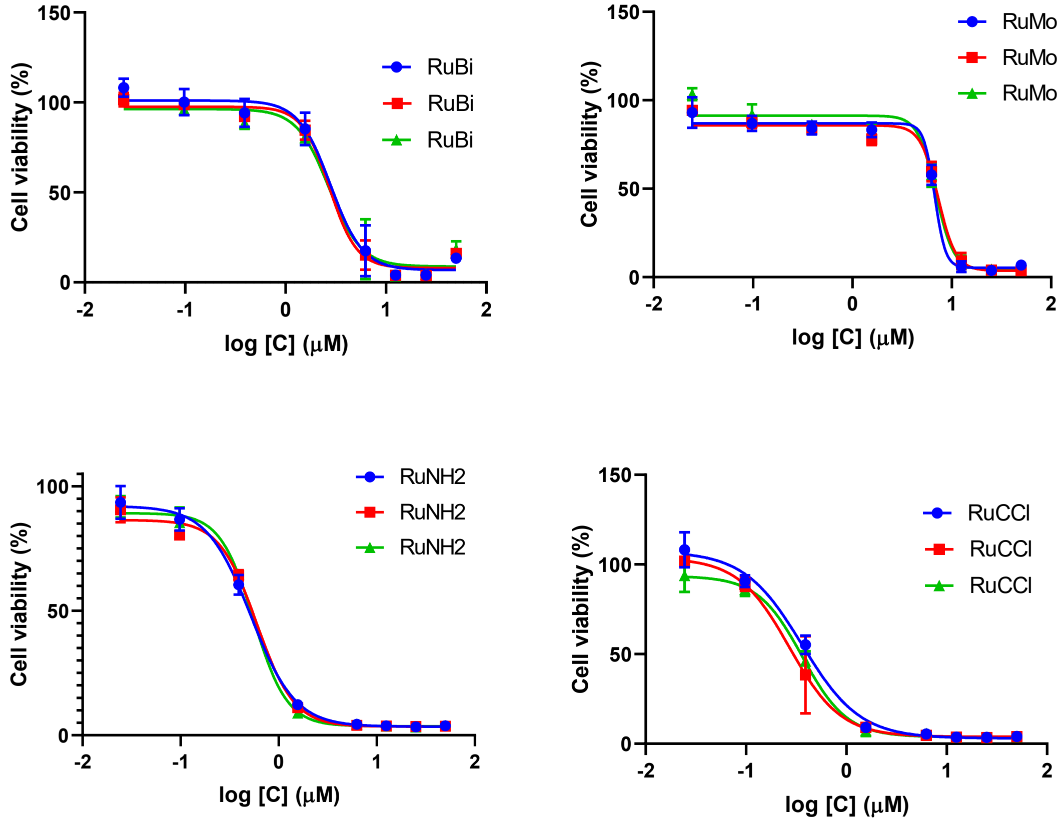

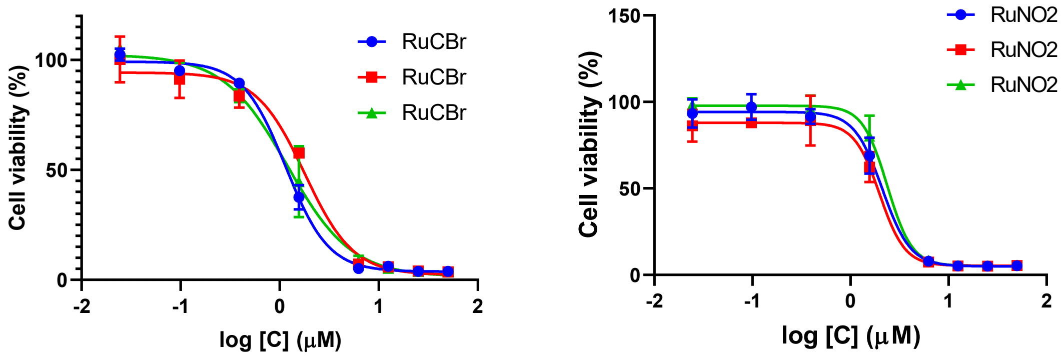

Figure S43. Concentration-response curve of tumor cells A2780 after complexes treatment for 48 h, in triplicate.

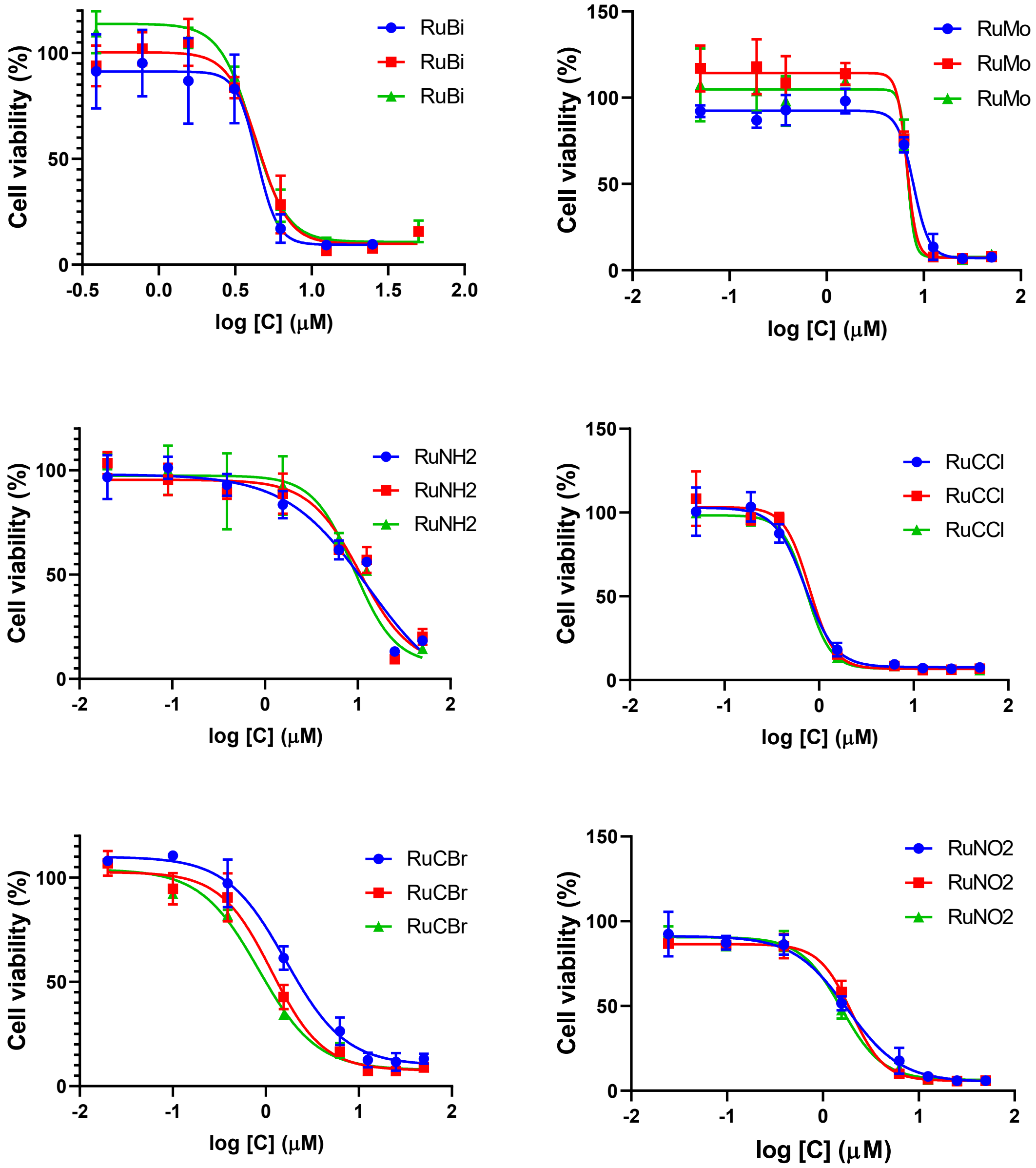

Figure S44. Concentration-response curve of tumor cells A2780cis after complexes treatment for 48 h, in triplicate.

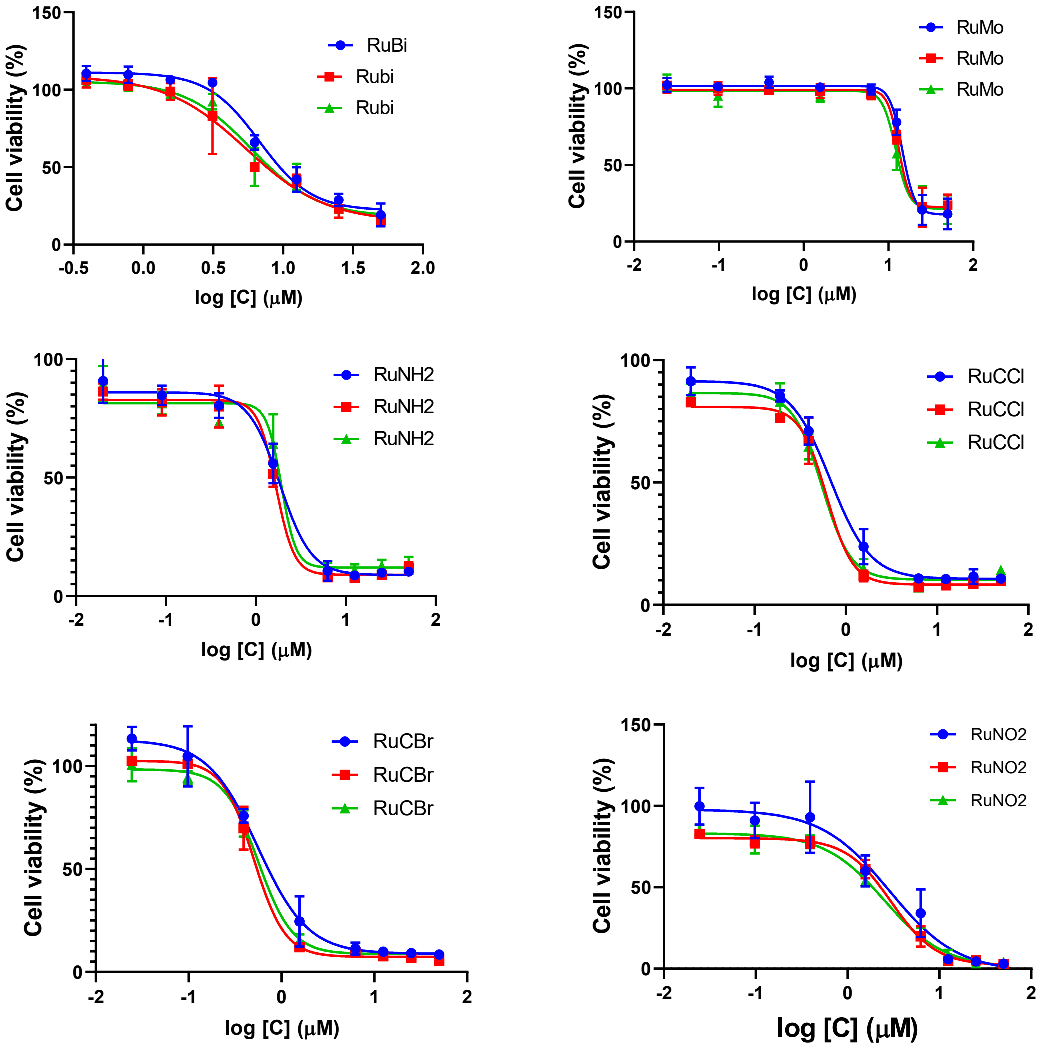

Figure S45. Concentration-response curve of non-tumor cells MRC-5 after complexes treatment for 48 h, in triplicate.

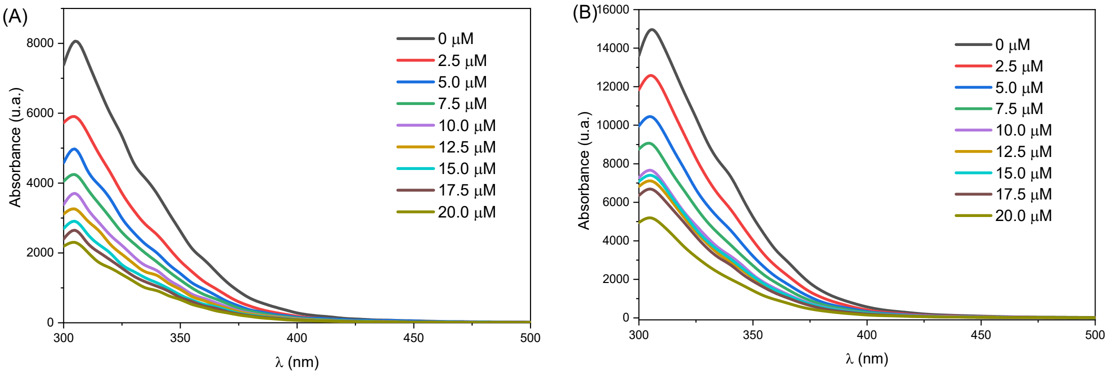

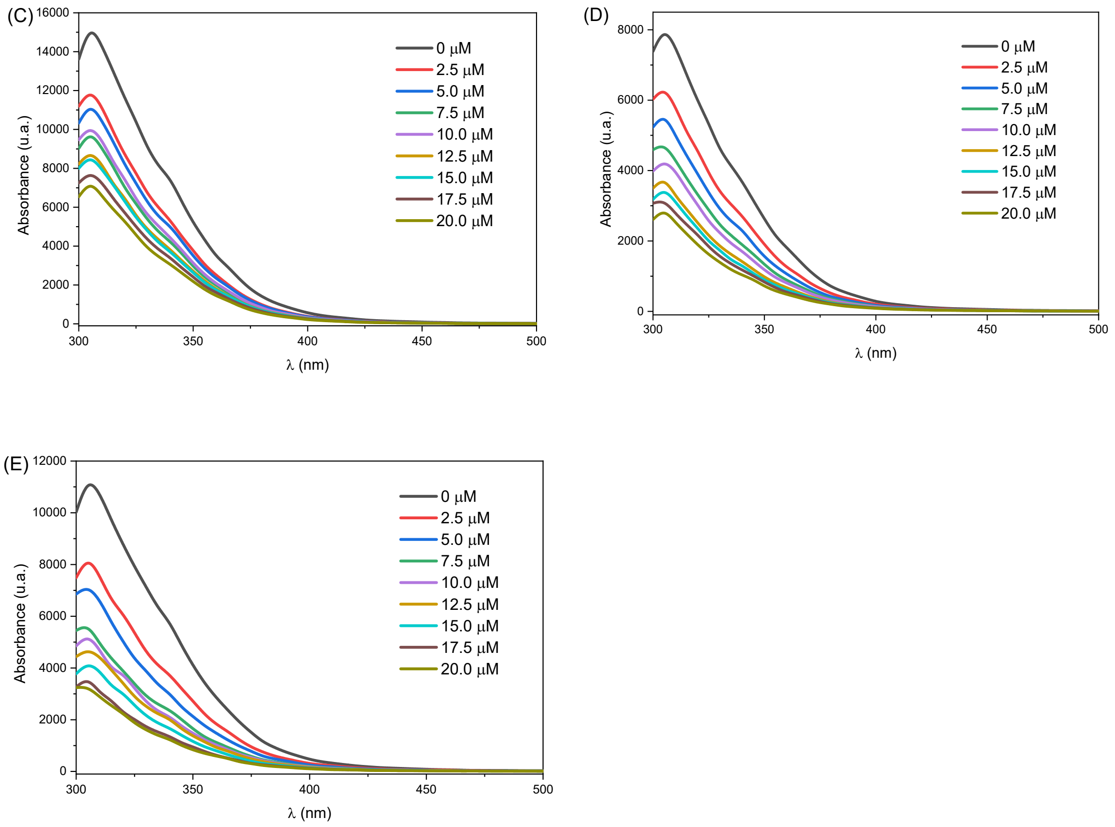

Figure S46. Fluorescence spectra of HSA solution (2.5 μM) in Tris–HCl buffer (0.1 M NaCl, pH 7.4) in the absence and presence of different concentrations of complexes (A) **RuBi**, (B) **RuMo**, (C) **RuCCl**, (D) **RuCBr** and (E) **$RuNO_2$**.

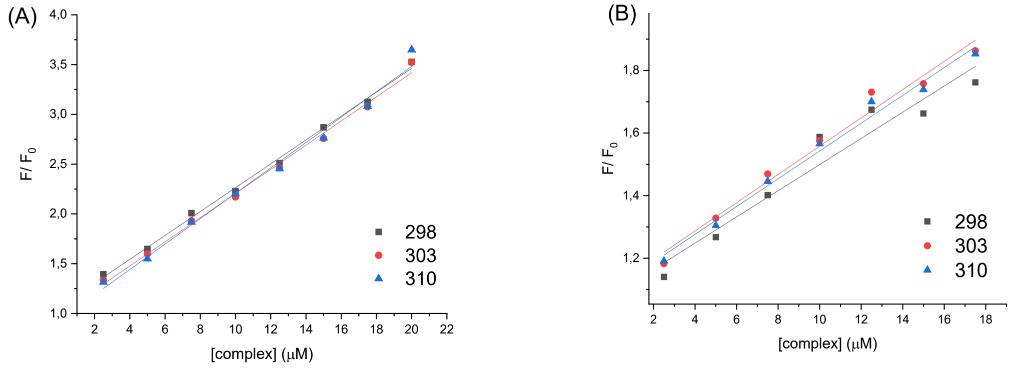

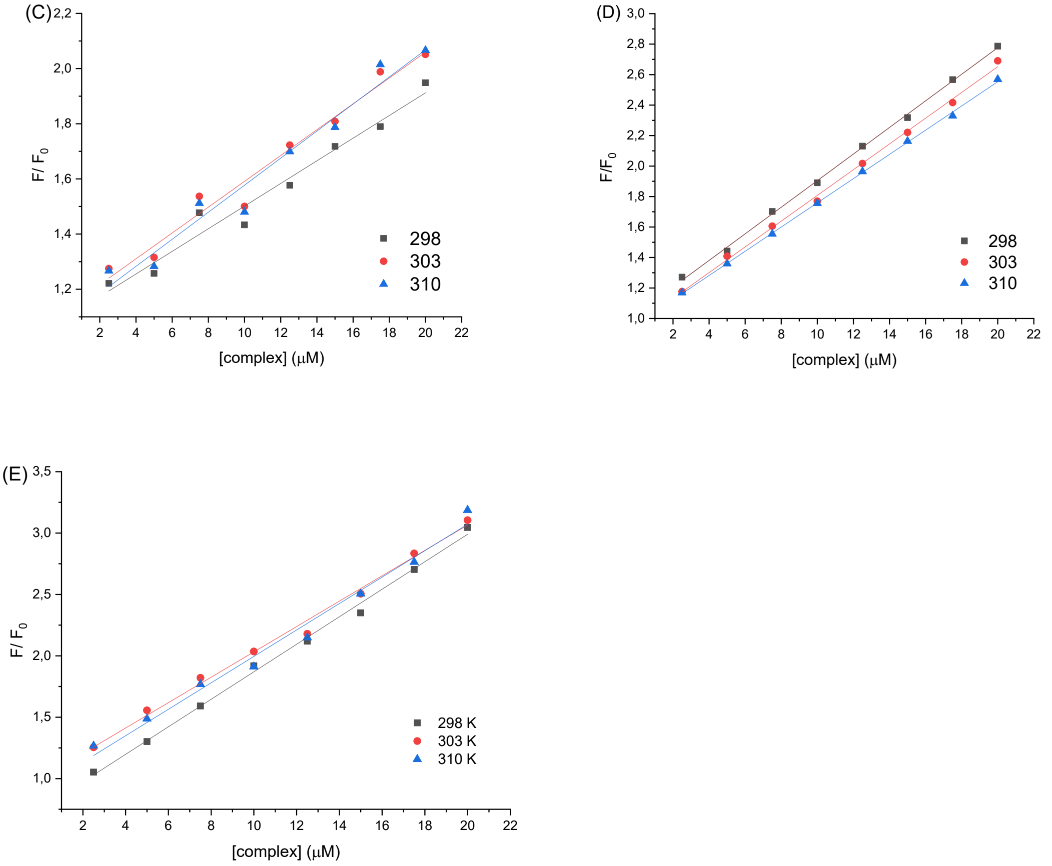

Figure S47. Stern–Volmer plots for the quenching of HSA fluorescence by complexes (A) **RuBi**, (B) **RuMo**, (C) **RuCCl** and (D) **RuCBr** and (E) **$RuNO_2$**, at 298, 303 and 310 K.
